# The association of *ARRB1* polymorphisms with response to antidepressant treatment in depressed patients

**DOI:** 10.3389/fphar.2022.974570

**Published:** 2022-10-26

**Authors:** Kenneth Chappell, Abd El Kader Ait Tayeb, Romain Colle, Jérôme Bouligand, Khalil El-Asmar, Florence Gressier, Séverine Trabado, Denis Joseph David, Bruno Feve, Laurent Becquemont, Emmanuelle Corruble, Céline Verstuyft

**Affiliations:** ^1^ Université Paris-Saclay, UMR 1018, CESP-Inserm, Team MOODS, Faculté de Pharmacie, Bâtiment Henri MOISSAN, Orsay, France; ^2^ Service Hospitalo-Universitaire de Psychiatrie de Bicêtre, Hôpitaux Universitaires Paris-Saclay, Assistance Publique-Hôpitaux de Paris, Hôpital de Bicêtre, Le Kremlin Bicêtre, France; ^3^ INSERM UMR-S U1185, Faculté de Médecine, University Paris-Saclay, Le Kremlin Bicêtre, France; ^4^ Service de Génétique Moléculaire, Pharmacogénétique et Hormonologie, Hôpitaux Universitaires Paris-Saclay, Assistance Publique-Hôpitaux de Paris, Hôpital de Bicêtre, Le Kremlin Bicêtre, France; ^5^ Department of Epidemiology and Population Health, Faculty of Health Sciences, American University of Beirut, Beirut, Lebanon; ^6^ CESP, MOODS Team, INSERM UMR 1018, Faculté de Médecine, University Paris-Saclay, Le Kremlin Bicêtre, France; ^7^ Sorbonne Université-INSERM, Centre de Recherche Saint-Antoine, UMR S938, Institut Hospitalo-Universitaire ICAN, Service d’Endocrinologie, Hôpital Saint-Antoine, Assistance Publique-Hôpitaux de Paris, Centre de Référence des Maladies Rares de l’Insulino-Sécrétion et de l’Insulino-Sensibilité, Paris, France; ^8^ Centre de Recherche Clinique Paris-Saclay, Hôpitaux Universitaires Paris-Saclay, Assistance Publique-Hôpitaux de Paris, Hôpital de Bicêtre, Le Kremlin Bicêtre, France; ^9^ Centre de Ressources Biologiques Paris-Saclay, Hôpitaux Universitaires Paris-Saclay, Assistance Publique-Hôpitaux de Paris, Hôpital de Bicêtre, Le Kremlin Bicêtre, France

**Keywords:** high-throughput sequencing, pharmacogenetics, major depressive disorder, β Arrestin, ARRB1

## Abstract

**Introduction:** β-arrestin 1, a protein encoded by *ARRB1* involved in receptor signaling, is a potential biomarker for the response to antidepressant drug (ATD) treatment in depression. We examined *ARRB1* genetic variants for their association with response following ATD treatment in METADAP, a cohort of 6-month ATD-treated depressed patients.

**Methods:** Patients (*n* = 388) were assessed at baseline (M0) and after 1 (M1), 3 (M3), and 6 months (M6) of treatment for Hamilton Depression Rating Scale (HDRS) changes, response, and remission. Whole-gene *ARRB1* variants identified from high-throughput sequencing were separated by a minor allele frequency (MAF)≥5%. Frequent variants (i.e., MAF≥5%) annotated by RegulomeDB as likely affecting transcription factor binding were analyzed using mixed-effects models. Rare variants (i.e., MAF<5%) were analyzed using a variant set analysis.

**Results:** The variant set analysis of rare variants was significant in explaining HDRS score changes (*T* = 878.9; *p* = 0.0033) and remission (*T* = -1974.1; *p* = 0.034). Rare variant counts were significant in explaining response (*p* = 0.016), remission (*p* = 0.022), and HDRS scores at M1 (*p* = 0.0021) and M3 (*p*=<0.001). rs553664 and rs536852 were significantly associated with the HDRS score (rs553664: *p* = 0.0055 | rs536852: *p* = 0.046) and remission (rs553664: *p* = 0.026 | rs536852: *p* = 0.012) through their interactions with time. At M6, significantly higher HDRS scores were observed in rs553664 AA homozygotes (13.98 ± 1.06) compared to AG heterozygotes (10.59 ± 0.86; *p* = 0.014) and in rs536852 GG homozygotes (14.88 ± 1.10) compared to AG heterozygotes (11.26 ± 0.95; *p* = 0.0061). Significantly lower remitter rates were observed in rs536852 GG homozygotes (8%, n = 56) compared to AG heterozygotes (42%, *n* = 105) at M6 (*p* = 0.0018).

**Conclusion:** Our results suggest *ARRB1* variants may influence the response to ATD treatment in depressed patients. Further analysis of functional *ARRB1* variants and rare variant burden in other populations would help corroborate our exploratory analysis. β-arrestin 1 and genetic variants of *ARRB1* may be useful clinical biomarkers for clinical improvement following ATD treatment in depressed individuals.

**Clinical Trial Registration:** clinicaltrials.gov; identifier NCT00526383

## 1 Introduction

Major Depressive Disorder (MDD) is the leading contributor to global disability (World Health Organization, 2017). Its main treatment option, antidepressant drugs (ATD), is only modestly effective, with more than 65% of ATD-treated patients failing to achieve remission (Trivedi et al., 2008). Given the rising global burden of MDD and the lack of effective treatments, it is necessary to identify robust biomarkers to predict treatment response.

Two potential candidates, β-arrestin 1 and 2, are ubiquitously expressed proteins encoded by the *ARRB1* and *ARRB2* genes, respectively (Parruti et al., 1993; Lefkowitz and Shenoy, 2005). Both have functions in receptor desensitization, especially with many G protein-coupled receptor (GPCR) families, though roles in protein scaffolding, clathrin-mediated endocytosis, and G-protein-independent signaling are known (Shukla et al., 2011; Bond et al., 2019). Indeed, the β-arrestins serve as scaffolds for various proteins, including the phosphoinositide-3, extracellular signal-regulated 1 and 2 (ERK1/2), and several mitogen-activated protein (MAP) kinases, whose signaling pathways are implicated in the treatment of mood disorders (Golan et al., 2009; Shukla et al., 2011; Bond et al., 2019). Additionally, β-arrestin 1 has been observed to function as a cytoplasm-to-nucleus messenger and nuclear protein scaffold (Golan et al., 2009). Interactions with GPCRs include serotonin, dopamine, adrenergic, and melatonin receptors, which are associated with different brain pathways and implicated in the pathophysiology of MDD (Kovacs et al., 2009; Petit et al., 2018).

Furthermore, biased agonists can preferentially activate downstream pathways. With respect to GPCRs, this includes classical G-protein- and/or β-arrestin-mediated pathways. Importantly, biased signaling may lend itself to the develop of novel therapeutic strategies in diverse pharmacological domains (Violin and Lefkowitz, 2007; Shukla et al., 2011; Bond et al., 2019). An example of biased agonism with respect to antidepressants was demonstrated with the tricyclic antidepressant, desipramine, a norepinephrine reuptake inhibitor and α2-adrenergic receptor ligand that was observed to lead to β-arrestin-mediated α2-adrenergic receptor internalization and downregulation in mouse embryonic fibroblasts (Cottingham et al., 2011).

**FIGURE 1:**
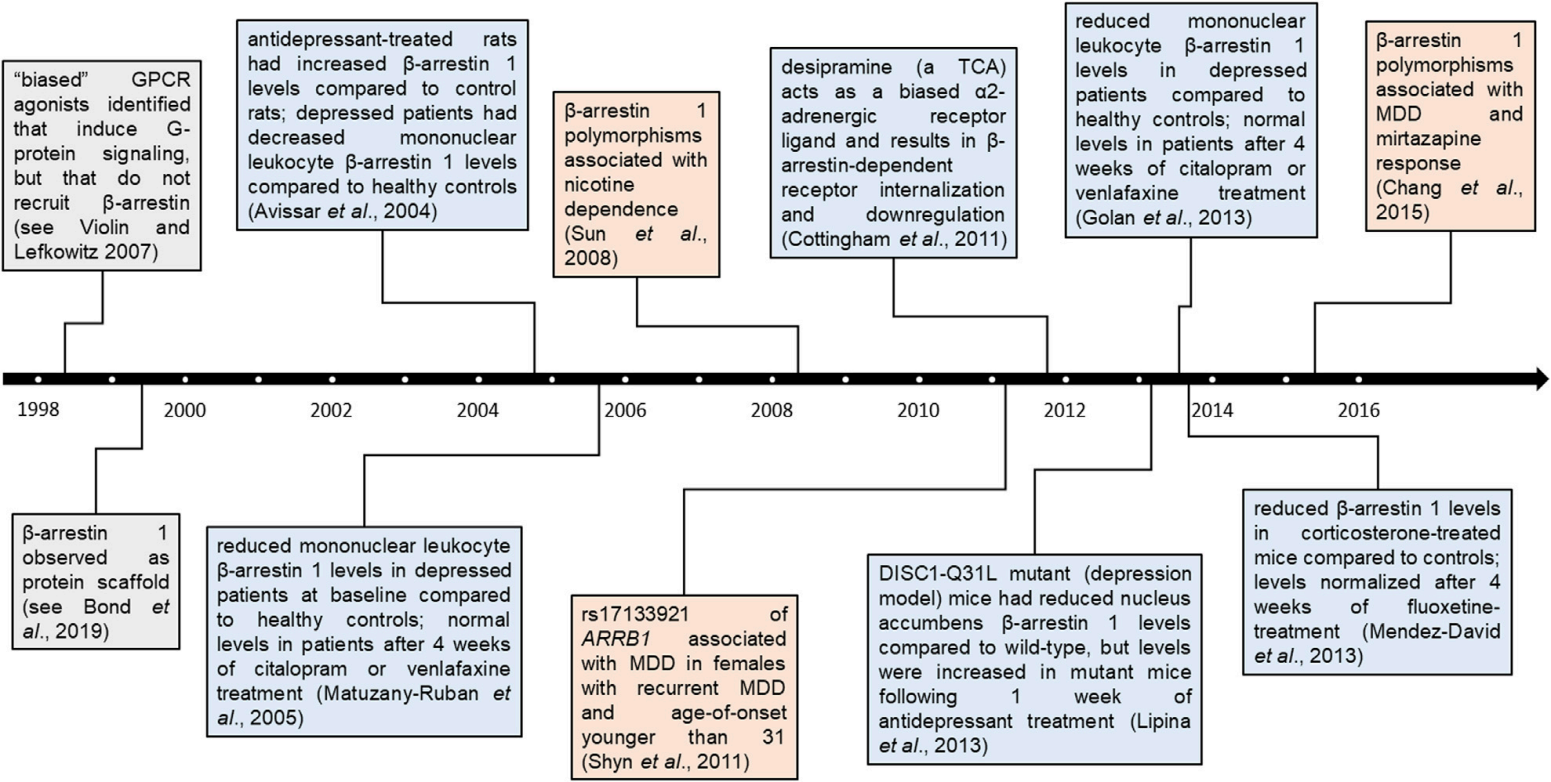
Timeline of β-arrestin 1 findings in psychopharmacology. Findings pertaining to β-arrestin 1 and *ARRB1* across time. Colored boxes denote broad β-arrestin 1 findings (grey), β-arrestin 1 protein findings in the context of depression and/or antidepressants (blue), or genetic associations in the context of neuropsychiatric disorders (orange). Distance from timeline denotes whether studies were conducted entirely in human participants (furthest), partly (between), or in other models (closest).

Lower *ARRB1* mRNA and β-arrestin 1 protein levels have been observed in murine models of depression, as well as in human mononuclear leukocytes of depressed patients, compared to healthy controls (Avissar et al., 2004; Matuzany-Ruban et al., 2005; Golan et al., 2013; Lipina et al., 2013; Mendez-David et al., 2013). These levels returned to levels similar to those of healthy controls in both murine models and depressed patients following 4 weeks of antidepressant treatment, a change that preceded clinical improvement (Golan et al., 2013). A timeline of studies examining β-arrestin 1 and *ARRB1* in the context of depression and its treatment with ATDs is summarized in Figure 1. These associations suggest that β-arrestin 1 may be a promising candidate biomarker for ATD treatment response in MDD patients (Mendez-David et al., 2013). Factors that may alter its expression or function, such as genetic variants, are thus of interest.

**FIGURE 2:**
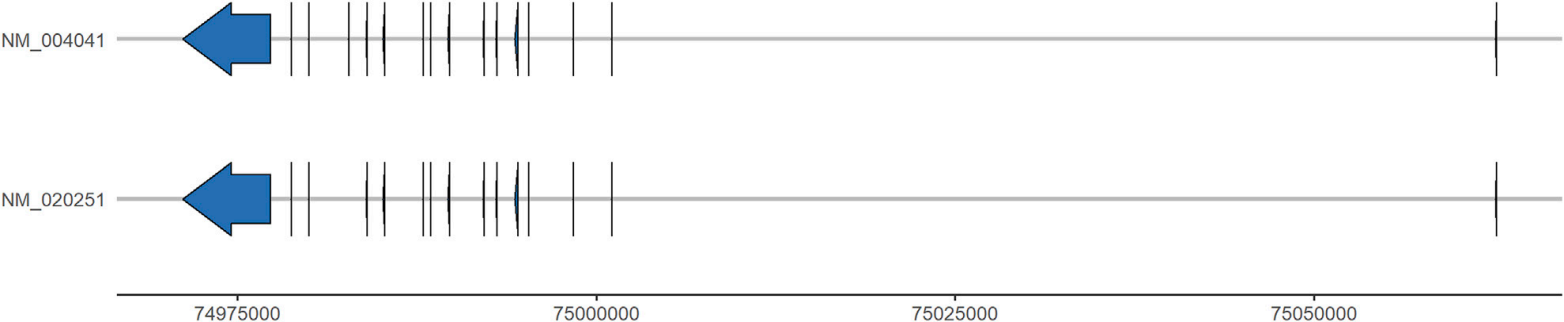
*ARRB1* transcript isoforms. Graphical representations of the two *ARRB1* transcript isoforms along chromosome 11 (genomic position, *x*-axis). Exons and 5′- and 3′-untranslated regions are represented by vertical lines and arrows. Intronic sequences are represented by the intervening horizontal lines. Data were obtained from NCBI Genome Data Viewer, assembly GRCh37. p13 (accessed 15 August 2022).

*ARRB1*, located on chromosome 11q13 and comprising 16 exons, encodes for two splicing isoforms of β-arrestin 1 (Parruti et al., 1993; Calabrese et al., 1994; O’Leary et al., 2016) (see Figure 2). Single nucleotide polymorphisms (SNP) of *ARRB1* have been studied principally in the neuropsychiatric contexts of nicotine dependence and depression (Sun et al., 2008; Chang et al., 2015). In a European cohort, a haplotype constructed from 6 SNPs within *ARRB1* was significantly associated with nicotine dependence (Sun et al., 2008). In a meta-analysis of 3 cohorts of depressed individuals of European ancestry, the intronic rs17133921 SNP was associated with MDD, specifically in the subgroup of females with recurrent MDD and an onset age of MDD prior to the age of 31 (Shyn et al., 2011). Furthermore, the rs12274033 SNP upstream of *ARRB1*, and a haplotype including it, were both associated with the response to mirtazapine treatment in a Korean population of MDD (Chang et al., 2015). These findings suggest that genetic polymorphisms of *ARRB1* may have an influence in different psychiatric contexts and could thus serve as potential candidate biomarkers for the response to ATD treatment in the context of MDD.

Advances in genetic analysis thanks to genome-wide association studies (GWAS) and high-throughput sequencing (HTS) technology have greatly increased the identification of genetic variants associated with disease. Furthermore, the Encyclopedia of DNA elements (ENCODE) project has mapped the vast majority of the human genome to functional elements involved in protein coding, transcription factor (TF) binding, and chromatin structure (The ENCODE Project Consortium, 2012). Importantly, these data can be used to annotate genetic variants with regulatory information and offer insight into their potential functional consequences in various disease contexts. RegulomeDB is one such database that uses these and other predictive data to rank genetic variants on their likelihood to lie in a functional element and thus have a functional consequence (Boyle et al., 2012).

However, most variants detected by HTS have a minor allele frequency (MAF) less than the 5% threshold principally used to delineate common variants (Momozawa et al., 2016). The analysis of these rarer variants is thus generally more difficult and underpowered. One approach to this issue is grouping variants into sets—defined by genes or genomic regions—and analyzing the association between this cumulative variable and a trait of interest (Chen et al., 2019). Recently, the optimal unified sequence kernel association test (SKAT-O) variant set method was used to identify significant associations between rarer variants in *ADH1C* and *GSTO1* and mRNA expression from a set of 303 pharmacokinetic-related genes (Klein et al., 2019). Importantly, SKAT-O considers both burden and SKAT tests, which groups variants with respect to the mean or the variance, respectively. It thus optimizes test selection depending on whether most variants act in the same direction (burden) or in opposing directions, i.e., both protectively and deleteriously (SKAT) (Lee et al., 2012).

Given that both β-arrestin 1 levels and *ARRB1* genetic variants have been associated with MDD and ATD treatment response, the objectives of this study were to analyze the association of clinical response following ATD treatment in a cohort of 6-month ATD-treated MDD patients, with 1) the set of rare variants and 2) frequent variants with likely functional consequences, identified from exonic, intronic, and 5′- and 3′-untranslated regions of *ARRB1*.

## 2 Materials and methods

### 2.1 Study design

Do Antidepressants Induce Metabolic Syndromes (METADAP) is a 6-month prospective, multicentric, and observational cohort study carried out in a psychiatric setting (Corruble et al., 2015). Patients with a current major depressive episode (MDE) in the context of MDD were treated in naturalistic conditions and assessed before and after beginning a new ATD treatment. This study was registered by the French National Agency for Medicine and Health Products Safety (ANSM) and the Commission Nationale de l'Informatique et des Libertés (CNIL). It was approved by the Ethics Committee of Paris-Boulogne (France) and conformed to international ethical standards (ClinicalTrials.gov identifier: NCT00526383).

### 2.2 Patient population

Males and females 18–65 years of age without a serious medical condition were recruited. Inclusion criteria were presentation with a current MDE in the context of MDD (DSM-IVTR), as assessed by the Mini International Neuropsychiatric Interview (MINI) and a score ≥18 on the 17-item Hamilton Depression Rating Scale (HDRS) (Hamilton, 1960), as well as need for a new ATD treatment. Patients with psychotic symptoms or other mental disorders—such as psychotic disorder, bipolar disorder, alcohol or drug dependence, or an eating disorder—or those who were pregnant or who had organic brain syndromes or serious medical conditions, were excluded. Patients using antipsychotics or mood stabilizers before inclusion and/or for at least 4 months during the year before inclusion were also excluded. Concurrent use with antipsychotics, mood stabilizers, and/or stimulants was not allowed during the study. Benzodiazepines, at the minimum effective dose and for the minimum time period, and psychotherapies were allowed (Corruble et al., 2015). Measures and samples were obtained prior to beginning ATD treatment (M0), and after 1 month (M1), 3 months (M3), and 6 months (M6) of ATD treatment. Data were collected from November 2009 to March 2013 in 6 university hospital (CHU) psychiatry departments across France in Paris (CHU Fernand-Widal and CHU Saint-Antoine), Le Kremlin-Bicêtre (CHU Bicêtre), Grenoble (CHU Grenoble Alps), Lille (CHU Michel Fontan 1), and Besançon (CHU of Besançon) (Corruble et al., 2015).

Of the 643 patients included in METADAP, 19 had major protocol deviations and were excluded. Of the 624 available for analysis, 519 patients provided samples for genetic studies. From these, 400 have undergone HTS. Two samples were removed due to technical difficulties. As our objective was to examine clinical response following ATD treatment, 10 patients without follow-up measures (i.e., at M1, M3, and M6) were removed. Thus, 388 patients were analyzed. Genetic analyses may have included fewer than 388 patients if overall call quality was high, but individual calls were poor and removed (discussed further below). The reasons for dropout included loss to follow-up (*n* = 87, 48.6%), ATD changes (*n* = 75, 41.9%), use of unauthorized drugs, including antipsychotics, mood stabilizers, and stimulants (*n* = 8, 4.5%), the presence of an exclusion criterion (i.e., unstable medical condition, psychiatric disorder, substance abuse, or pregnancy) during follow-up (*n* = 8, 4.5%), or death (*n* = 1, 0.5%). Missing data at M1 (*n* = 21) included 8 dropouts (38.1%; 4 lost to follow-up and 4 due to ATD changes), while missing data at M3 (*n* = 121) included 110 dropouts (90.9%; 62 lost to follow-up, 36 due to ATD changes, 6 due to unauthorized drug use, 5 due to the presence of an exclusion criterion, and 1 due to death. Information about ancestry was self-reported. Caucasian ethnicity was defined as having Caucasian parents, African as having Sub-Saharan African and/or Afro-Caribbean parents, and Asian as having East Asian, Central Asian, and/or South Asian parents (Morales et al., 2018). Three Asian patients were present within the analyzed population and were combined with the seven patients of mixed ethnicity. Education was classified into 3 levels: primary [e.g., elementary (≤5 years)]; secondary [e.g., middle school and high school (>5 and ≤12 years)]; tertiary [e.g., any college- or university-level education (>12 years)]. All patients provided written informed consent for study participation and for genetic analyses.

### 2.3 Antidepressant treatment

ATD monotherapies were prescribed by a psychiatrist in a “real world” psychiatric treatment setting as previously described (Corruble et al., 2015). ATDs belonged to 1 of 4 classes: selective serotonin reuptake inhibitors (SSRI), serotonin norepinephrine reuptake inhibitors (SNRI), tricyclic antidepressants (TCA), or other ATD treatments. In this ancillary study of 388 patients, 40% were prescribed an SSRI, 41% an SNRI, 6% a TCA, and 9% another ATD treatment; 4% received electroconvulsive therapy (ECT) instead of an ATD monotherapy. If a change in treatment was required during follow-up, the patient was dropped from the study.

### 2.4 Assessment of antidepressant treatment response

The HDRS was used to assess depression severity at M0 (i.e., baseline) and response to treatment at M1, M3, and M6. Responders to treatment were classified by an improved HDRS score of ≥50% relative to baseline, remitters by a HDRS score ≤7 after at least 4 weeks of treatment, as recommended by the American College of Neuropsychopharmacology (ACNP) Task Force (Rush et al., 2006). Clinical assessments were performed blind to genotyping results. Each interview and diagnostic assignment was reviewed by a senior psychiatrist. For each patient, all visits were reviewed by the same psychiatrist.

### 2.5 High-throughput sequencing and sequence alignment

Patient DNA samples were sequenced using a targeted panel of genes involved in mood disorders and ATD metabolism. Sequencing of the whole *ARRB1* gene was performed, including exons, introns, and 5′- and 3′-untranslated regions. The HTS protocol and variant calling methods are as previously described (Bouali et al., 2017; Chappell et al., 2021).

### 2.6 Variant filtration and selection pipeline

Variant Call Format data were loaded into R (v4.1.0) (R Core Team, 2020) using the vcfR package (v1.9.0) (Knaus and Grünwald, 2017). Variant calls were annotated for call quality. Specifically, variant calls with a sequencing depth (DP) < 20, SNPs with a quality score (QUAL) < 275, insertions/deletions (indels) with a QUAL<770, heterozygous calls with an allele balance (AB) < 0.34 or >0.79, and homozygous calls with an AB<0.96, were annotated as poor-quality calls. Variants with a call rate (# poor-quality calls/# of calls) < 95% were removed (Anderson et al., 2010). Frequent and rare variants were defined by a MAF≥5% or <5% (corresponding to both low-frequency and rare variants (Momozawa et al., 2016), but defined here as “rare” for simplicity), respectively. Frequent variants with a RegulomeDB category ranking of 1 or 2, corresponding to variants likely to directly affect TF binding—and in the case of rank 1, linked to expression of a gene target (Boyle et al., 2012)—were prioritized for individual analysis in association with clinical measures.

### 2.7 Variant set analysis

A variant set analysis was performed on all rare variants passing quality control (QC) using the *SMMAT* function from the GMMAT package (v1.3.2), which accounts for repeated measures in longitudinal data and can analyze both continuous and binary variables (Chen et al., 2019). The variables to be explained were the HDRS score, response rate, and remission rate. A null model was constructed including age, sex, ATD class, and visit (i.e., time) as covariables. Gaussian distributions with an identity link function were used to model the HDRS score, while binomial distributions with a logit link function were used to model response and remission rates. The genomic relationship matrix required by the *SMMAT* function was constructed using the *snpgdsGRM* function of the SNPRelate package (v1.26.0) (Zheng et al., 2012). The *SMMAT* function was run using default settings, except that the “use.minor.allele” argument was set to True—corresponding to the use of the minor allele as the coding allele rather than the alternative allele—and the “MAF.range” was set to 0.00-0.50. The SKAT-O test, corresponding to a linear combination of the burden and SKAT statistics, was used (Lee et al., 2012). As a single genomic region was analyzed, a threshold of *p* < 0.05 was considered significant.

### 2.8 Haplotype analysis and functional annotation of prioritized variants

Hardy-Weinberg equilibrium (HWE) and linkage disequilibrium (LD) were assessed using Haploview (v4.2) in the subgroup of Caucasian patients comprising 91% of the patient population (Barrett et al., 2005). Variants were considered to be in LD and assigned to a haplotype block as defined by an *r* ^2^ ≥ 0.8. The genetic variant with the highest MAF from each haplotype block was selected as a proxy for further analysis. Genetic variants were analyzed according to an additive model. Data from RegulomeDB (v2.0.3) and HaploReg (v4.1), including LD information, chromatin state, bound proteins, and altered TF motifs obtained from sources including the 1000 Genomes Project, ENCODE, and JASPAR, were used to annotate variants (Boyle et al., 2012; Ward and Kellis, 2016). LD between analyzed variants and undetected variants or variants outside of our sequencing range were further assessed using LDLink (Machiela and Chanock, 2015).

### 2.9 Data analysis

Statistical analyses were performed in R (v4.1.0) (R Core Team, 2020). Quantitative variables were analyzed using nonparametric Kruskal–Wallis rank-sum tests, while qualitative variables were analyzed using Fisher Exact tests. Mixed-effects models were used to analyze these longitudinal data. Importantly, missing data (e.g., due to dropout) does not result in a complete loss of information as these models allow for an average estimate to be calculated from the non-missing data (Mallinckrodt et al., 2003). Linear mixed-effects models and generalized linear mixed-effects logistic models were constructed with the lme4 package (v1.1-27.1) (Bates et al., 2015). The main variable to explain was the HDRS score. Other variables to explain were response and remission rates. Age, sex, ATD class, and visit were included *a priori* as fixed-effect covariables in all models. Demographic variables that significantly differed between genotype groups (i.e., *p* < 0.05) were also added as fixed-effects covariables. Individual was added as the random effect to account for repeated measures. Rare variant counts, variant genotype, and its interaction with visit, were the main explanatory variables examined. Rare variant counts were calculated by summing the number of MAF<5% variants present (i.e., heterozygous or homozygous genotype) in each individual. Given the exploratory nature of our study, we considered *p* < 0.05 as significant (Bender and Lange, 2001). Significance of fixed effects was assessed with the Satterthwaite method in linear mixed-effects models and with the Wald chi-square test in generalized linear mixed-effects models. In the event of a significant main or interaction effect, *post hoc* comparisons were carried out by performing linear and logistic regressions at individual timepoints (i.e., M1, M3, and M6) or by using the emmeans package (Lenth, 2022). Briefly, models were input into the *emmeans* function (with the “type” argument set to response) to estimate emmeans (for the HDRS) and probabilities (for response and/or remission) according to variant genotypes after controlling for other covariables. Comparisons were performed with the *contrast* function (with the “method” argument set to pairwise and the “infer” argument set to True) using the *emmeans* output.

## 3 Results

### 3.1 Patient demographics

Sociodemographic characteristics for the whole cohort are shown in Table 1. The mean age was 45.4, 68% of subjects were women, and 91% of subjects were Caucasian. 38% were current smokers at baseline and 73% had previously experienced an MDE.

**TABLE 1:** Sociodemographic characteristics. Sociodemographic characteristics of METADAP patients are shown for the whole sample. Kruskal–Wallis tests were used to compare age, tobacco consumption, onset age of MDE, and baseline HDRS scores (presented as mean ± standard deviation (m±sd)). Fisher Exact tests were used to compare sex, socio-education status, ethnicity, smoking status at baseline, MDE recurrence, prescribed ATD drug, and dropout rates across study time (presented as the number of patients and percentage). *: *p* < 0.05; **: *p* < 0.01; ***: *p* < 0.001. ATD, antidepressant drug; ECT, electroconvulsive therapy; HDRS, 17-item Hamilton Depression Rating Scale; m, mean; M1, after 1 month of treatment; M3, after 3 months of treatment; M6, after 6 months of treatment; MDE, major depressive episode; n, number of patients; *p*: *p*-value; SNRI, serotonin norepinephrine reuptake inhibitor; sd, standard deviation; SSRI, selective serotonin reuptake inhibitor; TCA, tricyclic antidepressant.

		Total sample *n* = 388
Age (in years) (m±sd)		45.4 ± 13.3
Female [*n* (%)]		263 (68)
Education [*n* (%)]	*Primary*	36 (9)
	*Secondary*	168 (43)
	*Tertiary*	183 (47)
Ethnicity [*n* (%)]	*Caucasian*	353 (91)
	*African*	24 (6)
	*Mixed*	10 (3)
Current smoker [*n* (%)]		149 (38)
Pack years (m±sd)		15.2 ± 14.9
Recurrent MDE [*n* (%)]		284 (73)
Onset age MDE (m±sd)		35.3 ± 14.5
Baseline HDRS (m±sd)		24.8 ± 4.9
Prescribed ATD [*n* (%)]	*SSRI*	157 (40)
	*SNRI*	158 (41)
	*TCA*	24 (6)
	*Other*	34 (9)
	*ECT*	15 (4)
Missing at follow-up [*n* (%)]	*M1*	21 (5)
	*M3*	121 (31)
	*M6*	179 (46)

### 3.2 Genetic variant selection and linkage analysis

**FIGURE 3:**
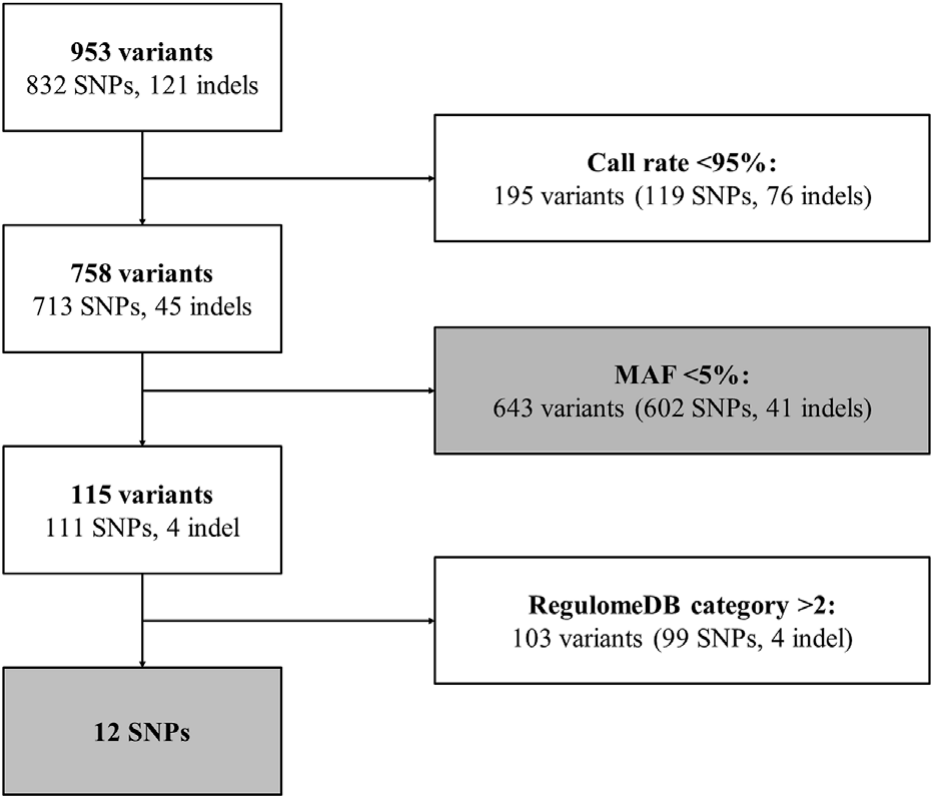
*ARRB1* genetic variant selection pipeline. Flowchart for *ARRB1* genetic variant selection. Gray boxes correspond to analyzed variants. Indel, insertion/deletion; MAF, minor allele frequency; SNP, single nucleotide polymorphism.

Genetic variant selection is summarized in Figure 3. A total of 953 unique genetic variants were identified from extracted Variant Call data, of which 832 were SNPs and 121 were indels (see Supplementary Table S1). 195 variants with call rates <95% (119 SNPs and 76 indels) were removed. Thus, 758 variants were available for analysis. 643 variants were rare (i.e., MAF<5%) (602 SNPs and 41 indels), of which 610 had a MAF<1% and 415 were identified in 1 individual (see Supplementary Table S1). Among the 115 frequent variants (i.e., MAF≥5%) (111 SNPs and 4 indels), 12 SNPs had a RegulomeDB category rank of 1 or 2 [i.e., linked to expression of a gene target (rank 1) and likely to directly affect TF binding (ranks 1 and 2)] and were prioritized for individual analysis (see Figure 3). Information for each prioritized SNP is shown in Table 2. Prioritized SNPs did not significantly deviate from HWE. Ten of the twelve prioritized SNPs were intronic, eight of which were located in intron 1.

**TABLE 2:** Genomic and other information about the 12 prioritized frequent SNPs. For the prioritized SNPs (i.e., MAF≥5% and RegulomeDB category rank of 1 or 2), the rs#, genomic position relative to the human genome assembly, hg19, and genomic region are shown. RegulomeDB rankings correspond to likely to affect binding and linked to expression of a gene target (1b, 1f) and likely to affect binding (2b, 2c). The major and minor alleles and minor allele frequency (MAF (%)) with respect to the Caucasian subpopulation are also shown. Hardy-Weinberg equilibrium *p*-values (HWE), haplotype blocks (Haplotype block), and linkage disequilibrium *r* ^2^ values (LD (*r* ^2^)) relative to the Caucasian subpopulation are also given. HWE, Hardy-Weinberg equilibrium; MAF, minor allele frequency; LD, linkage disequilibrium.

rs #	Position	Region	RegulomeDB rank	Major	Minor	MAF (%)	HWE	Haplotype block	LD (*r* ^2^)
rs2279130	74977185	3′UTR	2b	C	T	7.2	0.5		
rs501372	74993593	intron 5	2b	C	A	38.2	0.24	ht1	0.96
rs877711	74994352	exon5	2b	G	A	11.8	0.18		
rs567807	75000189	intron 2	2b	A	G	37.2	0.13	ht1	
rs553664	75005209	intron 1	2b	G	A	44.4	0.58	ht2	0.98
rs506233	75012535	intron 1	1f	A	G	43.7	0.38	ht2	
rs536852	75017436	intron 1	2b	G	A	49.1	0.98		
rs1676887	75019296	intron 1	1f	G	A	10.4	0.38		
rs113636971	75033020	intron 1	2b	A	G	6.4	0.88		
rs569796	75050842	intron 1	1b	G	C	29.9	0.48	ht3	0.98
rs504683	75051233	intron 1	2c	A	G	30.2	0.39	ht3	
rs561923	75058004	intron 1	2b	C	A	25.4	0.8		

Among the 12 prioritized SNPs, 3 pairs were observed in LD. rs501372 and rs567807 comprised haplotype block 1 (ht1), rs553664 and rs506233 (ht2), and rs569796 and rs504683 (ht3) (see Table 2 and Supplementary Figure S1). rs501372, rs553664, and rs504683 were selected as proxy SNPs for further analysis from each haplotype block. Nine SNPs were thus analyzed.

### 3.3 Variant set and accumulation analyses of rare variants

**FIGURE 4:**
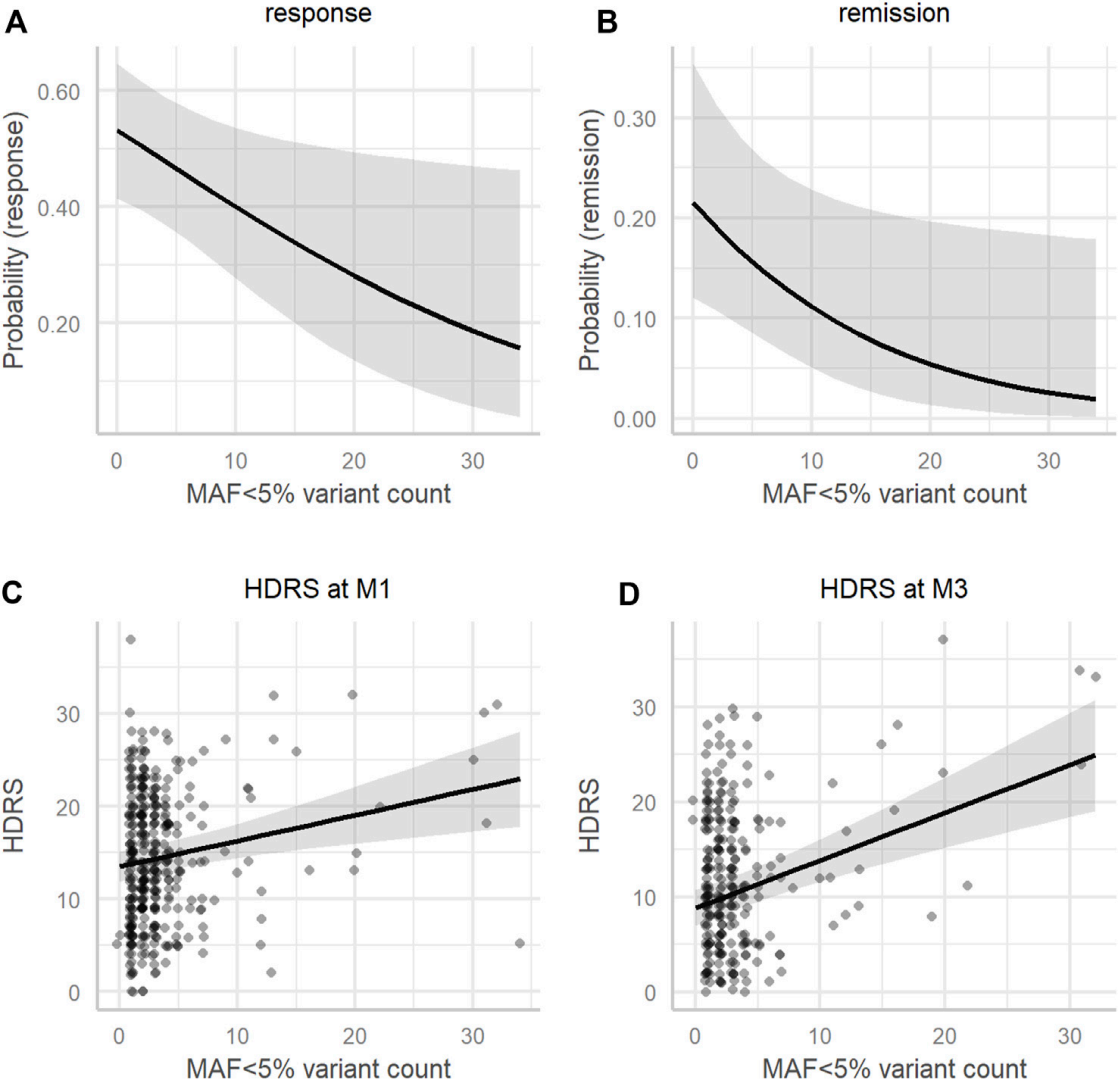
Association of MAF < 5% variant count with clinical measures. Model estimates adjusted for the mean age (45.36), sex (male), and antidepressant class (SSRI) of **(A)** the probability of response, **(B)** the probability of remission, **(C)** the HDRS score at M1, and **(D)** the HDRS score at M3 (*y*-axis) according to the MAF<5% variant count (*x*-axis). HDRS, 17-item Hamilton Depression Rating Scale; M1, 1 month after beginning antidepressant treatment; M3, 3 months after beginning antidepressant treatment; MAF, minor allele frequency.

*SMMAT* was used to assess the association of the 643 rare (i.e., MAF<5%) variants with clinical measures over time. The 643 rare variants were significant in explaining changes in the HDRS score over time in burden (*T* = 878.9; *Φ* = 89194.6; *p* = 0.0033) and SKAT-O tests (*p* = 0.0069) and remission rates in the burden test (*T* = -1974.1; *Φ* = 869281.1; *p* = 0.034), but not response rates, though a statistical trend was observed in the burden test (*T* = −1773.9; *Φ* = 937912.7; *p* = 0.07) (see Table 3). To further analyze the associations of rare variant accumulation with clinical measures, rare variant counts were calculated and analyzed. After controlling for age, sex, ATD class, and visit, a significant main effect of the rare variant count was observed in the mixed-effects models of response [χ^2^ (df = 1, *n* = 388) = 5.81, *p* = 0.016] and remission [χ^2^ (df = 1, *n* = 388) = 4.56, *p* = 0.033], as was a significant interaction with visit in the mixed-effects model of the HDRS score (*F*_3,918_ = 3.88, *p* = 0.0090) (see Table 4). In simplified mixed-effects models (i.e., without interaction) controlling for the same factors, the rare variant count was indeed significant in models of response (odds ratio (OR) = 0.95; 95% confidence interval (95%CI) [0.91–0.99]; *p* = 0.016) and remission (OR = 0.92; 95%CI [0.86–0.99]; *p* = 0.022) (see Figures 4A,B). Following Bonferroni correction, it was also a significant factor explaining the HDRS score in multiple linear regressions at M1 (coefficient = 0.26; 95%CI [0.11–0.41]; *p* = 0.0021) and M3 (coefficient = 0.46; 95%CI [0.28–0.64]; *p*=<0.001) (see Figures 4C,D), but not at M6 (coefficient = 0.25; 95%CI [−0.01–0.51]; *p* = 0.19).

**TABLE 3:** SMMAT results. Data and results for variant set analyses of the association between rare (i.e., MAF<5%) variants passing quality control and HDRS scores, response rates, and remission rates. All models included age, sex, antidepressant class, and visit as covariables. Shown are the mean allele frequency, the burden test score statistic and variance, and the associated *p*-values of the burden, SKAT, and SKAT-O tests. bold text*: *p* < 0.05. AF, allele frequency; *p*: *p*-value.

Clinical measure	Minimum frequency	Mean frequency	Maximum frequency	Burden stat	Burden variance	Burden *p*	SKAT *p*	SKAT-O *p*
*HDRS*	0.00129	0.00442	0.5	878.9	89194.6	0.0033*	0.41	0.0069*
*Response*				−1773.9	937912.7	0.07	0.47	0.12
*Remission*				−1974.1	869281.1	0.034*	0.37	0.06

**TABLE 4:** Mixed-effects model results. Associations for each of the clinical measures (HDRS, response, and remission) with 1) the rare (i.e., MAF<5%) variant count and its interaction with visit (i.e., time) and 2) the 9 prioritized frequent (i.e., MAF≥5% and RegulomeDB rank of 1 or 2) SNPs analyzed and their interactions with visit. Significant (i.e., *p* < 0.05) associations are highlighted in dark gray and bolded. Significance of fixed effects was assessed with the Satterthwaite method in mixed-effects models of the HDRS score and with the Wald chi-square test in mixed-effects models of response and remission. All models were adjusted for age, sex, antidepressant class, and any sociodemographic characteristics that significantly (bold text and *: *p* < 0.05) differed between genotypes (see Supplementary Table S2).

	HDRS						Response			Remission		
	SS	MS	df (num)	df (den)	*F*	*p*	χ^2^	df	*p*	χ^2^	df	*p*
**MAF<5% count**												
MAF<5% count	804.264	804.264	1	438.056	26.87	<0.001*	5.81	1	0.016*	4.56	1	**0.033**
Visit:MAF<5% count	348.786	116.262	3	918.602	3.88	0.0090*	0.53	2	0.77	1.99	2	0.37
**Prioritized variants (MAF≥5% and RegulomeDB rank ≤ 2)**												
rs2279130	59.196	29.598	2	401.121	0.98	0.38	1.48	2	0.48	4.11	2	0.13
Visit:rs2279130	122.998	20.500	6	914.026	0.68	0.67	3.03	4	0.55	1.94	4	0.75
rs501372	5.277	2.638	2	371.393	0.09	0.92	1.16	2	0.56	1.50	2	0.47
Visit:rs501372	168.779	28.130	6	882.364	0.93	0.47	4.64	4	0.33	2.00	4	0.73
rs877711	29.797	14.898	2	411.229	0.50	0.61	1.17	2	0.56	1.10	2	0.58
Visit:rs877711	195.206	32.534	6	942.398	1.09	0.36	0.63	4	0.96	4.59	4	0.33
rs553664	147.286	73.643	2	399.038	2.51	0.08	0.49	2	0.78	3.76	2	0.15
Visit:rs553664	541.248	90.208	6	919.440	3.08	0.0055*	8.86	4	0.06	11.02	4	0.026*
rs536852	213.512	106.756	2	385.710	3.53	0.030*	0.85	2	0.66	3.85	2	0.15
Visit:rs536852	390.451	65.075	6	886.704	2.15	0.046*	7.11	4	0.13	12.84	4	0.012*
rs1676887	50.854	25.427	2	372.885	0.85	0.43	1.36	2	0.51	1.78	2	0.41
Visit:rs1676887	194.069	32.345	6	887.639	1.08	0.37	1.71	4	0.79	5.43	4	0.25
rs113636971	10.977	5.489	2	379.137	0.18	0.83	0.23	2	0.89	0.14	2	0.93
Visit:rs113636971	94.369	15.728	6	896.752	0.52	0.79	0.57	4	0.97	0.74	4	0.95
rs504683	64.300	32.150	2	373.296	1.05	0.35	2.47	2	0.29	2.47	2	0.29
Visit:rs504683	74.684	12.447	6	892.312	0.41	0.87	0.79	4	0.94	0.79	4	0.94
rs561923	17.422	8.711	2	366.270	0.28	0.75	1.03	2	0.60	0.31	2	0.86
Visit:rs561923	37.649	6.275	6	883.377	0.20	0.98	2.06	4	0.72	2.62	4	0.62

χ^**2**^, chi-square value; den, denominator; df, degrees of freedom; *F*, *F*-value; HDRS, 17-item Hamilton Rating Depression scale; MAF, minor allele frequency; MS, mean sum of squares; num, numerator; *p*: *p*-value; SS, sum of squares; “:” designates an interaction between model terms.

### 3.4 Clinical response according to prioritized variants

**FIGURE 5:**
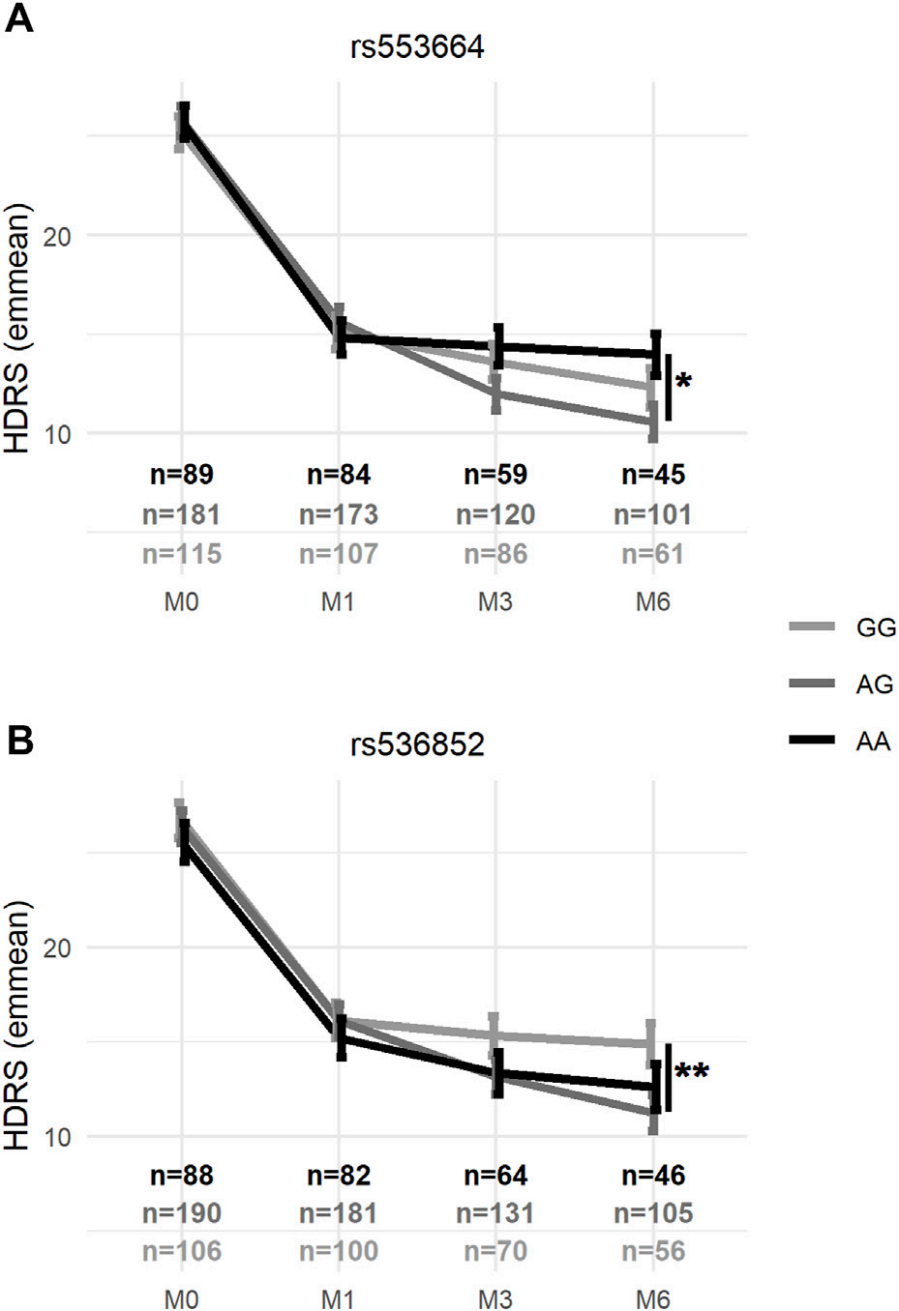
Average HDRS scores according to rs553664 and rs536852 genotypes over the course of antidepressant treatment. Average HDRS scores (*y*-axis) according to rs553664 **(A)** and rs536852 **(B)** genotypes (see legend) are shown across time (*x*-axis) after controlling for age, sex, antidepressant class, ethnicity, and significantly different demographic factors (see Supplementary Table S3). Error bars correspond to the standard error estimates from mixed-effects models. *: *p* < 0.0056 (0.05/9) **: *p* < 0.0011 (0.01/9). HDRS, 17-item Hamilton Depression Rating Scale; M0, baseline, prior to beginning antidepressant treatment; M1, 1 month after beginning antidepressant treatment; M3, 3 months after beginning antidepressant treatment; M6, 6 months after beginning antidepressant treatment.

**FIGURE 6:**
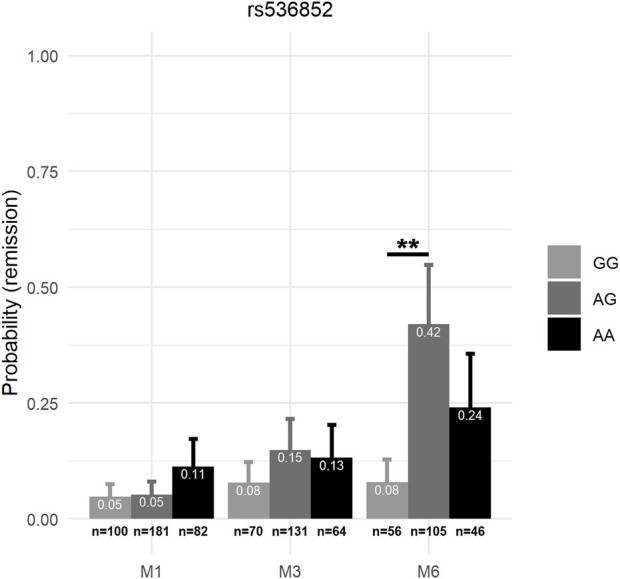
Remission rates according to rs536852 genotypes over the course of antidepressant treatment. The probability of remission (*y*-axis) according to rs536852 genotypes (see legend) are shown across time (*x*-axis) after controlling for age, sex, antidepressant class, ethnicity, and significantly different demographic factors (see Supplementary Table S2). Error bars correspond to the standard error estimates from mixed-effects models. **: *p* < 0.0011 (0.01/9). M1, 1 month after beginning antidepressant treatment; M3, 3 months after beginning antidepressant treatment; M6, 6 months after beginning antidepressant treatment.

Sociodemographic characteristics and significant differences between prioritized SNP genotypes are shown in Supplementary Table S2. Mixed-effects model associations of clinical measures with genotype and genotype × visit interactions for the 9 prioritized SNPs are summarized in Table 4. In mixed-effects models of the HDRS score controlling for age, sex, ATD class, ethnicity, and smoking status—and baseline HDRS scores for rs553664—the genotype × visit interaction was a significant explanatory factor for rs553664 (*F*_6,919_ = 3.08, *p* = 0.0055) and rs536852 (*F*_6,886_ = 2.15, *p* = 0.046) (see Table 4). In mixed-effects models of remission controlling for these same variables, the genotype × visit interaction was also significant for rs553664 [χ^2^ (df = 4, *n* = 388) = 11.02, *p* = 0.026] and rs536852 [χ^2^ (df = 4, *n* = 388) = 12.84, *p* = 0.012]. Following Bonferroni correction, a significantly higher HDRS score was observed in rs553664 AA homozygotes (13.98 ± 1.06) compared to AG heterozygotes (10.59 ± 0.86) at M6 (coefficient = 3.39, 95%CI [1.29–5.49], *p* = 0.014) (see Figure 5A) and in rs536852 GG homozygotes (14.88 ± 1.10) compared to AG heterozygotes (11.26 ± 0.95) at M6 (coefficient = 3.62, 95%CI [1.54–5.71], *p* = 0.0061) (see Figure 5B). A significantly lower remitter rate was also observed in rs536852 GG homozygotes (8%, *n* = 56) compared to AG heterozygotes (42%, *n* = 105) at M6 (OR = 0.12, 95%CI [0.39–0.37], *p* = 0.0018) (see Figure 6). No significant differences in response rates, or in remitter rates between rs553664 genotypes, were observed.

### 3.5 Functional analysis of ARRB1 polymorphisms

Annotations from RegulomeDB (v2.0.3) and HaploReg (v4.1) for rs553664 and rs536852—associated with clinical measures—and any variants in LD with them are shown in Table 5. rs553664 was observed to be in LD with rs506233 and an intronic deletion, rs35731045. rs536852 was not observed to be in LD with any variants within ±500,000 base pairs of its genomic position in non-Finnish European populations. It was most closely linked to rs553664 and rs506233, though these associations (*r* ^2^ = 0.67 and *r* ^2^ = 0.68, respectively) were weaker than in our sample (see Supplementary Figures S1, S2). rs553664, rs506233, and rs35731045 were annotated as binding FOS, NFIC, USF1, and IKZF1. rs536852 was annotated as binding POLR2A, FOS, and JUN. Altered TF binding motifs included the CCCTC-binding factor (CTCF). Each variant had at least one annotation with histone marks/chromatin state (see Table 5).

**TABLE 5:** HaploReg and RegulomeDB annotations. For rs553664 and rs536852, the rs#’s in linkage disequilibrium (i.e., *r* ^2^ > 80) with the given SNP, the associated histone marks/chromatin state in brain tissues, the bound proteins, and known and discovered altered motifs, are given.

rs #	rs #’s in LD (*r* ^2^ > 0.8)^a^	Histone marks^a^/chromatin state^b^ (brain)	Bound proteins	Altered TF motifs
rs553664	rs506233, rs35731045	Strong transcription^b^	NFYA^a^, USF1^a^, USF1^b^, USF2^b^, RAD51^b^, NFYC^b^, FOS^b^, EMSY^b^, CBFA2T2^b^, ZNF316^b^	Brachyury_1^a^, HP1-site-factor^a^, IRX3^b^, TBXT^b^
rs506233	rs553664, rs35731045	Enhancer^a^, promoter^a^, enhancers^b^, strong transcription^b^	NFIC^b^	Myc_known8^a^
rs35731045	rs553664, rs506233	Strong transcription^b^	IKZF1^b^	ERalpha-a_disc1^a^, ERalpha-a_disc2^a^, ERalpha-a_known1^a^, ERalpha-a_known4^a^, Esr2, RAR^a^, RXRA_disc1^a^, RXRA_known5^a^, SF1^a^, STAT_disc3^a^
rs536852		Enhancer^a^, promoter^a^, enhancers^b^, strong transcription^b^	CFOS^a^, CJUN^a^, FOS^b^, JUN^b^, POLR2A^b^	CTCF_disc9^a^, Irf_disc4^a^, Pax-4_3^a^, SP1_known2^a^, TATA_disc4^a^, TR4_disc3^a^, PAX4^b^

^a^ Annotation from HaploReg v4.1.

^b^ Annotation from RegulomeDB, v2.0.3. LD, linkage disequilibrium; TF, transcription factor.

## 4 Discussion

The present study is an ancillary investigation of the METADAP cohort that aimed to analyze the association between genetic variants of *ARRB1* and response following ATD treatment. Overall, 758 genetic variants passed quality control measures and were further filtered based on allelic frequency and their functional annotations as likely to affect TF binding. Analyses were carried out to assess the association of clinical response following ATD treatment with 1) the cumulative effect of rare (i.e., MAF<5%) variants and 2) frequent (i.e., MAF≥5%) variants with likely functional consequences. Twelve SNPs were prioritized, of which three pairs were observed to be in LD. Thus, 9 SNPs (3 proxies and 6 individual) were analyzed.

The variant set analysis using *SMMAT* (Chen et al., 2019) and leveraging the SKAT-O framework (Lee et al., 2012), suggests that the accumulation of rare *ARRB1* variants is associated with HDRS score changes and remission rates over the course of ATD treatment, as the burden test results were significant. As such, increased variant accumulation in *ARRB1* may have an impact on clinical response following ATD treatment. Furthermore, as reflected by the significant finding in the burden test, but not the SKAT test, a large proportion of these variants likely have causal effects on ATD response that, overall, act in the same direction (Lee et al., 2012). This was further highlighted by the significant association of greater rare variant counts with higher HDRS scores—notably at M1 and M3—and overall lower response and remission rates during treatment. Of note, certain rare variants in our cohort, including those in LD with the rs12274033 polymorphism that was previously associated with remission rates following mirtazapine treatment in a Korean population (Chang et al., 2015), may contribute to the observed associations with HDRS score changes and remission rates.

The 9 prioritized SNPs we analyzed have not been previously examined. They were prioritized for analysis from over 900 variants identified from HTS data spanning the *ARRB1* gene, including exons, introns, and 5′- and 3′-untranslated regions. Given that *ARRB1* mRNA and β-arrestin 1 protein levels have been previously associated with depression and ATD treatment (Avissar et al., 2004; Matuzany-Ruban et al., 2005; Golan et al., 2013), variants annotated as likely to lie in a functional element—and thus be more likely to impact *ARRB1* expression or β-arrestin function—were prioritized using RegulomeDB (Boyle et al., 2012). RegulomeDB has previously been used to select and study genetic variants in various disease backgrounds (Lee et al., 2015; Hong et al., 2018; Huo et al., 2019). Specifically, variants with a category ranking of 1 or 2, corresponding to variants likely to directly affect TF binding, were prioritized for further analysis. To our knowledge, this is the first study to leverage functional annotations to prioritize variants for analysis in the context of MDD and its treatment with ATDs.

Protein binding annotations from HaploReg and RegulomeDB, utilizing ChIP-seq data from ENCODE, suggest that rs553664 (and its proxies, including rs506233) and rs536852, associated with the HDRS score and remission in METADAP, lie within functional elements that bind various proteins including USF1, NFIC, FOS, JUN, and POLR2A. Genetic risk factors that overlap with TF-binding sites for USF1, NFIC, and POLR2A have been identified in relation to schizophrenia and MDD (Huo et al., 2019; Li et al., 2020).

FOS and JUN can dimerize to form a member of the Activator Protein-1 (AP-1) TF family, a leucine-zipper TF shown to regulate proliferation, differentiation, and apoptosis (Garces de los Fayos Alonso et al., 2018). Both FOS and JUN are expressed in the brain where they have context-dependent influences on neurodegeneration and/or neuroprotection (Herdegen and Waetzig, 2001). Recently, AP-1 was observed to have a role in fluoxetine response in a murine model of heightened anxiety (Chottekalapanda et al., 2020), and was also found to be activated by other factors such as BDNF, which is itself a factor influencing the response to different ATD treatments (Colle et al., 2015). It is known that ATDs can modify the activation of different genes through their modulation of serotonergic pathways (Lesch and Heils, 2000). Indeed, several TFs have been associated with ATD response or have been shown to be altered in depression (Pittenger and Duman, 2008; Sasayama et al., 2013). Moreover, the beta-arrestins act as scaffolds for kinases, including c-Jun N-terminal kinases of the JNK axis, which are implicated in neuronal plasticity, memory, and neuronal maturation (Herdegen and Waetzig, 2001; Shukla et al., 2011). As such, the potential impact rs553664 and/or rs536852 may have on AP-1 and/or the binding of other TFs could alter β-arrestin 1 expression and, consequently, any downstream functions of β-arrestin 1, including the transmission of receptor-mediated signals during ATD treatment or the scaffolding of proteins.

A putative binding motif for CTCF was also associated with rs536852. CTCF is a conserved zinc-finger TF with important roles in transcriptional regulation and 3D genome organization (Li et al., 2020). In a study of risk alleles for schizophrenia, SNPs lying in CTCF binding sites were enriched among those analyzed (Huo et al., 2019). Furthermore, in a study of MDD genetic risk factors, 11 of the 34 TF binding-disrupting SNPs analyzed were specific to CTCF binding sites, suggesting that disruption of CTCF binding may be a shared mechanism among genetic risk variants of MDD (Li et al., 2020). ATD-induced transcriptional alterations in the brain may also be influenced by CTCF (Piechota et al., 2015). Of course, whether CTCF binding is disrupted and, if so, the biological and clinical consequences of this, should be verified and further investigated.

Our findings demonstrate an association between genetic variants of *ARRB1* and clinical outcomes of response following ATD treatment. These findings suggest that these genetic factors might influence various mechanisms involved in the response to ATD therapy. Given that biased agonists can preferentially activate or bypass β-arrestin 1-mediated processes (Violin and Lefkowitz, 2007; Cottingham et al., 2011; Shukla et al., 2011; Bond et al., 2019), patients carrying either an excess of rare *ARRB1* variants or the rs553664 and rs536852 polymorphisms may benefit from the use of ATD treatments that 1) favor G-protein-mediated pathways and 2) circumvent β-arrestin-mediated pathways and the desensitization of GPCRs. Alternatively, these patients could also benefit from the addition of other therapeutical strategies, such as psychotherapies (i.e., talk therapies) or brain stimulation therapies, including ECT and repetitive transcranial magnetic stimulation.

Our study has several limitations. First, the proportion of missing data was relatively high, though the proportion at 6 months (46.1%) was comparable to the proportions in the STAR*D cohort at 9 (43%) and 12 weeks (61%) (Trivedi et al., 2006; Muthén et al., 2011). Additionally, mixed-effects models are a robust method to control for the bias imposed by these missing data (Mallinckrodt et al., 2003). Second, hard filters were used to filter out poor-quality variants, which could have removed variants near the cutoff threshold. Importantly, these variants could have had significant associations with clinical measures. Third, it should be noted that the functions of β-arrestin 1 and 2 often overlap. Thus, any impact that variants of *ARRB1* may have on β-arrestin 1 expression or function may be compensated for by β-arrestin 2, at least within the cytoplasm (Shukla et al., 2011). The strengths of this study include its prospective and naturalistic design, which allows for the analysis of treatment response across a 6-month period and better reflects “real-world” clinical practice. Additionally, this study leveraged functional annotations to select and prioritize variants with likely functional consequences on *ARRB1* expression for analysis since reduced β-arrestin 1 levels have been previously associated with depression severity and the response to ATD treatment (Avissar et al., 2004; Matuzany-Ruban et al., 2005; Golan et al., 2013). Finally, to date, this is the largest cohort analysis of *ARRB1* variants in relation to response following ATD treatment in patients with MDD. The findings from this exploratory analysis should be replicated in independent cohorts of depressed individuals undergoing ATD treatment.

To conclude, *ARRB1* genetic variants were associated with clinical measures of response following ATD treatment in a cohort of ATD-treated depressed patients. Specifically, we observed associations between clinical measures and 1) rare variant accumulation and 2) two frequent variants with probable functional consequences, rs553664 and rs536852. These findings suggest that variants of *ARRB1*, and perhaps especially those within genomic regulatory regions, may contribute to clinical response following ATD treatment. Functional analyses would help confirm the potential effect(s) that these polymorphisms have on clinical improvement following antidepressant treatment. Further study in larger cohorts would also help corroborate the findings of our exploratory analysis.

## Data Availability

The data analyzed in this study is subject to the following licenses/restrictions: data are under the protection of health data regulation set by the French National Commission on Informatics and Liberty (Commission Nationale de l’Informatique et des Libertés, CNIL), in line with European regulations and the Data Protection Act, and the Comité de protection des personnes (CPP, equivalent to the Research Ethics Committee). The data can be available upon reasonable request to the principal investigator of the study (emmanuelle.corruble@aphp.fr). The French law forbids us to provide free access to METADAP data. Please feel free to contact us should you have any additional questions.
