# Supplementary material for: The association of ARRB1 polymorphisms with response to antidepressant treatment in depressed patients
Source: Front Pharmacol. 2022 Oct 26;13:974570. doi: 10.3389/fphar.2022.974570 (PMC9644891; doi:10.3389/fphar.2022.974570)
Supplement: Supplementary file 1 [file DataSheet1.docx]

## Supplementary Figure 1: LD plot for the 12 prioritized frequent SNPs

Shown are the LD associations between each of the 12 prioritized SNPs. Haplotype blocks are defined by an *r*^2^>0.80 (values shown in each square). **LD**: linkage disequilibrium


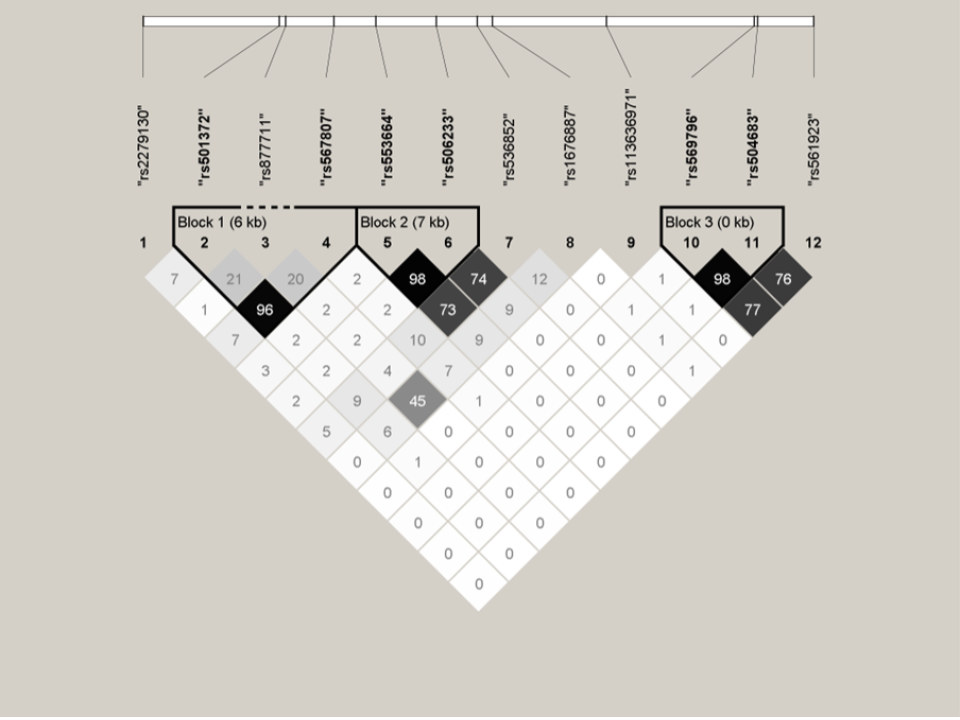


## Supplementary Figure 2: LD analysis of rs536852 using the 1000 Genomes Project

*r*^2^ associations relative to rs536822 (y-axis) in non-Finnish European populations of the 1000 Genomes Project between rs536852, rs553664, and rs506233, and linked variants, plotted according to their hg19 genomic position (x-axis). **LD**: linkage disequilibrium


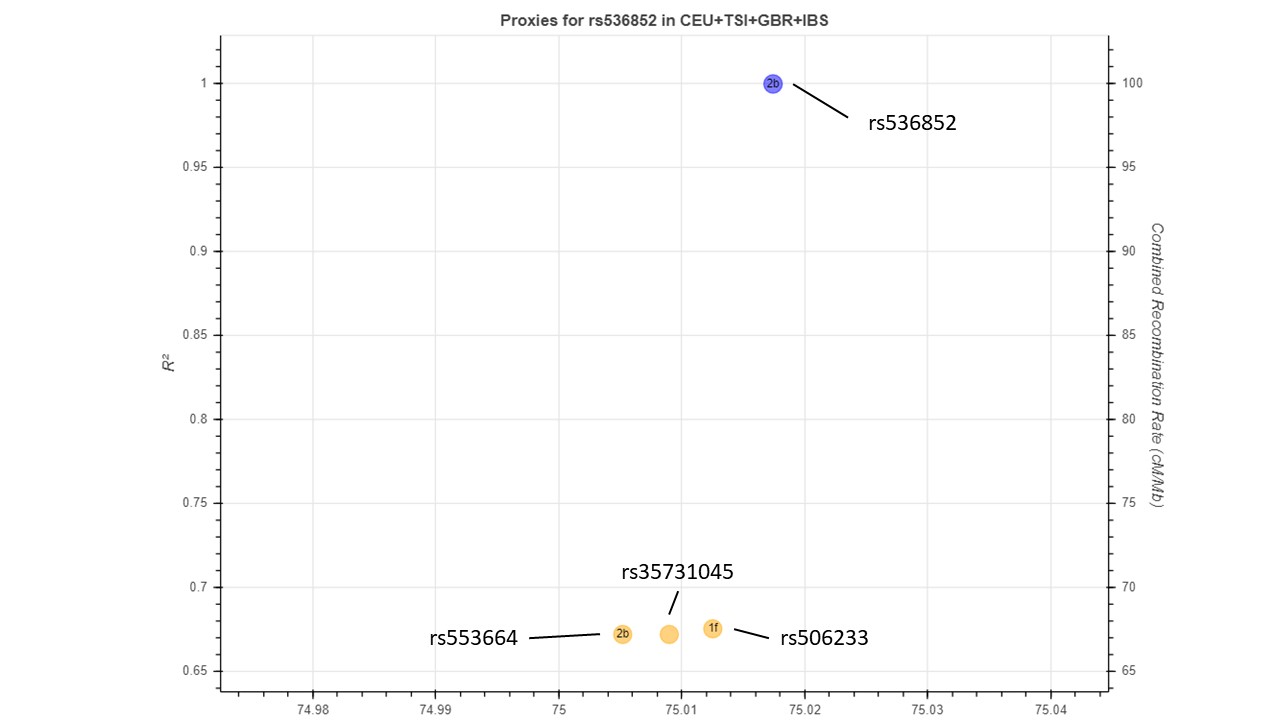


**Supplementary Table 1: *ARRB1* genetic variants detected from HTS**

For each *ARRB1* variant detected from HTS we report the chromosome, rs#, genomic position (hg19), reference allele, alternative allele, number of variant calls (# calls), the call rate, the number of alternative allele calls (AC), the total number of alleles (AN), the number of alternative allele homozygotes (# HOM), the allele frequency of the alternative allele (ALT AF), the minor allele, the MAF, and the RegulomeDB rank, if applicable. Dark green rows correspond to frequent (i.e., MAF≥5%) variants passing QC (i.e., call rate ≥95%) with a RegulomeDB rank of 1 or 2 (i.e., linked to expression of a gene target (rank 1) and likely to directly affect TF binding (ranks 1 and 2)). Light green rows correspond to frequent variants passing QC with a RegulomeDB rank >2. Light blue rows correspond to rare (i.e., MAF<5%) variants passing QC. Red rows correspond to variants not passing QC (i.e., call rate <95%). **HTS**: high-throughput sequencing; **MAF**: minor allele frequency; **QC**: quality control

| **chromosome** | **rs#** | **position (hg19)** | **reference** | **alternative** | **# calls** | **call rate** | **AC** | **AN** | **# HOM** | **ALT AF** | **minor** | **MAF** | **ranking** |
| --- | --- | --- | --- | --- | --- | --- | --- | --- | --- | --- | --- | --- | --- |
| chr11 | rs555031 | 75028607 | C | T | 284 | 0.9683 | 365 | 758 | 90 | 0.4815 | T | 0.4815 | 5 |
| chr11 | rs536852 | 75017436 | A | G | 300 | 0.9867 | 402 | 768 | 106 | 0.5234 | A | 0.4766 | 2b |
| chr11 | rs553664 | 75005209 | G | A | 273 | 0.989 | 359 | 770 | 89 | 0.4662 | A | 0.4662 | 2b |
| chr11 | rs7929974 | 75029167 | C | T | 281 | 0.879 | 330 | 708 | 83 | 0.4661 | T | 0.4661 |  |
| chr11 | rs506233 | 75012535 | A | G | 272 | 0.9559 | 344 | 752 | 84 | 0.4574 | G | 0.4574 | 1f |
| chr11 | rs616191 | 75044798 | T | C | 270 | 0.9815 | 341 | 766 | 76 | 0.4452 | C | 0.4452 | 3a |
| chr11 | rs2510894 | 75062178 | T | G | 313 | 0.984 | 428 | 766 | 120 | 0.5587 | T | 0.4413 | 4 |
| chr11 | rs35137614 | 75046634 | C | CA | 349 | 0.2923 | 122 | 282 | 20 | 0.4326 | CA | 0.4326 |  |
| chr11 | rs35731045 | 75008996 | GT | G | 272 | 0.7316 | 271 | 630 | 72 | 0.4302 | G | 0.4302 |  |
| chr11 | rs527106 | 75054436 | G | C | 314 | 0.9904 | 439 | 770 | 128 | 0.5701 | G | 0.4299 | 3a |
| chr11 | rs552454 | 75022481 | G | A | 260 | 0.9885 | 325 | 770 | 68 | 0.4221 | A | 0.4221 | 4 |
| chr11 | rs576014 | 75023441 | A | G | 262 | 0.9847 | 324 | 768 | 66 | 0.4219 | G | 0.4219 | 4 |
| chr11 | rs520563 | 75022058 | T | C | 260 | 0.9846 | 323 | 768 | 67 | 0.4206 | C | 0.4206 | 5 |
| chr11 | rs505443 | 75035994 | G | A | 329 | 0.8176 | 385 | 656 | 116 | 0.5869 | G | 0.4131 |  |
| chr11 | rs61237959 | 75014043 | C | CATT | 273 | 0.663 | 242 | 592 | 61 | 0.4088 | CATT | 0.4088 |  |
| chr11 | rs518232 | 75023968 | G | C | 329 | 0.9909 | 464 | 770 | 138 | 0.6026 | G | 0.3974 | 5 |
| chr11 | rs472112 | 75021501 | C | T | 328 | 0.9878 | 463 | 768 | 139 | 0.6029 | C | 0.3971 | 3a |
| chr11 | rs634287 | 74994694 | C | A | 334 | 0.9162 | 435 | 720 | 129 | 0.6042 | C | 0.3958 |  |
| chr11 | rs501372 | 74993593 | A | C | 334 | 0.982 | 462 | 764 | 134 | 0.6047 | A | 0.3953 | 2b |
| chr11 | rs631651 | 74994077 | C | G | 334 | 0.988 | 465 | 768 | 135 | 0.6055 | C | 0.3945 | 5 |
| chr11 | rs546812 | 75023093 | A | G | 328 | 0.9695 | 458 | 756 | 140 | 0.6058 | A | 0.3942 | 4 |
| chr11 | rs577673 | 74999933 | A | C | 335 | 0.9642 | 457 | 752 | 134 | 0.6077 | A | 0.3923 | 4 |
| chr11 | rs564011 | 75036116 | G | A | 329 | 0.9483 | 451 | 742 | 139 | 0.6078 | G | 0.3922 |  |
| chr11 | rs647630 | 74995350 | C | T | 335 | 0.9821 | 465 | 764 | 136 | 0.6086 | C | 0.3914 | 5 |
| chr11 | rs494146 | 75020596 | G | A | 247 | 0.9879 | 300 | 770 | 56 | 0.3896 | A | 0.3896 | 5 |
| chr11 | rs11284367 | 75045973 | GT | G | 354 | 0.548 | 282 | 456 | 88 | 0.6184 | GT | 0.3816 |  |
| chr11 | rs506448 | 75036115 | T | C | 331 | 0.9456 | 458 | 740 | 145 | 0.6189 | T | 0.3811 |  |
| chr11 | rs667791 | 74999428 | G | A | 344 | 0.9884 | 477 | 768 | 137 | 0.6211 | G | 0.3789 | 5 |
| chr11 | rs567807 | 75000189 | G | A | 344 | 0.9855 | 477 | 766 | 138 | 0.6227 | G | 0.3773 | 2b |
| chr11 | rs528833 | 74981039 | G | A | 239 | 0.9707 | 286 | 762 | 54 | 0.3753 | A | 0.3753 | 5 |
| chr11 | rs508435 | 75024816 | T | G | 337 | 0.9881 | 482 | 768 | 149 | 0.6276 | T | 0.3724 | 4 |
| chr11 | rs529513 | 75018232 | G | A | 340 | 0.9706 | 478 | 756 | 148 | 0.6323 | G | 0.3677 | 4 |
| chr11 | rs476364 | 75045900 | C | G | 338 | 0.9882 | 495 | 768 | 161 | 0.6445 | C | 0.3555 | 4 |
| chr11 | rs747221 | 75026331 | T | C | 337 | 0.9881 | 497 | 768 | 164 | 0.6471 | T | 0.3529 | 4 |
| chr11 | rs7127461 | 75032518 | G | C | 220 | 0.9773 | 269 | 766 | 54 | 0.3512 | C | 0.3512 | 5 |
| chr11 | rs61895989 | 75041337 | A | C | 342 | 0.9854 | 498 | 766 | 161 | 0.6501 | A | 0.3499 | 4 |
| chr11 | rs657561 | 75020379 | T | C | 344 | 0.9651 | 489 | 752 | 157 | 0.6503 | T | 0.3497 | 5 |
| chr11 | rs144522068 | 75041336 | C | CT | 342 | 0.9737 | 494 | 758 | 161 | 0.6517 | C | 0.3483 | 4 |
| chr11 | rs533085 | 75036757 | G | A | 343 | 0.9825 | 498 | 764 | 161 | 0.6518 | G | 0.3482 | 4 |
| chr11 | rs534889 | 75036953 | C | G | 343 | 0.9854 | 500 | 766 | 162 | 0.6527 | C | 0.3473 | 3a |
| chr11 | rs643523 | 75041207 | C | T | 342 | 0.9942 | 510 | 772 | 170 | 0.6606 | C | 0.3394 | 4 |
| chr11 | rs34053901 | 74988021 | G | GTCCCC | 221 | 0.8914 | 240 | 728 | 43 | 0.3297 | GTCCCC | 0.3297 |  |
| chr11 | rs578130 | 75003563 | G | A | 213 | 0.9812 | 249 | 768 | 40 | 0.3242 | A | 0.3242 | 5 |
| chr11 | rs55710203 | 75038643 | G | GGTT | 233 | 0.3948 | 160 | 494 | 68 | 0.3239 | GGTT | 0.3239 |  |
| chr11 | rs499332 | 75001451 | C | T | 356 | 0.9916 | 527 | 770 | 174 | 0.6844 | C | 0.3156 | 5 |
| chr11 | rs490528 | 75001493 | C | A | 357 | 0.9888 | 527 | 768 | 174 | 0.6862 | C | 0.3138 | 5 |
| chr11 | rs10899086 | 75026071 | G | A | 203 | 0.9754 | 239 | 766 | 41 | 0.3120 | A | 0.3120 | 5 |
| chr11 | rs745373 | 75002673 | G | C | 357 | 0.9804 | 525 | 762 | 175 | 0.6890 | G | 0.3110 | 4 |
| chr11 | rs553400 | 75028896 | A | G | 348 | 0.9828 | 546 | 764 | 204 | 0.7147 | A | 0.2853 | 3a |
| chr11 | rs504683 | 75051233 | A | G | 193 | 0.9896 | 218 | 772 | 27 | 0.2824 | G | 0.2824 | 2c |
| chr11 | rs569796 | 75050842 | G | C | 191 | 0.9895 | 216 | 772 | 27 | 0.2798 | C | 0.2798 | 1b |
| chr11 | rs2851459 | 75049054 | G | A | 189 | 0.9841 | 211 | 770 | 25 | 0.2740 | A | 0.2740 | 4 |
| chr11 | rs35479079 | 75043144 | CA | C | 358 | 0.7821 | 452 | 620 | 172 | 0.7290 | CA | 0.2710 |  |
| chr11 | rs2371216 | 75016833 | G | A | 178 | 0.9888 | 202 | 772 | 26 | 0.2617 | A | 0.2617 | 5 |
| chr11 | rs611908 | 75017087 | C | T | 179 | 1 | 203 | 776 | 24 | 0.2616 | T | 0.2616 | 5 |
| chr11 | rs7938192 | 75015367 | A | T | 178 | 0.9775 | 200 | 768 | 26 | 0.2604 | T | 0.2604 | 5 |
| chr11 | rs515590 | 75017666 | A | C | 181 | 0.9613 | 196 | 762 | 22 | 0.2572 | C | 0.2572 | 4 |
| chr11 | rs540123 | 75018144 | A | G | 181 | 0.9613 | 196 | 762 | 22 | 0.2572 | G | 0.2572 | 4 |
| chr11 | rs4945003 | 75005221 | G | T | 176 | 0.983 | 198 | 770 | 25 | 0.2571 | T | 0.2571 | 4 |
| chr11 | rs6592607 | 75009647 | C | T | 176 | 0.983 | 198 | 770 | 25 | 0.2571 | T | 0.2571 | 4 |
| chr11 | rs11607741 | 75026863 | A | C | 173 | 0.9827 | 197 | 770 | 27 | 0.2558 | C | 0.2558 | 5 |
| chr11 | rs73000501 | 75006407 | T | C | 176 | 0.9602 | 194 | 762 | 25 | 0.2546 | C | 0.2546 | 5 |
| chr11 | rs12797588 | 75059184 | C | T | 171 | 0.9825 | 196 | 770 | 28 | 0.2545 | T | 0.2545 | 4 |
| chr11 | rs678956 | 75046522 | A | G | 361 | 0.9612 | 558 | 748 | 211 | 0.7460 | A | 0.2540 | 5 |
| chr11 | rs547173 | 74998858 | A | G | 182 | 0.9615 | 193 | 762 | 18 | 0.2533 | G | 0.2533 | 5 |
| chr11 | rs4945005 | 75006877 | G | T | 175 | 0.9714 | 194 | 766 | 24 | 0.2533 | T | 0.2533 | 5 |
| chr11 | rs11236386 | 75009975 | A | G | 173 | 0.9827 | 195 | 770 | 25 | 0.2532 | G | 0.2532 | 6 |
| chr11 | rs11236385 | 75005955 | A | G | 175 | 0.96 | 192 | 762 | 24 | 0.2520 | G | 0.2520 | 6 |
| chr11 | rs531793 | 75026016 | C | T | 165 | 0.9576 | 191 | 762 | 33 | 0.2507 | T | 0.2507 | 5 |
| chr11 | rs665702 | 75057268 | T | C | 172 | 0.9767 | 192 | 768 | 24 | 0.2500 | C | 0.2500 | 4 |
| chr11 | rs566567 | 75059388 | A | G | 172 | 0.9767 | 191 | 768 | 23 | 0.2487 | G | 0.2487 | 4 |
| chr11 | rs2508622 | 75029106 | C | A | 361 | 0.9806 | 576 | 762 | 222 | 0.7559 | C | 0.2441 | 3a |
| chr11 | rs2508623 | 75029105 | G | A | 361 | 0.9806 | 576 | 762 | 222 | 0.7559 | G | 0.2441 | 3a |
| chr11 | rs562946 | 75052797 | A | C | 168 | 0.9821 | 185 | 770 | 20 | 0.2403 | C | 0.2403 | 4 |
| chr11 | rs561923 | 75058004 | C | A | 168 | 0.9643 | 182 | 764 | 20 | 0.2382 | A | 0.2382 | 2b |
| chr11 | rs526013 | 74993182 | A | G | 173 | 0.9595 | 180 | 762 | 14 | 0.2362 | G | 0.2362 | 5 |
| chr11 | rs55914269 | 75034713 | C | A | 155 | 0.9742 | 176 | 768 | 25 | 0.2292 | A | 0.2292 | 4 |
| chr11 | rs67362416 | 75034238 | G | A | 156 | 0.9615 | 172 | 764 | 22 | 0.2251 | A | 0.2251 | 4 |
| chr11 | rs746168 | 74992267 | A | C | 151 | 0.9735 | 172 | 768 | 25 | 0.2240 | C | 0.2240 | 4 |
| chr11 | rs10899084 | 75008570 | A | C | 150 | 0.9733 | 161 | 768 | 15 | 0.2096 | C | 0.2096 | 5 |
| chr11 | rs35518200 | 75003174 | A | ATATT | 364 | 0.467 | 310 | 388 | 140 | 0.7990 | A | 0.2010 |  |
| chr11 | rs55918409 | 75005661 | A | AT | 329 | 0.7143 | 470 | 588 | 235 | 0.7993 | A | 0.2007 |  |
| chr11 | rs480174 | 74995226 | G | A | 146 | 0.9384 | 148 | 758 | 11 | 0.1953 | A | 0.1953 | 5 |
| chr11 | rs616714 | 75044640 | G | T | 142 | 0.9648 | 148 | 766 | 11 | 0.1932 | T | 0.1932 | 4 |
| chr11 | rs489161 | 74999953 | A | G | 143 | 0.9441 | 145 | 760 | 10 | 0.1908 | G | 0.1908 | 4 |
| chr11 | rs542866 | 75044090 | C | A | 143 | 0.9301 | 144 | 756 | 11 | 0.1905 | A | 0.1905 | 7 |
| chr11 | rs578966 | 75003652 | A | G | 142 | 0.9507 | 143 | 762 | 8 | 0.1877 | G | 0.1877 | 5 |
| chr11 | rs687652 | 75022521 | T | C | 132 | 0.9924 | 140 | 774 | 9 | 0.1809 | C | 0.1809 | 4 |
| chr11 | rs750465 | 74990313 | G | C | 128 | 0.9609 | 138 | 766 | 15 | 0.1802 | C | 0.1802 | 4 |
| chr11 | rs477750 | 75009924 | A | G | 135 | 0.9481 | 137 | 762 | 9 | 0.1798 | G | 0.1798 | 6 |
| chr11 | rs76697641 | 75020511 | C | T | 128 | 0.9609 | 137 | 766 | 14 | 0.1789 | T | 0.1789 | 5 |
| chr11 | rs61232550 | 75008312 | GAAA | G | 147 | 0.8639 | 130 | 736 | 3 | 0.1766 | G | 0.1766 |  |
| chr11 | rs494647 | 75000549 | C | A | 135 | 0.8593 | 127 | 738 | 11 | 0.1721 | A | 0.1721 | 4 |
| chr11 | rs73492823 | 75047767 | C | A | 121 | 0.9835 | 127 | 772 | 8 | 0.1645 | A | 0.1645 | 4 |
| chr11 | rs146853679 | 74999660 | CTT | C | 316 | 0.1108 | 35 | 214 | 0 | 0.1636 | C | 0.1636 |  |
| chr11 | rs58388154 | 75020301 | C | T | 120 | 0.9083 | 121 | 754 | 12 | 0.1605 | T | 0.1605 | 5 |
| chr11 | rs56386203 | 75036745 | G | A | 106 | 0.9623 | 111 | 768 | 9 | 0.1445 | A | 0.1445 | 5 |
| chr11 | rs113289392 | 75026852 | G | GACCC | 108 | 0.9444 | 109 | 764 | 7 | 0.1427 | GACCC | 0.1427 |  |
| chr11 | rs34770388 | 75009286 | TA | T | 290 | 0.131 | 38 | 272 | 0 | 0.1397 | T | 0.1397 |  |
| chr11 | rs2276310 | 74982939 | C | T | 98 | 0.949 | 103 | 766 | 10 | 0.1345 | T | 0.1345 | 5 |
| chr11 | rs75220805 | 75020353 | C | T | 100 | 0.92 | 102 | 760 | 10 | 0.1342 | T | 0.1342 | 5 |
| chr11 | rs10531351 | 74984739 | ACCC | A | 378 | 0.9603 | 646 | 746 | 283 | 0.8660 | ACCC | 0.1340 | 4 |
| chr11 | rs67088202 | 74984744 | ATGT | A | 378 | 0.9603 | 646 | 746 | 283 | 0.8660 | ATGT | 0.1340 | 4 |
| chr11 | rs34867163 | 75062547 | A | G | 114 | 0.7982 | 96 | 730 | 5 | 0.1315 | G | 0.1315 | 4 |
| chr11 | rs3932962 | 74979280 | A | G | 99 | 0.9596 | 100 | 768 | 5 | 0.1302 | G | 0.1302 | 5 |
| chr11 | rs11602383 | 75028376 | G | C | 92 | 0.9239 | 92 | 762 | 7 | 0.1207 | C | 0.1207 | 5 |
| chr11 | rs12721489 | 74994679 | G | A | 91 | 0.9231 | 92 | 762 | 8 | 0.1207 | A | 0.1207 | 4 |
| chr11 | rs2236710 | 74994016 | C | A | 86 | 0.9651 | 91 | 770 | 8 | 0.1182 | A | 0.1182 | 5 |
| chr11 | rs877711 | 74994352 | G | A | 85 | 0.9765 | 91 | 772 | 8 | 0.1179 | A | 0.1179 | 2b |
| chr11 | rs1676888 | 75009892 | G | C | 378 | 0.9788 | 671 | 760 | 301 | 0.8829 | G | 0.1171 | 7 |
| chr11 | rs36123903 | 74982882 | G | A | 88 | 0.9545 | 88 | 768 | 4 | 0.1146 | A | 0.1146 | 5 |
| chr11 | rs67965584 | 75044211 | G | A | 87 | 0.954 | 88 | 768 | 5 | 0.1146 | A | 0.1146 | 4 |
| chr11 | rs1789685 | 74999322 | A | G | 380 | 0.9789 | 674 | 760 | 302 | 0.8868 | A | 0.1132 | 7 |
| chr11 | rs77712188 | 75026855 | C | A | 79 | 1 | 86 | 776 | 7 | 0.1108 | A | 0.1108 | 5 |
| chr11 | rs35242615 | 75029301 | G | A | 93 | 0.8065 | 81 | 740 | 6 | 0.1095 | A | 0.1095 | 4 |
| chr11 | rs899115 | 74995509 | C | G | 77 | 0.987 | 84 | 774 | 8 | 0.1085 | G | 0.1085 | 5 |
| chr11 | rs34044847 | 74997191 | C | T | 79 | 0.9494 | 83 | 768 | 8 | 0.1081 | T | 0.1081 | 5 |
| chr11 | rs138888342 | 74976042 | GA | G | 84 | 0.9167 | 82 | 762 | 5 | 0.1076 | G | 0.1076 |  |
| chr11 | rs17133921 | 75034986 | G | A | 79 | 0.9747 | 83 | 772 | 6 | 0.1075 | A | 0.1075 | 4 |
| chr11 | rs34903113 | 74997656 | A | G | 75 | 1 | 83 | 776 | 8 | 0.1070 | G | 0.1070 | 5 |
| chr11 | rs11604864 | 75055107 | C | T | 83 | 0.9277 | 81 | 764 | 4 | 0.1060 | T | 0.1060 | 5 |
| chr11 | rs2510658 | 75006292 | A | C | 380 | 0.8605 | 600 | 670 | 273 | 0.8955 | A | 0.1045 | 5 |
| chr11 | rs1676887 | 75019296 | A | G | 382 | 0.9895 | 688 | 768 | 310 | 0.8958 | A | 0.1042 | 1f |
| chr11 | rs512797 | 74996081 | G | A | 78 | 0.9615 | 80 | 770 | 5 | 0.1039 | A | 0.1039 | 5 |
| chr11 | rs12099326 | 75016233 | G | A | 166 | 0.2771 | 55 | 536 | 9 | 0.1026 | A | 0.1026 | 5 |
| chr11 | rs80306703 | 75031995 | T | C | 77 | 0.9481 | 78 | 768 | 5 | 0.1016 | C | 0.1016 | 4 |
| chr11 | rs685929 | 75028697 | T | C | 67 | 0.9851 | 76 | 774 | 10 | 0.0982 | C | 0.0982 | 3a |
| chr11 | rs4945006 | 75022559 | G | T | 71 | 0.9437 | 72 | 768 | 5 | 0.0938 | T | 0.0938 | 4 |
| chr11 | rs1783470 | 75021065 | T | C | 67 | 0.9851 | 70 | 774 | 4 | 0.0904 | C | 0.0904 | 5 |
| chr11 | NA | 75005650 | TA | T | 83 | 0.6867 | 63 | 724 | 6 | 0.0870 | T | 0.0870 |  |
| chr11 | rs58827950 | 75005646 | TAAA | T | 82 | 0.6951 | 63 | 726 | 6 | 0.0868 | T | 0.0868 |  |
| chr11 | rs56851527 | 75028760 | G | A | 65 | 0.9692 | 66 | 772 | 3 | 0.0855 | A | 0.0855 | 4 |
| chr11 | rs5792683 | 74987258 | G | GA | 386 | 0.6218 | 443 | 484 | 203 | 0.9153 | G | 0.0847 |  |
| chr11 | rs146453197 | 75040402 | C | T | 66 | 0.9242 | 64 | 766 | 3 | 0.0836 | T | 0.0836 | 2b |
| chr11 | rs1676890 | 74989276 | C | T | 385 | 0.974 | 693 | 756 | 318 | 0.9167 | C | 0.0833 | 5 |
| chr11 | rs113790622 | 75032038 | CA | C | 65 | 0.8923 | 62 | 762 | 4 | 0.0814 | C | 0.0814 |  |
| chr11 | rs11236389 | 75020708 | A | G | 60 | 0.9667 | 61 | 772 | 3 | 0.0790 | G | 0.0790 | 5 |
| chr11 | rs542645 | 75026392 | A | G | 58 | 0.9828 | 60 | 774 | 3 | 0.0775 | G | 0.0775 | 4 |
| chr11 | rs67906407 | 75023564 | G | A | 54 | 1 | 57 | 776 | 3 | 0.0735 | A | 0.0735 | 4 |
| chr11 | rs35439906 | 75062901 | TGC | T | 76 | 0.6447 | 53 | 722 | 4 | 0.0734 | T | 0.0734 |  |
| chr11 | rs2279130 | 74977185 | C | T | 54 | 0.9815 | 56 | 774 | 3 | 0.0724 | T | 0.0724 | 2b |
| chr11 | rs67373454 | 75023653 | C | G | 54 | 0.9815 | 56 | 774 | 3 | 0.0724 | G | 0.0724 | 4 |
| chr11 | rs140234723 | 75025417 | G | GC | 66 | 0.7273 | 53 | 740 | 5 | 0.0716 | GC | 0.0716 |  |
| chr11 | rs35786880 | 74980376 | C | T | 55 | 0.9273 | 54 | 768 | 3 | 0.0703 | T | 0.0703 | 5 |
| chr11 | rs2279129 | 74977067 | C | T | 54 | 0.963 | 54 | 772 | 2 | 0.0699 | T | 0.0699 | 4 |
| chr11 | rs2282600 | 74975662 | G | A | 50 | 1 | 53 | 776 | 3 | 0.0683 | A | 0.0683 | 4 |
| chr11 | rs71467873 | 75023291 | G | T | 54 | 0.9259 | 52 | 768 | 2 | 0.0677 | T | 0.0677 | 4 |
| chr11 | rs56117769 | 75023303 | G | A | 49 | 0.9796 | 52 | 774 | 4 | 0.0672 | A | 0.0672 | 4 |
| chr11 | rs4944078 | 75022695 | C | T | 49 | 0.9388 | 50 | 770 | 4 | 0.0649 | T | 0.0649 | 4 |
| chr11 | rs113636971 | 75033020 | A | G | 48 | 1 | 50 | 776 | 2 | 0.0644 | G | 0.0644 | 2b |
| chr11 | rs1621185 | 74996526 | T | C | 385 | 0.9974 | 725 | 774 | 341 | 0.9367 | T | 0.0633 | 3a |
| chr11 | rs1789681 | 74993789 | A | G | 385 | 0.9974 | 725 | 774 | 341 | 0.9367 | A | 0.0633 | 4 |
| chr11 | rs1676884 | 74995632 | A | C | 385 | 0.9948 | 724 | 772 | 341 | 0.9378 | A | 0.0622 | 5 |
| chr11 | NA | 75011263 | A | AAAAG | 84 | 0.4643 | 42 | 686 | 3 | 0.0612 | AAAAG | 0.0612 |  |
| chr11 | rs1789687 | 74997718 | T | C | 386 | 0.9922 | 723 | 770 | 340 | 0.9390 | T | 0.0610 | 5 |
| chr11 | rs142733324 | 74995946 | CAAAAT | C | 385 | 0.9714 | 708 | 754 | 334 | 0.9390 | CAAAAT | 0.0610 | 7 |
| chr11 | rs7938332 | 75024242 | C | T | 57 | 0.7544 | 45 | 748 | 2 | 0.0602 | T | 0.0602 | 5 |
| chr11 | rs1676886 | 74996955 | A | G | 385 | 0.987 | 720 | 766 | 340 | 0.9399 | A | 0.0601 | 5 |
| chr11 | rs1789688 | 74997562 | A | G | 385 | 0.9714 | 709 | 754 | 335 | 0.9403 | A | 0.0597 | 5 |
| chr11 | rs1320709 | 74995111 | A | G | 386 | 0.9845 | 719 | 764 | 339 | 0.9411 | A | 0.0589 | 5 |
| chr11 | rs59368208 | 75051936 | G | A | 48 | 0.9375 | 45 | 770 | 0 | 0.0584 | A | 0.0584 | 2b |
| chr11 | rs61265142 | 74971104 | G | A | 43 | 0.9535 | 44 | 772 | 3 | 0.0570 | A | 0.0570 | 4 |
| chr11 | rs12360620 | 75024372 | G | A | 48 | 0.875 | 43 | 764 | 1 | 0.0563 | A | 0.0563 | 3a |
| chr11 | rs35927438 | 75002801 | G | A | 44 | 0.9318 | 43 | 770 | 2 | 0.0558 | A | 0.0558 | 3a |
| chr11 | rs34559163 | 75002732 | A | G | 44 | 0.9318 | 42 | 770 | 1 | 0.0545 | G | 0.0545 | 4 |
| chr11 | rs146431282 | 75026051 | GC | G | 49 | 0.8367 | 41 | 760 | 0 | 0.0539 | G | 0.0539 |  |
| chr11 | rs1789689 | 74995674 | G | A | 386 | 0.9715 | 714 | 754 | 339 | 0.9469 | G | 0.0531 | 5 |
| chr11 | rs34772321 | 75015284 | C | T | 41 | 0.9268 | 40 | 770 | 2 | 0.0519 | T | 0.0519 | 5 |
| chr11 | rs11236388 | 75013792 | C | T | 38 | 1 | 40 | 776 | 2 | 0.0515 | T | 0.0515 | 5 |
| chr11 | rs72998602 | 74972833 | A | G | 37 | 1 | 39 | 776 | 2 | 0.0503 | G | 0.0503 | 4 |
| chr11 | rs17133858 | 74971557 | G | T | 38 | 0.9474 | 38 | 772 | 2 | 0.0492 | T | 0.0492 |  |
| chr11 | rs1789682 | 75013896 | G | A | 386 | 0.9896 | 731 | 768 | 349 | 0.9518 | G | 0.0482 |  |
| chr11 | rs1972287 | 75011263 | A | G | 76 | 0.3553 | 32 | 678 | 5 | 0.0472 | G | 0.0472 |  |
| chr11 | rs78757099 | 75059358 | C | T | 32 | 1 | 34 | 776 | 2 | 0.0438 | T | 0.0438 |  |
| chr11 | rs12721494 | 74979856 | C | T | 36 | 0.8611 | 33 | 766 | 2 | 0.0431 | T | 0.0431 |  |
| chr11 | rs148649394 | 75015481 | GA | G | 39 | 0.7692 | 32 | 758 | 2 | 0.0422 | G | 0.0422 |  |
| chr11 | rs34999509 | 75001034 | G | A | 31 | 1 | 32 | 776 | 1 | 0.0412 | A | 0.0412 |  |
| chr11 | rs35776328 | 74993485 | G | A | 29 | 0.9655 | 29 | 774 | 1 | 0.0375 | A | 0.0375 |  |
| chr11 | NA | 74972225 | CGT | C | 156 | 0.1026 | 16 | 496 | 0 | 0.0323 | C | 0.0323 |  |
| chr11 | rs200373325 | 75012602 | T | TA | 28 | 0.7857 | 22 | 764 | 0 | 0.0288 | TA | 0.0288 |  |
| chr11 | rs11236393 | 75028785 | G | A | 21 | 0.9048 | 22 | 772 | 3 | 0.0285 | A | 0.0285 |  |
| chr11 | rs117930709 | 75035511 | C | G | 21 | 1 | 21 | 776 | 0 | 0.0271 | G | 0.0271 |  |
| chr11 | rs35329661 | 74974990 | C | T | 21 | 1 | 21 | 776 | 0 | 0.0271 | T | 0.0271 |  |
| chr11 | rs75765618 | 75017857 | T | C | 20 | 1 | 21 | 776 | 1 | 0.0271 | C | 0.0271 |  |
| chr11 | rs10562128 | 74984393 | CTGGGG | C | 382 | 0.7408 | 563 | 578 | 280 | 0.9740 | CTGGGG | 0.0260 |  |
| chr11 | rs112296637 | 75036838 | C | T | 20 | 1 | 20 | 776 | 0 | 0.0258 | T | 0.0258 |  |
| chr11 | rs117944171 | 75038486 | C | T | 21 | 0.9048 | 19 | 772 | 0 | 0.0246 | T | 0.0246 |  |
| chr11 | rs139809706 | 74988155 | TC | T | 21 | 0.9048 | 19 | 772 | 0 | 0.0246 | T | 0.0246 |  |
| chr11 | rs117311695 | 75036357 | T | C | 19 | 1 | 19 | 776 | 0 | 0.0245 | C | 0.0245 |  |
| chr11 | rs113880482 | 75009817 | GAA | G | 19 | 0.8947 | 18 | 772 | 1 | 0.0233 | G | 0.0233 |  |
| chr11 | rs79040257 | 75009798 | C | A | 19 | 0.8947 | 18 | 772 | 1 | 0.0233 | A | 0.0233 |  |
| chr11 | rs117615697 | 75039324 | T | A | 18 | 1 | 18 | 776 | 0 | 0.0232 | A | 0.0232 |  |
| chr11 | rs7107762 | 75029474 | C | G | 25 | 0.6 | 17 | 756 | 2 | 0.0225 | G | 0.0225 |  |
| chr11 | rs138056781 | 75009966 | CATA | C | 19 | 0.8421 | 17 | 770 | 1 | 0.0221 | C | 0.0221 |  |
| chr11 | rs76522744 | 75040231 | A | C | 18 | 0.8889 | 17 | 772 | 1 | 0.0220 | C | 0.0220 |  |
| chr11 | rs149819755 | 75031514 | G | T | 18 | 0.9444 | 17 | 774 | 0 | 0.0220 | T | 0.0220 |  |
| chr11 | rs117147721 | 75049333 | G | T | 17 | 0.9412 | 16 | 774 | 0 | 0.0207 | T | 0.0207 |  |
| chr11 | rs147623751 | 75049331 | C | G | 17 | 0.9412 | 16 | 774 | 0 | 0.0207 | G | 0.0207 |  |
| chr11 | rs117857233 | 75051099 | C | T | 16 | 1 | 16 | 776 | 0 | 0.0206 | T | 0.0206 |  |
| chr11 | rs55708216 | 75017947 | T | A | 17 | 0.8235 | 15 | 770 | 1 | 0.0195 | A | 0.0195 |  |
| chr11 | rs56015415 | 75017533 | A | G | 16 | 0.875 | 15 | 772 | 1 | 0.0194 | G | 0.0194 |  |
| chr11 | rs76635068 | 75019383 | A | C | 16 | 0.875 | 15 | 772 | 1 | 0.0194 | C | 0.0194 |  |
| chr11 | rs145478334 | 75036818 | C | G | 16 | 0.9375 | 15 | 774 | 0 | 0.0194 | G | 0.0194 |  |
| chr11 | rs74528524 | 75024437 | C | T | 15 | 0.9333 | 15 | 774 | 1 | 0.0194 | T | 0.0194 |  |
| chr11 | rs75585826 | 75022870 | G | A | 14 | 0.9286 | 15 | 774 | 2 | 0.0194 | A | 0.0194 |  |
| chr11 | rs35382855 | 75062783 | G | A | 24 | 0.5833 | 14 | 756 | 0 | 0.0185 | A | 0.0185 |  |
| chr11 | rs58741836 | 75017721 | G | T | 15 | 0.8667 | 14 | 772 | 1 | 0.0181 | T | 0.0181 |  |
| chr11 | rs77612168 | 75022398 | A | T | 14 | 0.9286 | 14 | 774 | 1 | 0.0181 | T | 0.0181 |  |
| chr11 | rs139848901 | 75045543 | T | C | 14 | 1 | 14 | 776 | 0 | 0.0180 | C | 0.0180 |  |
| chr11 | rs111626932 | 75010964 | TG | T | 15 | 0.8 | 13 | 770 | 1 | 0.0169 | T | 0.0169 |  |
| chr11 | rs57430303 | 75017930 | GTCTCCCTAGACC | G | 17 | 0.6471 | 12 | 764 | 1 | 0.0157 | G | 0.0157 |  |
| chr11 | rs79912001 | 75020867 | A | G | 13 | 0.8462 | 12 | 772 | 1 | 0.0155 | G | 0.0155 |  |
| chr11 | rs116477105 | 74974036 | C | T | 12 | 1 | 12 | 776 | 0 | 0.0155 | T | 0.0155 |  |
| chr11 | rs141403740 | 75013588 | G | A | 12 | 1 | 12 | 776 | 0 | 0.0155 | A | 0.0155 |  |
| chr11 | rs58135266 | 75053740 | T | C | 12 | 1 | 12 | 776 | 0 | 0.0155 | C | 0.0155 |  |
| chr11 | rs73492805 | 75035749 | T | C | 12 | 1 | 12 | 776 | 0 | 0.0155 | C | 0.0155 |  |
| chr11 | rs669562 | 75005651 | A | T | 13 | 0.8462 | 11 | 772 | 0 | 0.0142 | T | 0.0142 |  |
| chr11 | rs34588870 | 75002535 | C | T | 11 | 1 | 11 | 776 | 0 | 0.0142 | T | 0.0142 |  |
| chr11 | rs35452852 | 75000054 | G | A | 11 | 1 | 11 | 776 | 0 | 0.0142 | A | 0.0142 |  |
| chr11 | rs35495581 | 75001150 | C | T | 11 | 1 | 11 | 776 | 0 | 0.0142 | T | 0.0142 |  |
| chr11 | rs35702714 | 74978077 | T | C | 11 | 1 | 11 | 776 | 0 | 0.0142 | C | 0.0142 |  |
| chr11 | NA | 74974784 | G | GA | 176 | 0.0341 | 6 | 436 | 0 | 0.0138 | GA | 0.0138 |  |
| chr11 | rs200780395 | 75024449 | TG | T | 13 | 0.7692 | 10 | 770 | 0 | 0.0130 | T | 0.0130 |  |
| chr11 | rs737410 | 75026082 | A | G | 12 | 0.8333 | 10 | 772 | 0 | 0.0130 | G | 0.0130 |  |
| chr11 | rs140877759 | 75001910 | A | T | 10 | 1 | 10 | 776 | 0 | 0.0129 | T | 0.0129 |  |
| chr11 | rs147638918 | 74972839 | C | T | 10 | 1 | 10 | 776 | 0 | 0.0129 | T | 0.0129 |  |
| chr11 | rs148007053 | 74995704 | T | C | 10 | 1 | 10 | 776 | 0 | 0.0129 | C | 0.0129 |  |
| chr11 | NA | 75005660 | T | A | 12 | 0.75 | 9 | 770 | 0 | 0.0117 | A | 0.0117 |  |
| chr11 | rs116841168 | 75031634 | G | A | 9 | 0.8889 | 9 | 774 | 1 | 0.0116 | A | 0.0116 |  |
| chr11 | rs35110913 | 74996546 | G | A | 10 | 0.9 | 9 | 774 | 0 | 0.0116 | A | 0.0116 |  |
| chr11 | rs117132831 | 74973107 | C | T | 9 | 1 | 9 | 776 | 0 | 0.0116 | T | 0.0116 |  |
| chr11 | rs148509849 | 75056580 | C | T | 9 | 1 | 9 | 776 | 0 | 0.0116 | T | 0.0116 |  |
| chr11 | NA | 74972225 | CGTGT | C | 115 | 0.0522 | 6 | 558 | 0 | 0.0108 | C | 0.0108 |  |
| chr11 | rs141824154 | 75032312 | C | T | 21 | 0.3333 | 8 | 748 | 1 | 0.0107 | T | 0.0107 |  |
| chr11 | NA | 75010281 | TA | T | 19 | 0.4211 | 8 | 754 | 0 | 0.0106 | T | 0.0106 |  |
| chr11 | rs73492803 | 75035730 | A | G | 9 | 0.8889 | 8 | 774 | 0 | 0.0103 | G | 0.0103 |  |
| chr11 | rs10557032 | 75015356 | ACT | A | 8 | 1 | 8 | 776 | 0 | 0.0103 | A | 0.0103 |  |
| chr11 | rs112272479 | 75057736 | T | C | 8 | 1 | 8 | 776 | 0 | 0.0103 | C | 0.0103 |  |
| chr11 | rs12577286 | 75058309 | C | A | 8 | 1 | 8 | 776 | 0 | 0.0103 | A | 0.0103 |  |
| chr11 | rs186782401 | 75043297 | G | A | 8 | 1 | 8 | 776 | 0 | 0.0103 | A | 0.0103 |  |
| chr11 | rs73492828 | 75058036 | C | T | 8 | 1 | 8 | 776 | 0 | 0.0103 | T | 0.0103 |  |
| chr11 | rs78308688 | 75035365 | C | G | 8 | 1 | 8 | 776 | 0 | 0.0103 | G | 0.0103 |  |
| chr11 | NA | 75056702 | G | A | 8 | 1 | 8 | 776 | 0 | 0.0103 | A | 0.0103 |  |
| chr11 | NA | 75043293 | T | A | 8 | 1 | 8 | 776 | 0 | 0.0103 | A | 0.0103 |  |
| chr11 | rs76688928 | 75016175 | T | G | 25 | 0.28 | 7 | 740 | 0 | 0.0095 | G | 0.0095 |  |
| chr11 | rs146812830 | 75013225 | CA | C | 12 | 0.5833 | 7 | 766 | 0 | 0.0091 | C | 0.0091 |  |
| chr11 | rs111531646 | 75052578 | G | C | 9 | 0.7778 | 7 | 772 | 0 | 0.0091 | C | 0.0091 |  |
| chr11 | rs79472038 | 75023720 | A | G | 8 | 0.875 | 7 | 774 | 0 | 0.0090 | G | 0.0090 |  |
| chr11 | rs114951214 | 75026303 | T | C | 7 | 1 | 7 | 776 | 0 | 0.0090 | C | 0.0090 |  |
| chr11 | rs117757400 | 75033092 | C | G | 7 | 1 | 7 | 776 | 0 | 0.0090 | G | 0.0090 |  |
| chr11 | rs148130209 | 75010773 | C | G | 7 | 1 | 7 | 776 | 0 | 0.0090 | G | 0.0090 |  |
| chr11 | rs35118888 | 75019788 | CA | C | 29 | 0.2069 | 6 | 730 | 0 | 0.0082 | C | 0.0082 |  |
| chr11 | NA | 75008937 | T | A | 19 | 0.3158 | 6 | 750 | 0 | 0.0080 | A | 0.0080 |  |
| chr11 | rs35807702 | 74997477 | A | AT | 13 | 0.4615 | 6 | 762 | 0 | 0.0079 | AT | 0.0079 |  |
| chr11 | rs34604653 | 74978255 | G | A | 8 | 0.75 | 6 | 772 | 0 | 0.0078 | A | 0.0078 |  |
| chr11 | rs145235225 | 75022358 | C | T | 7 | 0.8571 | 6 | 774 | 0 | 0.0078 | T | 0.0078 |  |
| chr11 | rs34914104 | 74993974 | G | A | 7 | 0.8571 | 6 | 774 | 0 | 0.0078 | A | 0.0078 |  |
| chr11 | NA | 75056680 | A | AT | 7 | 0.8571 | 6 | 774 | 0 | 0.0078 | AT | 0.0078 |  |
| chr11 | rs116633202 | 75053381 | C | T | 5 | 1 | 6 | 776 | 1 | 0.0077 | T | 0.0077 |  |
| chr11 | rs117593728 | 75016419 | C | G | 6 | 1 | 6 | 776 | 0 | 0.0077 | G | 0.0077 |  |
| chr11 | rs12360540 | 75057649 | A | G | 6 | 1 | 6 | 776 | 0 | 0.0077 | G | 0.0077 |  |
| chr11 | rs147397456 | 75017510 | A | T | 6 | 1 | 6 | 776 | 0 | 0.0077 | T | 0.0077 |  |
| chr11 | rs190508071 | 74988971 | C | T | 6 | 1 | 6 | 776 | 0 | 0.0077 | T | 0.0077 |  |
| chr11 | rs34251325 | 74983399 | C | T | 5 | 1 | 6 | 776 | 1 | 0.0077 | T | 0.0077 |  |
| chr11 | rs35666856 | 74982794 | A | G | 6 | 1 | 6 | 776 | 0 | 0.0077 | G | 0.0077 |  |
| chr11 | rs58320160 | 75004913 | G | A | 6 | 1 | 6 | 776 | 0 | 0.0077 | A | 0.0077 |  |
| chr11 | rs77043015 | 75032905 | T | C | 6 | 1 | 6 | 776 | 0 | 0.0077 | C | 0.0077 |  |
| chr11 | rs79059324 | 75026450 | A | G | 6 | 1 | 6 | 776 | 0 | 0.0077 | G | 0.0077 |  |
| chr11 | rs7952044 | 74987683 | C | T | 6 | 1 | 6 | 776 | 0 | 0.0077 | T | 0.0077 |  |
| chr11 | rs11361682 | 75027368 | CA | C | 69 | 0.0725 | 5 | 648 | 0 | 0.0077 | C | 0.0077 |  |
| chr11 | rs112776931 | 74981670 | CA | C | 252 | 0.0079 | 2 | 276 | 0 | 0.0072 | C | 0.0072 |  |
| chr11 | NA | 75008938 | T | A | 19 | 0.2632 | 5 | 748 | 0 | 0.0067 | A | 0.0067 |  |
| chr11 | rs34825019 | 74996289 | G | A | 7 | 0.7143 | 5 | 772 | 0 | 0.0065 | A | 0.0065 |  |
| chr11 | rs114167831 | 75017435 | C | T | 5 | 1 | 5 | 776 | 0 | 0.0064 | T | 0.0064 |  |
| chr11 | rs141218825 | 75022989 | G | A | 5 | 1 | 5 | 776 | 0 | 0.0064 | A | 0.0064 |  |
| chr11 | rs142666480 | 74989804 | G | A | 5 | 1 | 5 | 776 | 0 | 0.0064 | A | 0.0064 |  |
| chr11 | rs146293962 | 75029946 | C | A | 5 | 1 | 5 | 776 | 0 | 0.0064 | A | 0.0064 |  |
| chr11 | rs181825944 | 75017762 | A | G | 5 | 1 | 5 | 776 | 0 | 0.0064 | G | 0.0064 |  |
| chr11 | rs181873855 | 74972113 | T | G | 5 | 1 | 5 | 776 | 0 | 0.0064 | G | 0.0064 |  |
| chr11 | rs34879796 | 75000732 | C | T | 5 | 1 | 5 | 776 | 0 | 0.0064 | T | 0.0064 |  |
| chr11 | rs35272648 | 74980961 | G | A | 5 | 1 | 5 | 776 | 0 | 0.0064 | A | 0.0064 |  |
| chr11 | rs41429652 | 74971747 | C | T | 5 | 1 | 5 | 776 | 0 | 0.0064 | T | 0.0064 |  |
| chr11 | rs60527753 | 75032415 | A | T | 5 | 1 | 5 | 776 | 0 | 0.0064 | T | 0.0064 |  |
| chr11 | rs61116397 | 74971982 | T | C | 5 | 1 | 5 | 776 | 0 | 0.0064 | C | 0.0064 |  |
| chr11 | rs61133275 | 74971980 | C | CT | 5 | 1 | 5 | 776 | 0 | 0.0064 | CT | 0.0064 |  |
| chr11 | rs78604352 | 75019399 | C | T | 5 | 1 | 5 | 776 | 0 | 0.0064 | T | 0.0064 |  |
| chr11 | rs78839836 | 75016732 | C | T | 5 | 1 | 5 | 776 | 0 | 0.0064 | T | 0.0064 |  |
| chr11 | NA | 75016050 | T | TTTTC | 66 | 0.0455 | 4 | 650 | 1 | 0.0062 | TTTTC | 0.0062 |  |
| chr11 | rs201879386 | 75042362 | A | AT | 7 | 0.5714 | 4 | 770 | 0 | 0.0052 | AT | 0.0052 |  |
| chr11 | rs73490802 | 75035657 | G | A | 7 | 0.5714 | 4 | 770 | 0 | 0.0052 | A | 0.0052 |  |
| chr11 | NA | 75007252 | G | A | 7 | 0.5714 | 4 | 770 | 0 | 0.0052 | A | 0.0052 |  |
| chr11 | rs113466990 | 75028866 | G | A | 6 | 0.6667 | 4 | 772 | 0 | 0.0052 | A | 0.0052 |  |
| chr11 | NA | 75038643 | G | GGTTT | 4 | 0.5 | 4 | 772 | 2 | 0.0052 | GGTTT | 0.0052 |  |
| chr11 | rs116787983 | 75020323 | C | T | 4 | 0.75 | 4 | 774 | 1 | 0.0052 | T | 0.0052 |  |
| chr11 | rs374250082 | 75008944 | G | A | 5 | 0.8 | 4 | 774 | 0 | 0.0052 | A | 0.0052 |  |
| chr11 | rs56376940 | 75029262 | G | A | 5 | 0.8 | 4 | 774 | 0 | 0.0052 | A | 0.0052 |  |
| chr11 | rs74495262 | 74972713 | G | A | 5 | 0.8 | 4 | 774 | 0 | 0.0052 | A | 0.0052 |  |
| chr11 | NA | 75011254 | AAAAAAAAAAAAAG | A | 4 | 0.75 | 4 | 774 | 1 | 0.0052 | A | 0.0052 |  |
| chr11 | rs113276950 | 75017156 | G | A | 4 | 1 | 4 | 776 | 0 | 0.0052 | A | 0.0052 |  |
| chr11 | rs114712814 | 75041530 | C | T | 4 | 1 | 4 | 776 | 0 | 0.0052 | T | 0.0052 |  |
| chr11 | rs114803418 | 75034847 | C | T | 4 | 1 | 4 | 776 | 0 | 0.0052 | T | 0.0052 |  |
| chr11 | rs139036232 | 75050806 | G | A | 4 | 1 | 4 | 776 | 0 | 0.0052 | A | 0.0052 |  |
| chr11 | rs187975460 | 74975556 | G | A | 4 | 1 | 4 | 776 | 0 | 0.0052 | A | 0.0052 |  |
| chr11 | rs34804214 | 74994232 | A | G | 4 | 1 | 4 | 776 | 0 | 0.0052 | G | 0.0052 |  |
| chr11 | rs34824695 | 74974957 | G | A | 4 | 1 | 4 | 776 | 0 | 0.0052 | A | 0.0052 |  |
| chr11 | rs35948518 | 75060198 | C | T | 4 | 1 | 4 | 776 | 0 | 0.0052 | T | 0.0052 |  |
| chr11 | rs56056585 | 75054036 | A | G | 4 | 1 | 4 | 776 | 0 | 0.0052 | G | 0.0052 |  |
| chr11 | rs58723399 | 75053940 | C | T | 4 | 1 | 4 | 776 | 0 | 0.0052 | T | 0.0052 |  |
| chr11 | rs7102956 | 75028107 | C | T | 4 | 1 | 4 | 776 | 0 | 0.0052 | T | 0.0052 |  |
| chr11 | rs74667789 | 75054630 | G | A | 4 | 1 | 4 | 776 | 0 | 0.0052 | A | 0.0052 |  |
| chr11 | rs75123678 | 75058802 | C | T | 4 | 1 | 4 | 776 | 0 | 0.0052 | T | 0.0052 |  |
| chr11 | rs76469252 | 75058647 | C | T | 4 | 1 | 4 | 776 | 0 | 0.0052 | T | 0.0052 |  |
| chr11 | rs78023964 | 75058823 | C | T | 4 | 1 | 4 | 776 | 0 | 0.0052 | T | 0.0052 |  |
| chr11 | rs78759643 | 75052508 | C | T | 4 | 1 | 4 | 776 | 0 | 0.0052 | T | 0.0052 |  |
| chr11 | rs79279308 | 75028638 | C | T | 4 | 1 | 4 | 776 | 0 | 0.0052 | T | 0.0052 |  |
| chr11 | NA | 74980986 | AC | A | 4 | 1 | 4 | 776 | 0 | 0.0052 | A | 0.0052 |  |
| chr11 | NA | 75037985 | A | G | 4 | 1 | 4 | 776 | 0 | 0.0052 | G | 0.0052 |  |
| chr11 | NA | 75010491 | GA | G | 47 | 0.0638 | 3 | 688 | 0 | 0.0044 | G | 0.0044 |  |
| chr11 | rs60898398 | 75005642 | TAAA | T | 7 | 0.4286 | 3 | 768 | 0 | 0.0039 | T | 0.0039 |  |
| chr11 | rs111846212 | 75028603 | C | G | 5 | 0.6 | 3 | 772 | 0 | 0.0039 | G | 0.0039 |  |
| chr11 | rs138476145 | 75015572 | TA | T | 5 | 0.6 | 3 | 772 | 0 | 0.0039 | T | 0.0039 |  |
| chr11 | NA | 74981963 | CT | C | 5 | 0.6 | 3 | 772 | 0 | 0.0039 | C | 0.0039 |  |
| chr11 | NA | 75002908 | A | G | 5 | 0.6 | 3 | 772 | 0 | 0.0039 | G | 0.0039 |  |
| chr11 | rs145165645 | 75022869 | C | T | 4 | 0.75 | 3 | 774 | 0 | 0.0039 | T | 0.0039 |  |
| chr11 | rs186276983 | 74972407 | G | T | 4 | 0.75 | 3 | 774 | 0 | 0.0039 | T | 0.0039 |  |
| chr11 | rs188678299 | 75062798 | G | A | 4 | 0.75 | 3 | 774 | 0 | 0.0039 | A | 0.0039 |  |
| chr11 | rs77815997 | 75036044 | G | A | 4 | 0.75 | 3 | 774 | 0 | 0.0039 | A | 0.0039 |  |
| chr11 | rs80209113 | 75035973 | G | A | 4 | 0.75 | 3 | 774 | 0 | 0.0039 | A | 0.0039 |  |
| chr11 | rs114301297 | 75019172 | A | G | 3 | 1 | 3 | 776 | 0 | 0.0039 | G | 0.0039 |  |
| chr11 | rs115451430 | 75009132 | T | A | 3 | 1 | 3 | 776 | 0 | 0.0039 | A | 0.0039 |  |
| chr11 | rs116305392 | 75012878 | T | C | 3 | 1 | 3 | 776 | 0 | 0.0039 | C | 0.0039 |  |
| chr11 | rs116562787 | 75022589 | C | T | 3 | 1 | 3 | 776 | 0 | 0.0039 | T | 0.0039 |  |
| chr11 | rs116826601 | 75007525 | G | A | 3 | 1 | 3 | 776 | 0 | 0.0039 | A | 0.0039 |  |
| chr11 | rs11827808 | 75004494 | C | G | 3 | 1 | 3 | 776 | 0 | 0.0039 | G | 0.0039 |  |
| chr11 | rs12577043 | 74978088 | A | C | 2 | 1 | 3 | 776 | 1 | 0.0039 | C | 0.0039 |  |
| chr11 | rs12721493 | 74988048 | A | G | 3 | 1 | 3 | 776 | 0 | 0.0039 | G | 0.0039 |  |
| chr11 | rs138730282 | 75010389 | C | T | 3 | 1 | 3 | 776 | 0 | 0.0039 | T | 0.0039 |  |
| chr11 | rs143846053 | 75010111 | TG | T | 3 | 1 | 3 | 776 | 0 | 0.0039 | T | 0.0039 |  |
| chr11 | rs147847535 | 75009950 | A | G | 3 | 1 | 3 | 776 | 0 | 0.0039 | G | 0.0039 |  |
| chr11 | rs149950836 | 74983729 | A | AGAGGGGCT | 3 | 1 | 3 | 776 | 0 | 0.0039 | AGAGGGGCT | 0.0039 |  |
| chr11 | rs181310549 | 75027756 | G | A | 3 | 1 | 3 | 776 | 0 | 0.0039 | A | 0.0039 |  |
| chr11 | rs183308351 | 75062189 | G | A | 3 | 1 | 3 | 776 | 0 | 0.0039 | A | 0.0039 |  |
| chr11 | rs187332931 | 75052131 | A | G | 3 | 1 | 3 | 776 | 0 | 0.0039 | G | 0.0039 |  |
| chr11 | rs189080782 | 75053995 | T | C | 3 | 1 | 3 | 776 | 0 | 0.0039 | C | 0.0039 |  |
| chr11 | rs34598900 | 74975247 | G | A | 3 | 1 | 3 | 776 | 0 | 0.0039 | A | 0.0039 |  |
| chr11 | rs34643883 | 75000336 | T | G | 3 | 1 | 3 | 776 | 0 | 0.0039 | G | 0.0039 |  |
| chr11 | rs34674786 | 74996962 | C | T | 3 | 1 | 3 | 776 | 0 | 0.0039 | T | 0.0039 |  |
| chr11 | rs34899069 | 74990527 | C | T | 2 | 1 | 3 | 776 | 1 | 0.0039 | T | 0.0039 |  |
| chr11 | rs34945787 | 74997472 | C | T | 3 | 1 | 3 | 776 | 0 | 0.0039 | T | 0.0039 |  |
| chr11 | rs35891965 | 74983082 | G | A | 3 | 1 | 3 | 776 | 0 | 0.0039 | A | 0.0039 |  |
| chr11 | rs3893000 | 74974444 | C | T | 3 | 1 | 3 | 776 | 0 | 0.0039 | T | 0.0039 |  |
| chr11 | rs58880747 | 75039001 | T | C | 3 | 1 | 3 | 776 | 0 | 0.0039 | C | 0.0039 |  |
| chr11 | rs7102524 | 75010897 | C | A | 3 | 1 | 3 | 776 | 0 | 0.0039 | A | 0.0039 |  |
| chr11 | rs7112979 | 75011386 | G | T | 3 | 1 | 3 | 776 | 0 | 0.0039 | T | 0.0039 |  |
| chr11 | rs76407232 | 75049495 | T | G | 3 | 1 | 3 | 776 | 0 | 0.0039 | G | 0.0039 |  |
| chr11 | rs77079044 | 75048560 | C | G | 3 | 1 | 3 | 776 | 0 | 0.0039 | G | 0.0039 |  |
| chr11 | rs77165095 | 75040900 | T | C | 3 | 1 | 3 | 776 | 0 | 0.0039 | C | 0.0039 |  |
| chr11 | rs77304639 | 75006579 | T | C | 3 | 1 | 3 | 776 | 0 | 0.0039 | C | 0.0039 |  |
| chr11 | rs77380765 | 75010700 | A | G | 3 | 1 | 3 | 776 | 0 | 0.0039 | G | 0.0039 |  |
| chr11 | rs78103004 | 75010044 | G | A | 3 | 1 | 3 | 776 | 0 | 0.0039 | A | 0.0039 |  |
| chr11 | rs79577711 | 75048107 | G | A | 3 | 1 | 3 | 776 | 0 | 0.0039 | A | 0.0039 |  |
| chr11 | rs79781165 | 75050147 | C | T | 3 | 1 | 3 | 776 | 0 | 0.0039 | T | 0.0039 |  |
| chr11 | rs79950403 | 75062127 | C | T | 3 | 1 | 3 | 776 | 0 | 0.0039 | T | 0.0039 |  |
| chr11 | NA | 75042090 | C | T | 3 | 1 | 3 | 776 | 0 | 0.0039 | T | 0.0039 |  |
| chr11 | NA | 75043464 | T | C | 3 | 1 | 3 | 776 | 0 | 0.0039 | C | 0.0039 |  |
| chr11 | NA | 75045980 | T | G | 3 | 1 | 3 | 776 | 0 | 0.0039 | G | 0.0039 |  |
| chr11 | NA | 74980798 | TGAG | T | 3 | 1 | 3 | 776 | 0 | 0.0039 | T | 0.0039 |  |
| chr11 | NA | 74995466 | G | A | 3 | 1 | 3 | 776 | 0 | 0.0039 | A | 0.0039 |  |
| chr11 | NA | 75008272 | A | C | 3 | 1 | 3 | 776 | 0 | 0.0039 | C | 0.0039 |  |
| chr11 | NA | 75009868 | T | TATGTATGTATAG | 3 | 1 | 3 | 776 | 0 | 0.0039 | TATGTATGTATAG | 0.0039 |  |
| chr11 | NA | 75009959 | A | G | 3 | 1 | 3 | 776 | 0 | 0.0039 | G | 0.0039 |  |
| chr11 | NA | 75024457 | T | C | 3 | 1 | 3 | 776 | 0 | 0.0039 | C | 0.0039 |  |
| chr11 | NA | 75057530 | C | A | 3 | 1 | 3 | 776 | 0 | 0.0039 | A | 0.0039 |  |
| chr11 | NA | 74993432 | G | A | 3 | 1 | 3 | 776 | 0 | 0.0039 | A | 0.0039 |  |
| chr11 | NA | 74998822 | C | T | 3 | 1 | 3 | 776 | 0 | 0.0039 | T | 0.0039 |  |
| chr11 | NA | 75006305 | A | G | 3 | 1 | 3 | 776 | 0 | 0.0039 | G | 0.0039 |  |
| chr11 | NA | 75019764 | T | G | 3 | 1 | 3 | 776 | 0 | 0.0039 | G | 0.0039 |  |
| chr11 | NA | 74972225 | CGTGTGT | C | 49 | 0.0408 | 2 | 682 | 0 | 0.0029 | C | 0.0029 |  |
| chr11 | NA | 74974784 | G | GAA | 44 | 0.0455 | 2 | 692 | 0 | 0.0029 | GAA | 0.0029 |  |
| chr11 | NA | 75010491 | G | GA | 34 | 0.0588 | 2 | 712 | 0 | 0.0028 | GA | 0.0028 |  |
| chr11 | rs200753370 | 74974784 | GA | G | 33 | 0.0606 | 2 | 714 | 0 | 0.0028 | G | 0.0028 |  |
| chr11 | rs11236384 | 75005661 | A | T | 30 | 0.0667 | 2 | 720 | 0 | 0.0028 | T | 0.0028 |  |
| chr11 | rs55710203 | 75038643 | G | GGT | 23 | 0.0435 | 2 | 732 | 1 | 0.0027 | GGT | 0.0027 |  |
| chr11 | NA | 75008312 | GA | G | 21 | 0.0952 | 2 | 738 | 0 | 0.0027 | G | 0.0027 |  |
| chr11 | rs201536338 | 75038637 | G | GT | 12 | 0.1667 | 2 | 756 | 0 | 0.0026 | GT | 0.0026 |  |
| chr11 | rs28687723 | 75032316 | C | G | 7 | 0.2857 | 2 | 766 | 0 | 0.0026 | G | 0.0026 |  |
| chr11 | rs58827950 | 75005646 | TAAATA | T | 7 | 0.2857 | 2 | 766 | 0 | 0.0026 | T | 0.0026 |  |
| chr11 | rs113887473 | 74972117 | CCAATATCAGGGTCACGACCCATCCCCA | C | 5 | 0.4 | 2 | 770 | 0 | 0.0026 | C | 0.0026 |  |
| chr11 | rs112543856 | 75030637 | G | A | 4 | 0.5 | 2 | 772 | 0 | 0.0026 | A | 0.0026 |  |
| chr11 | rs200212797 | 75046000 | T | TA | 4 | 0.5 | 2 | 772 | 0 | 0.0026 | TA | 0.0026 |  |
| chr11 | rs112376035 | 75028456 | C | T | 3 | 0.6667 | 2 | 774 | 0 | 0.0026 | T | 0.0026 |  |
| chr11 | rs183297699 | 75018080 | A | G | 3 | 0.6667 | 2 | 774 | 0 | 0.0026 | G | 0.0026 |  |
| chr11 | rs4945005 | 75006877 | G | A | 3 | 0.6667 | 2 | 774 | 0 | 0.0026 | A | 0.0026 |  |
| chr11 | rs76004353 | 75007690 | C | A | 3 | 0.6667 | 2 | 774 | 0 | 0.0026 | A | 0.0026 |  |
| chr11 | rs922399 | 75040969 | A | T | 3 | 0.6667 | 2 | 774 | 0 | 0.0026 | T | 0.0026 |  |
| chr11 | rs1109759 | 75026000 | G | A | 2 | 1 | 2 | 776 | 0 | 0.0026 | A | 0.0026 |  |
| chr11 | rs115129654 | 75059241 | A | G | 2 | 1 | 2 | 776 | 0 | 0.0026 | G | 0.0026 |  |
| chr11 | rs115278549 | 75044595 | G | A | 2 | 1 | 2 | 776 | 0 | 0.0026 | A | 0.0026 |  |
| chr11 | rs115370400 | 75037584 | C | T | 2 | 1 | 2 | 776 | 0 | 0.0026 | T | 0.0026 |  |
| chr11 | rs115371375 | 75018468 | G | C | 2 | 1 | 2 | 776 | 0 | 0.0026 | C | 0.0026 |  |
| chr11 | rs115852953 | 75049362 | T | C | 2 | 1 | 2 | 776 | 0 | 0.0026 | C | 0.0026 |  |
| chr11 | rs115854157 | 75029852 | C | G | 2 | 1 | 2 | 776 | 0 | 0.0026 | G | 0.0026 |  |
| chr11 | rs116461880 | 75029775 | A | G | 2 | 1 | 2 | 776 | 0 | 0.0026 | G | 0.0026 |  |
| chr11 | rs116580074 | 75023899 | T | C | 2 | 1 | 2 | 776 | 0 | 0.0026 | C | 0.0026 |  |
| chr11 | rs116960251 | 75003929 | C | T | 2 | 1 | 2 | 776 | 0 | 0.0026 | T | 0.0026 |  |
| chr11 | rs12273173 | 74993805 | T | A | 2 | 1 | 2 | 776 | 0 | 0.0026 | A | 0.0026 |  |
| chr11 | rs12721496 | 74992340 | G | A | 2 | 1 | 2 | 776 | 0 | 0.0026 | A | 0.0026 |  |
| chr11 | rs137981707 | 75036914 | C | A | 2 | 1 | 2 | 776 | 0 | 0.0026 | A | 0.0026 |  |
| chr11 | rs138247515 | 75005087 | C | T | 2 | 1 | 2 | 776 | 0 | 0.0026 | T | 0.0026 |  |
| chr11 | rs140241121 | 75003595 | A | G | 2 | 1 | 2 | 776 | 0 | 0.0026 | G | 0.0026 |  |
| chr11 | rs141657165 | 75055494 | T | C | 2 | 1 | 2 | 776 | 0 | 0.0026 | C | 0.0026 |  |
| chr11 | rs142776460 | 75018558 | C | G | 2 | 1 | 2 | 776 | 0 | 0.0026 | G | 0.0026 |  |
| chr11 | rs143603291 | 75041251 | A | C | 2 | 1 | 2 | 776 | 0 | 0.0026 | C | 0.0026 |  |
| chr11 | rs144250148 | 75035430 | C | T | 2 | 1 | 2 | 776 | 0 | 0.0026 | T | 0.0026 |  |
| chr11 | rs145065114 | 75019283 | C | T | 2 | 1 | 2 | 776 | 0 | 0.0026 | T | 0.0026 |  |
| chr11 | rs146574519 | 74984894 | A | T | 2 | 1 | 2 | 776 | 0 | 0.0026 | T | 0.0026 |  |
| chr11 | rs148601369 | 74977908 | G | C | 2 | 1 | 2 | 776 | 0 | 0.0026 | C | 0.0026 |  |
| chr11 | rs149102158 | 75003930 | G | A | 2 | 1 | 2 | 776 | 0 | 0.0026 | A | 0.0026 |  |
| chr11 | rs150423823 | 74972410 | GTTC | G | 2 | 1 | 2 | 776 | 0 | 0.0026 | G | 0.0026 |  |
| chr11 | rs150783164 | 74983240 | A | G | 1 | 1 | 2 | 776 | 1 | 0.0026 | G | 0.0026 |  |
| chr11 | rs17133939 | 75054176 | G | A | 2 | 1 | 2 | 776 | 0 | 0.0026 | A | 0.0026 |  |
| chr11 | rs183317119 | 75039164 | G | C | 2 | 1 | 2 | 776 | 0 | 0.0026 | C | 0.0026 |  |
| chr11 | rs183827296 | 75028065 | G | A | 2 | 1 | 2 | 776 | 0 | 0.0026 | A | 0.0026 |  |
| chr11 | rs183991201 | 74999413 | G | A | 2 | 1 | 2 | 776 | 0 | 0.0026 | A | 0.0026 |  |
| chr11 | rs185421129 | 74986251 | T | G | 2 | 1 | 2 | 776 | 0 | 0.0026 | G | 0.0026 |  |
| chr11 | rs185921027 | 74983731 | A | G | 2 | 1 | 2 | 776 | 0 | 0.0026 | G | 0.0026 |  |
| chr11 | rs186575527 | 74987514 | T | C | 2 | 1 | 2 | 776 | 0 | 0.0026 | C | 0.0026 |  |
| chr11 | rs186835182 | 75013957 | A | C | 2 | 1 | 2 | 776 | 0 | 0.0026 | C | 0.0026 |  |
| chr11 | rs187106553 | 75025539 | G | T | 2 | 1 | 2 | 776 | 0 | 0.0026 | T | 0.0026 |  |
| chr11 | rs188699083 | 75021236 | G | C | 2 | 1 | 2 | 776 | 0 | 0.0026 | C | 0.0026 |  |
| chr11 | rs189085145 | 75031506 | G | C | 2 | 1 | 2 | 776 | 0 | 0.0026 | C | 0.0026 |  |
| chr11 | rs189670915 | 74971654 | G | T | 2 | 1 | 2 | 776 | 0 | 0.0026 | T | 0.0026 |  |
| chr11 | rs189757727 | 75044517 | G | C | 2 | 1 | 2 | 776 | 0 | 0.0026 | C | 0.0026 |  |
| chr11 | rs190663506 | 75056582 | A | G | 2 | 1 | 2 | 776 | 0 | 0.0026 | G | 0.0026 |  |
| chr11 | rs193194500 | 75024902 | A | G | 2 | 1 | 2 | 776 | 0 | 0.0026 | G | 0.0026 |  |
| chr11 | rs34601759 | 74975132 | G | T | 2 | 1 | 2 | 776 | 0 | 0.0026 | T | 0.0026 |  |
| chr11 | rs35615112 | 74980351 | C | G | 2 | 1 | 2 | 776 | 0 | 0.0026 | G | 0.0026 |  |
| chr11 | rs35773729 | 74989266 | G | T | 2 | 1 | 2 | 776 | 0 | 0.0026 | T | 0.0026 |  |
| chr11 | rs36004313 | 74982922 | C | T | 2 | 1 | 2 | 776 | 0 | 0.0026 | T | 0.0026 |  |
| chr11 | rs36006020 | 74991373 | T | C | 2 | 1 | 2 | 776 | 0 | 0.0026 | C | 0.0026 |  |
| chr11 | rs368853912 | 75019876 | G | T | 2 | 1 | 2 | 776 | 0 | 0.0026 | T | 0.0026 |  |
| chr11 | rs372840252 | 75001729 | C | T | 2 | 1 | 2 | 776 | 0 | 0.0026 | T | 0.0026 |  |
| chr11 | rs372937722 | 75001886 | C | T | 2 | 1 | 2 | 776 | 0 | 0.0026 | T | 0.0026 |  |
| chr11 | rs373388997 | 75020210 | A | G | 2 | 1 | 2 | 776 | 0 | 0.0026 | G | 0.0026 |  |
| chr11 | rs376640557 | 74998928 | T | C | 2 | 1 | 2 | 776 | 0 | 0.0026 | C | 0.0026 |  |
| chr11 | rs376699998 | 75005807 | A | G | 2 | 1 | 2 | 776 | 0 | 0.0026 | G | 0.0026 |  |
| chr11 | rs376785972 | 75030688 | T | C | 2 | 1 | 2 | 776 | 0 | 0.0026 | C | 0.0026 |  |
| chr11 | rs55794814 | 74989799 | T | C | 2 | 1 | 2 | 776 | 0 | 0.0026 | C | 0.0026 |  |
| chr11 | rs73490793 | 75030608 | G | A | 2 | 1 | 2 | 776 | 0 | 0.0026 | A | 0.0026 |  |
| chr11 | rs73490797 | 75033191 | G | A | 2 | 1 | 2 | 776 | 0 | 0.0026 | A | 0.0026 |  |
| chr11 | rs78423055 | 75054512 | C | G | 2 | 1 | 2 | 776 | 0 | 0.0026 | G | 0.0026 |  |
| chr11 | rs79859519 | 74976322 | C | T | 2 | 1 | 2 | 776 | 0 | 0.0026 | T | 0.0026 |  |
| chr11 | rs80196420 | 75004097 | G | A | 2 | 1 | 2 | 776 | 0 | 0.0026 | A | 0.0026 |  |
| chr11 | rs9804521 | 74996471 | T | G | 2 | 1 | 2 | 776 | 0 | 0.0026 | G | 0.0026 |  |
| chr11 | NA | 74976238 | G | A | 2 | 1 | 2 | 776 | 0 | 0.0026 | A | 0.0026 |  |
| chr11 | NA | 74979131 | T | C | 2 | 1 | 2 | 776 | 0 | 0.0026 | C | 0.0026 |  |
| chr11 | NA | 74979581 | G | A | 2 | 1 | 2 | 776 | 0 | 0.0026 | A | 0.0026 |  |
| chr11 | NA | 74979584 | G | A | 2 | 1 | 2 | 776 | 0 | 0.0026 | A | 0.0026 |  |
| chr11 | NA | 74984907 | A | T | 2 | 1 | 2 | 776 | 0 | 0.0026 | T | 0.0026 |  |
| chr11 | NA | 74986520 | G | A | 2 | 1 | 2 | 776 | 0 | 0.0026 | A | 0.0026 |  |
| chr11 | NA | 75047692 | C | T | 2 | 1 | 2 | 776 | 0 | 0.0026 | T | 0.0026 |  |
| chr11 | NA | 75053089 | T | C | 2 | 1 | 2 | 776 | 0 | 0.0026 | C | 0.0026 |  |
| chr11 | NA | 75059346 | C | G | 2 | 1 | 2 | 776 | 0 | 0.0026 | G | 0.0026 |  |
| chr11 | NA | 74987178 | A | G | 2 | 1 | 2 | 776 | 0 | 0.0026 | G | 0.0026 |  |
| chr11 | NA | 74988260 | G | C | 2 | 1 | 2 | 776 | 0 | 0.0026 | C | 0.0026 |  |
| chr11 | NA | 74991985 | T | TG | 2 | 1 | 2 | 776 | 0 | 0.0026 | TG | 0.0026 |  |
| chr11 | NA | 74999238 | A | G | 2 | 1 | 2 | 776 | 0 | 0.0026 | G | 0.0026 |  |
| chr11 | NA | 75000067 | G | A | 2 | 1 | 2 | 776 | 0 | 0.0026 | A | 0.0026 |  |
| chr11 | NA | 75000552 | C | T | 1 | 1 | 2 | 776 | 1 | 0.0026 | T | 0.0026 |  |
| chr11 | NA | 75001511 | C | T | 2 | 1 | 2 | 776 | 0 | 0.0026 | T | 0.0026 |  |
| chr11 | NA | 75001576 | C | T | 2 | 1 | 2 | 776 | 0 | 0.0026 | T | 0.0026 |  |
| chr11 | NA | 75003939 | T | TC | 2 | 1 | 2 | 776 | 0 | 0.0026 | TC | 0.0026 |  |
| chr11 | NA | 75004915 | G | C | 2 | 1 | 2 | 776 | 0 | 0.0026 | C | 0.0026 |  |
| chr11 | NA | 75005127 | T | C | 2 | 1 | 2 | 776 | 0 | 0.0026 | C | 0.0026 |  |
| chr11 | NA | 75007989 | C | A | 2 | 1 | 2 | 776 | 0 | 0.0026 | A | 0.0026 |  |
| chr11 | NA | 75009464 | T | C | 2 | 1 | 2 | 776 | 0 | 0.0026 | C | 0.0026 |  |
| chr11 | NA | 75009554 | T | C | 2 | 1 | 2 | 776 | 0 | 0.0026 | C | 0.0026 |  |
| chr11 | NA | 75014828 | A | G | 2 | 1 | 2 | 776 | 0 | 0.0026 | G | 0.0026 |  |
| chr11 | NA | 75015338 | T | C | 2 | 1 | 2 | 776 | 0 | 0.0026 | C | 0.0026 |  |
| chr11 | NA | 75015572 | T | C | 2 | 1 | 2 | 776 | 0 | 0.0026 | C | 0.0026 |  |
| chr11 | NA | 75017948 | C | A | 2 | 1 | 2 | 776 | 0 | 0.0026 | A | 0.0026 |  |
| chr11 | NA | 75019163 | G | A | 2 | 1 | 2 | 776 | 0 | 0.0026 | A | 0.0026 |  |
| chr11 | NA | 75028868 | G | C | 2 | 1 | 2 | 776 | 0 | 0.0026 | C | 0.0026 |  |
| chr11 | NA | 75029916 | A | G | 2 | 1 | 2 | 776 | 0 | 0.0026 | G | 0.0026 |  |
| chr11 | NA | 75031557 | G | T | 2 | 1 | 2 | 776 | 0 | 0.0026 | T | 0.0026 |  |
| chr11 | NA | 75032210 | G | A | 2 | 1 | 2 | 776 | 0 | 0.0026 | A | 0.0026 |  |
| chr11 | NA | 75038549 | C | T | 2 | 1 | 2 | 776 | 0 | 0.0026 | T | 0.0026 |  |
| chr11 | NA | 75038643 | G | GGTTTT | 1 | 1 | 2 | 776 | 1 | 0.0026 | GGTTTT | 0.0026 |  |
| chr11 | NA | 75040602 | G | A | 2 | 1 | 2 | 776 | 0 | 0.0026 | A | 0.0026 |  |
| chr11 | NA | 75047556 | G | A | 2 | 1 | 2 | 776 | 0 | 0.0026 | A | 0.0026 |  |
| chr11 | NA | 75057886 | G | A | 2 | 1 | 2 | 776 | 0 | 0.0026 | A | 0.0026 |  |
| chr11 | NA | 75060269 | G | T | 2 | 1 | 2 | 776 | 0 | 0.0026 | T | 0.0026 |  |
| chr11 | NA | 75009817 | GA | G | 2 | 1 | 2 | 776 | 0 | 0.0026 | G | 0.0026 |  |
| chr11 | NA | 75010007 | C | A | 2 | 1 | 2 | 776 | 0 | 0.0026 | A | 0.0026 |  |
| chr11 | NA | 75028058 | C | T | 2 | 1 | 2 | 776 | 0 | 0.0026 | T | 0.0026 |  |
| chr11 | NA | 75059043 | C | T | 2 | 1 | 2 | 776 | 0 | 0.0026 | T | 0.0026 |  |
| chr11 | NA | 75027368 | C | CA | 90 | 0.0111 | 1 | 598 | 0 | 0.0017 | CA | 0.0017 |  |
| chr11 | NA | 75040132 | CTT | C | 45 | 0.0222 | 1 | 688 | 0 | 0.0015 | C | 0.0015 |  |
| chr11 | NA | 75007229 | C | CAA | 36 | 0.0278 | 1 | 706 | 0 | 0.0014 | CAA | 0.0014 |  |
| chr11 | NA | 74999660 | CT | C | 34 | 0.0294 | 1 | 710 | 0 | 0.0014 | C | 0.0014 |  |
| chr11 | NA | 75007229 | C | CAAA | 25 | 0.04 | 1 | 728 | 0 | 0.0014 | CAAA | 0.0014 |  |
| chr11 | NA | 75019788 | C | CA | 20 | 0.05 | 1 | 738 | 0 | 0.0014 | CA | 0.0014 |  |
| chr11 | NA | 74972225 | CGTGTGTGT | C | 16 | 0.0625 | 1 | 746 | 0 | 0.0013 | C | 0.0013 |  |
| chr11 | rs11236394 | 75038644 | T | G | 13 | 0.0769 | 1 | 752 | 0 | 0.0013 | G | 0.0013 |  |
| chr11 | NA | 74972225 | CGTGTGTGTGTGT | C | 13 | 0.0769 | 1 | 752 | 0 | 0.0013 | C | 0.0013 |  |
| chr11 | NA | 75032309 | A | AC | 9 | 0.1111 | 1 | 760 | 0 | 0.0013 | AC | 0.0013 |  |
| chr11 | NA | 75007229 | C | CAAAA | 8 | 0.125 | 1 | 762 | 0 | 0.0013 | CAAAA | 0.0013 |  |
| chr11 | NA | 74972225 | C | CGT | 7 | 0.1429 | 1 | 764 | 0 | 0.0013 | CGT | 0.0013 |  |
| chr11 | NA | 75038643 | G | GTT | 7 | 0.1429 | 1 | 764 | 0 | 0.0013 | GTT | 0.0013 |  |
| chr11 | rs199578338 | 75010281 | T | TA | 4 | 0.25 | 1 | 770 | 0 | 0.0013 | TA | 0.0013 |  |
| chr11 | rs375322039 | 74979587 | G | A | 4 | 0.25 | 1 | 770 | 0 | 0.0013 | A | 0.0013 |  |
| chr11 | rs536750 | 75049534 | G | C | 388 | 0.9948 | 771 | 772 | 385 | 0.9987 | G | 0.0013 |  |
| chr11 | rs111561448 | 75028328 | G | A | 3 | 0.3333 | 1 | 772 | 0 | 0.0013 | A | 0.0013 |  |
| chr11 | rs188176869 | 75047429 | C | T | 3 | 0.3333 | 1 | 772 | 0 | 0.0013 | T | 0.0013 |  |
| chr11 | rs373200031 | 75006903 | G | A | 3 | 0.3333 | 1 | 772 | 0 | 0.0013 | A | 0.0013 |  |
| chr11 | NA | 74974329 | G | GT | 3 | 0.3333 | 1 | 772 | 0 | 0.0013 | GT | 0.0013 |  |
| chr11 | NA | 75006291 | C | A | 3 | 0.3333 | 1 | 772 | 0 | 0.0013 | A | 0.0013 |  |
| chr11 | NA | 75006293 | C | A | 3 | 0.3333 | 1 | 772 | 0 | 0.0013 | A | 0.0013 |  |
| chr11 | NA | 75007255 | T | A | 3 | 0.3333 | 1 | 772 | 0 | 0.0013 | A | 0.0013 |  |
| chr11 | NA | 75007254 | G | A | 3 | 0.3333 | 1 | 772 | 0 | 0.0013 | A | 0.0013 |  |
| chr11 | NA | 75008936 | ATT | A | 3 | 0.3333 | 1 | 772 | 0 | 0.0013 | A | 0.0013 |  |
| chr11 | rs114033091 | 75030842 | C | T | 2 | 0.5 | 1 | 774 | 0 | 0.0013 | T | 0.0013 |  |
| chr11 | rs114759537 | 75043975 | C | A | 2 | 0.5 | 1 | 774 | 0 | 0.0013 | A | 0.0013 |  |
| chr11 | rs117959018 | 75020872 | G | A | 2 | 0.5 | 1 | 774 | 0 | 0.0013 | A | 0.0013 |  |
| chr11 | rs11822845 | 75007233 | A | T | 2 | 0.5 | 1 | 774 | 0 | 0.0013 | T | 0.0013 |  |
| chr11 | rs147940139 | 75031697 | C | G | 2 | 0.5 | 1 | 774 | 0 | 0.0013 | G | 0.0013 |  |
| chr11 | rs190430655 | 75017665 | C | T | 2 | 0.5 | 1 | 774 | 0 | 0.0013 | T | 0.0013 |  |
| chr11 | rs35951757 | 74994827 | G | T | 2 | 0.5 | 1 | 774 | 0 | 0.0013 | T | 0.0013 |  |
| chr11 | rs74482654 | 75047145 | C | A | 2 | 0.5 | 1 | 774 | 0 | 0.0013 | A | 0.0013 |  |
| chr11 | rs75603766 | 75012645 | G | C | 2 | 0.5 | 1 | 774 | 0 | 0.0013 | C | 0.0013 |  |
| chr11 | rs77891894 | 74979666 | A | G | 2 | 0.5 | 1 | 774 | 0 | 0.0013 | G | 0.0013 |  |
| chr11 | NA | 74972250 | G | GTGTGTA | 2 | 0.5 | 1 | 774 | 0 | 0.0013 | GTGTGTA | 0.0013 |  |
| chr11 | NA | 74987424 | G | T | 2 | 0.5 | 1 | 774 | 0 | 0.0013 | T | 0.0013 |  |
| chr11 | NA | 74999203 | G | A | 2 | 0.5 | 1 | 774 | 0 | 0.0013 | A | 0.0013 |  |
| chr11 | NA | 75014289 | G | A | 2 | 0.5 | 1 | 774 | 0 | 0.0013 | A | 0.0013 |  |
| chr11 | NA | 74972225 | C | CGTGT | 2 | 0.5 | 1 | 774 | 0 | 0.0013 | CGTGT | 0.0013 |  |
| chr11 | NA | 75032511 | C | T | 2 | 0.5 | 1 | 774 | 0 | 0.0013 | T | 0.0013 |  |
| chr11 | NA | 75039818 | G | A | 2 | 0.5 | 1 | 774 | 0 | 0.0013 | A | 0.0013 |  |
| chr11 | NA | 75043116 | C | T | 2 | 0.5 | 1 | 774 | 0 | 0.0013 | T | 0.0013 |  |
| chr11 | NA | 75048928 | G | A | 2 | 0.5 | 1 | 774 | 0 | 0.0013 | A | 0.0013 |  |
| chr11 | NA | 75041241 | G | C | 2 | 0.5 | 1 | 774 | 0 | 0.0013 | C | 0.0013 |  |
| chr11 | rs111523925 | 74978912 | C | G | 1 | 1 | 1 | 776 | 0 | 0.0013 | G | 0.0013 |  |
| chr11 | rs111660907 | 74980054 | G | A | 1 | 1 | 1 | 776 | 0 | 0.0013 | A | 0.0013 |  |
| chr11 | rs111802915 | 75055860 | A | G | 1 | 1 | 1 | 776 | 0 | 0.0013 | G | 0.0013 |  |
| chr11 | rs112483895 | 74989642 | G | A | 1 | 1 | 1 | 776 | 0 | 0.0013 | A | 0.0013 |  |
| chr11 | rs112699618 | 75054781 | A | G | 1 | 1 | 1 | 776 | 0 | 0.0013 | G | 0.0013 |  |
| chr11 | rs112714455 | 75025592 | T | G | 1 | 1 | 1 | 776 | 0 | 0.0013 | G | 0.0013 |  |
| chr11 | rs112923264 | 75055794 | C | T | 1 | 1 | 1 | 776 | 0 | 0.0013 | T | 0.0013 |  |
| chr11 | rs113010308 | 75059040 | C | G | 1 | 1 | 1 | 776 | 0 | 0.0013 | G | 0.0013 |  |
| chr11 | rs113313443 | 75046410 | C | T | 1 | 1 | 1 | 776 | 0 | 0.0013 | T | 0.0013 |  |
| chr11 | rs113384806 | 75054140 | G | A | 1 | 1 | 1 | 776 | 0 | 0.0013 | A | 0.0013 |  |
| chr11 | rs113608885 | 75055701 | G | C | 1 | 1 | 1 | 776 | 0 | 0.0013 | C | 0.0013 |  |
| chr11 | rs114301150 | 75037817 | G | A | 1 | 1 | 1 | 776 | 0 | 0.0013 | A | 0.0013 |  |
| chr11 | rs114320459 | 75027981 | G | A | 1 | 1 | 1 | 776 | 0 | 0.0013 | A | 0.0013 |  |
| chr11 | rs114448343 | 75052012 | C | T | 1 | 1 | 1 | 776 | 0 | 0.0013 | T | 0.0013 |  |
| chr11 | rs114679753 | 75041041 | T | C | 1 | 1 | 1 | 776 | 0 | 0.0013 | C | 0.0013 |  |
| chr11 | rs114685187 | 75033711 | G | A | 1 | 1 | 1 | 776 | 0 | 0.0013 | A | 0.0013 |  |
| chr11 | rs115091664 | 75054171 | C | T | 1 | 1 | 1 | 776 | 0 | 0.0013 | T | 0.0013 |  |
| chr11 | rs115486781 | 75034292 | T | C | 1 | 1 | 1 | 776 | 0 | 0.0013 | C | 0.0013 |  |
| chr11 | rs115616410 | 75053754 | C | T | 1 | 1 | 1 | 776 | 0 | 0.0013 | T | 0.0013 |  |
| chr11 | rs115637731 | 75045084 | T | C | 1 | 1 | 1 | 776 | 0 | 0.0013 | C | 0.0013 |  |
| chr11 | rs116017182 | 75053732 | G | A | 1 | 1 | 1 | 776 | 0 | 0.0013 | A | 0.0013 |  |
| chr11 | rs116096903 | 75043438 | A | G | 1 | 1 | 1 | 776 | 0 | 0.0013 | G | 0.0013 |  |
| chr11 | rs116099324 | 74983493 | C | A | 1 | 1 | 1 | 776 | 0 | 0.0013 | A | 0.0013 |  |
| chr11 | rs116107836 | 74975985 | C | T | 1 | 1 | 1 | 776 | 0 | 0.0013 | T | 0.0013 |  |
| chr11 | rs116353611 | 75035460 | C | T | 1 | 1 | 1 | 776 | 0 | 0.0013 | T | 0.0013 |  |
| chr11 | rs116441878 | 75026690 | G | A | 1 | 1 | 1 | 776 | 0 | 0.0013 | A | 0.0013 |  |
| chr11 | rs116479610 | 74972393 | C | T | 1 | 1 | 1 | 776 | 0 | 0.0013 | T | 0.0013 |  |
| chr11 | rs116564309 | 74975876 | C | T | 1 | 1 | 1 | 776 | 0 | 0.0013 | T | 0.0013 |  |
| chr11 | rs117665064 | 75022519 | A | T | 1 | 1 | 1 | 776 | 0 | 0.0013 | T | 0.0013 |  |
| chr11 | rs117718254 | 75051043 | G | T | 1 | 1 | 1 | 776 | 0 | 0.0013 | T | 0.0013 |  |
| chr11 | rs117720873 | 75029795 | A | T | 1 | 1 | 1 | 776 | 0 | 0.0013 | T | 0.0013 |  |
| chr11 | rs117788237 | 74974074 | C | T | 1 | 1 | 1 | 776 | 0 | 0.0013 | T | 0.0013 |  |
| chr11 | rs11820702 | 74985410 | G | C | 1 | 1 | 1 | 776 | 0 | 0.0013 | C | 0.0013 |  |
| chr11 | rs12277084 | 75030917 | T | C | 1 | 1 | 1 | 776 | 0 | 0.0013 | C | 0.0013 |  |
| chr11 | rs12282407 | 75039701 | A | C | 1 | 1 | 1 | 776 | 0 | 0.0013 | C | 0.0013 |  |
| chr11 | rs12289875 | 75040004 | G | T | 1 | 1 | 1 | 776 | 0 | 0.0013 | T | 0.0013 |  |
| chr11 | rs138563278 | 75029469 | C | T | 1 | 1 | 1 | 776 | 0 | 0.0013 | T | 0.0013 |  |
| chr11 | rs138633653 | 74999907 | G | A | 1 | 1 | 1 | 776 | 0 | 0.0013 | A | 0.0013 |  |
| chr11 | rs138680940 | 75021327 | C | T | 1 | 1 | 1 | 776 | 0 | 0.0013 | T | 0.0013 |  |
| chr11 | rs138942262 | 74981758 | A | G | 1 | 1 | 1 | 776 | 0 | 0.0013 | G | 0.0013 |  |
| chr11 | rs139538894 | 74978807 | G | T | 1 | 1 | 1 | 776 | 0 | 0.0013 | T | 0.0013 |  |
| chr11 | rs139604424 | 74974480 | C | A | 1 | 1 | 1 | 776 | 0 | 0.0013 | A | 0.0013 |  |
| chr11 | rs140226575 | 74978732 | G | A | 1 | 1 | 1 | 776 | 0 | 0.0013 | A | 0.0013 |  |
| chr11 | rs140347328 | 75000148 | G | A | 1 | 1 | 1 | 776 | 0 | 0.0013 | A | 0.0013 |  |
| chr11 | rs140943182 | 74998734 | A | G | 1 | 1 | 1 | 776 | 0 | 0.0013 | G | 0.0013 |  |
| chr11 | rs141122457 | 75036744 | C | T | 1 | 1 | 1 | 776 | 0 | 0.0013 | T | 0.0013 |  |
| chr11 | rs141147574 | 75050932 | A | G | 1 | 1 | 1 | 776 | 0 | 0.0013 | G | 0.0013 |  |
| chr11 | rs141296694 | 75026208 | C | T | 1 | 1 | 1 | 776 | 0 | 0.0013 | T | 0.0013 |  |
| chr11 | rs141340082 | 74972120 | A | G | 1 | 1 | 1 | 776 | 0 | 0.0013 | G | 0.0013 |  |
| chr11 | rs141380706 | 75037683 | C | T | 1 | 1 | 1 | 776 | 0 | 0.0013 | T | 0.0013 |  |
| chr11 | rs142123343 | 74971934 | C | T | 1 | 1 | 1 | 776 | 0 | 0.0013 | T | 0.0013 |  |
| chr11 | rs142215230 | 75015805 | G | A | 1 | 1 | 1 | 776 | 0 | 0.0013 | A | 0.0013 |  |
| chr11 | rs142603821 | 75049247 | A | G | 1 | 1 | 1 | 776 | 0 | 0.0013 | G | 0.0013 |  |
| chr11 | rs142759434 | 75059210 | A | G | 1 | 1 | 1 | 776 | 0 | 0.0013 | G | 0.0013 |  |
| chr11 | rs142983263 | 75002099 | T | C | 1 | 1 | 1 | 776 | 0 | 0.0013 | C | 0.0013 |  |
| chr11 | rs143290928 | 75047006 | G | A | 1 | 1 | 1 | 776 | 0 | 0.0013 | A | 0.0013 |  |
| chr11 | rs143318077 | 74995007 | AAAG | A | 1 | 1 | 1 | 776 | 0 | 0.0013 | A | 0.0013 |  |
| chr11 | rs143842421 | 75005148 | T | C | 1 | 1 | 1 | 776 | 0 | 0.0013 | C | 0.0013 |  |
| chr11 | rs143854609 | 75021178 | G | A | 1 | 1 | 1 | 776 | 0 | 0.0013 | A | 0.0013 |  |
| chr11 | rs143898884 | 75001709 | C | T | 1 | 1 | 1 | 776 | 0 | 0.0013 | T | 0.0013 |  |
| chr11 | rs143931124 | 75043417 | C | A | 1 | 1 | 1 | 776 | 0 | 0.0013 | A | 0.0013 |  |
| chr11 | rs144192249 | 75061044 | C | A | 1 | 1 | 1 | 776 | 0 | 0.0013 | A | 0.0013 |  |
| chr11 | rs144763180 | 75040838 | A | G | 1 | 1 | 1 | 776 | 0 | 0.0013 | G | 0.0013 |  |
| chr11 | rs144828866 | 75037267 | C | T | 1 | 1 | 1 | 776 | 0 | 0.0013 | T | 0.0013 |  |
| chr11 | rs145101792 | 75057603 | C | T | 1 | 1 | 1 | 776 | 0 | 0.0013 | T | 0.0013 |  |
| chr11 | rs145394042 | 74992181 | G | A | 1 | 1 | 1 | 776 | 0 | 0.0013 | A | 0.0013 |  |
| chr11 | rs145518846 | 74974479 | C | A | 1 | 1 | 1 | 776 | 0 | 0.0013 | A | 0.0013 |  |
| chr11 | rs146343268 | 75010295 | A | C | 1 | 1 | 1 | 776 | 0 | 0.0013 | C | 0.0013 |  |
| chr11 | rs146394424 | 75015995 | T | C | 1 | 1 | 1 | 776 | 0 | 0.0013 | C | 0.0013 |  |
| chr11 | rs147289743 | 74998089 | A | G | 1 | 1 | 1 | 776 | 0 | 0.0013 | G | 0.0013 |  |
| chr11 | rs147414171 | 75055760 | T | A | 1 | 1 | 1 | 776 | 0 | 0.0013 | A | 0.0013 |  |
| chr11 | rs147602750 | 74977234 | G | A | 1 | 1 | 1 | 776 | 0 | 0.0013 | A | 0.0013 |  |
| chr11 | rs147833380 | 75013549 | C | T | 1 | 1 | 1 | 776 | 0 | 0.0013 | T | 0.0013 |  |
| chr11 | rs148335621 | 75047108 | T | A | 1 | 1 | 1 | 776 | 0 | 0.0013 | A | 0.0013 |  |
| chr11 | rs148455764 | 75060060 | C | T | 1 | 1 | 1 | 776 | 0 | 0.0013 | T | 0.0013 |  |
| chr11 | rs148983627 | 75062182 | G | A | 1 | 1 | 1 | 776 | 0 | 0.0013 | A | 0.0013 |  |
| chr11 | rs149660460 | 75036787 | G | A | 1 | 1 | 1 | 776 | 0 | 0.0013 | A | 0.0013 |  |
| chr11 | rs149732534 | 74979604 | C | T | 1 | 1 | 1 | 776 | 0 | 0.0013 | T | 0.0013 |  |
| chr11 | rs149963586 | 75056341 | G | A | 1 | 1 | 1 | 776 | 0 | 0.0013 | A | 0.0013 |  |
| chr11 | rs150272347 | 75025387 | G | A | 1 | 1 | 1 | 776 | 0 | 0.0013 | A | 0.0013 |  |
| chr11 | rs150360014 | 75045777 | A | G | 1 | 1 | 1 | 776 | 0 | 0.0013 | G | 0.0013 |  |
| chr11 | rs150413893 | 75014894 | G | A | 1 | 1 | 1 | 776 | 0 | 0.0013 | A | 0.0013 |  |
| chr11 | rs150658125 | 75043420 | T | C | 1 | 1 | 1 | 776 | 0 | 0.0013 | C | 0.0013 |  |
| chr11 | rs180884659 | 74975771 | A | G | 1 | 1 | 1 | 776 | 0 | 0.0013 | G | 0.0013 |  |
| chr11 | rs182804715 | 74989085 | A | G | 1 | 1 | 1 | 776 | 0 | 0.0013 | G | 0.0013 |  |
| chr11 | rs182978023 | 75027727 | C | T | 1 | 1 | 1 | 776 | 0 | 0.0013 | T | 0.0013 |  |
| chr11 | rs183114825 | 75012834 | C | T | 1 | 1 | 1 | 776 | 0 | 0.0013 | T | 0.0013 |  |
| chr11 | rs183246733 | 74974126 | G | A | 1 | 1 | 1 | 776 | 0 | 0.0013 | A | 0.0013 |  |
| chr11 | rs183252977 | 74997658 | C | T | 1 | 1 | 1 | 776 | 0 | 0.0013 | T | 0.0013 |  |
| chr11 | rs183499156 | 75026328 | C | T | 1 | 1 | 1 | 776 | 0 | 0.0013 | T | 0.0013 |  |
| chr11 | rs183756852 | 74995776 | C | T | 1 | 1 | 1 | 776 | 0 | 0.0013 | T | 0.0013 |  |
| chr11 | rs184077284 | 75002742 | C | T | 1 | 1 | 1 | 776 | 0 | 0.0013 | T | 0.0013 |  |
| chr11 | rs184617408 | 75022109 | G | A | 1 | 1 | 1 | 776 | 0 | 0.0013 | A | 0.0013 |  |
| chr11 | rs185859224 | 75026711 | G | A | 1 | 1 | 1 | 776 | 0 | 0.0013 | A | 0.0013 |  |
| chr11 | rs186562890 | 75022545 | T | C | 1 | 1 | 1 | 776 | 0 | 0.0013 | C | 0.0013 |  |
| chr11 | rs186857233 | 75023784 | T | C | 1 | 1 | 1 | 776 | 0 | 0.0013 | C | 0.0013 |  |
| chr11 | rs187028198 | 75060913 | G | A | 1 | 1 | 1 | 776 | 0 | 0.0013 | A | 0.0013 |  |
| chr11 | rs187491258 | 75036896 | T | C | 1 | 1 | 1 | 776 | 0 | 0.0013 | C | 0.0013 |  |
| chr11 | rs188330004 | 74994913 | G | A | 1 | 1 | 1 | 776 | 0 | 0.0013 | A | 0.0013 |  |
| chr11 | rs189237492 | 74986667 | C | G | 1 | 1 | 1 | 776 | 0 | 0.0013 | G | 0.0013 |  |
| chr11 | rs190086534 | 75006703 | A | G | 1 | 1 | 1 | 776 | 0 | 0.0013 | G | 0.0013 |  |
| chr11 | rs190182077 | 75048365 | G | A | 1 | 1 | 1 | 776 | 0 | 0.0013 | A | 0.0013 |  |
| chr11 | rs190750754 | 75034371 | T | C | 1 | 1 | 1 | 776 | 0 | 0.0013 | C | 0.0013 |  |
| chr11 | rs190784710 | 75008144 | A | C | 1 | 1 | 1 | 776 | 0 | 0.0013 | C | 0.0013 |  |
| chr11 | rs192579986 | 75062363 | C | A | 1 | 1 | 1 | 776 | 0 | 0.0013 | A | 0.0013 |  |
| chr11 | rs192817513 | 75039991 | G | A | 1 | 1 | 1 | 776 | 0 | 0.0013 | A | 0.0013 |  |
| chr11 | rs193276399 | 75019899 | G | A | 1 | 1 | 1 | 776 | 0 | 0.0013 | A | 0.0013 |  |
| chr11 | rs200113678 | 75062431 | C | CG | 1 | 1 | 1 | 776 | 0 | 0.0013 | CG | 0.0013 |  |
| chr11 | rs2276309 | 74982924 | C | T | 1 | 1 | 1 | 776 | 0 | 0.0013 | T | 0.0013 |  |
| chr11 | rs28687723 | 75032316 | C | A | 1 | 1 | 1 | 776 | 0 | 0.0013 | A | 0.0013 |  |
| chr11 | rs34207030 | 74977884 | A | G | 1 | 1 | 1 | 776 | 0 | 0.0013 | G | 0.0013 |  |
| chr11 | rs34279285 | 75002805 | G | A | 1 | 1 | 1 | 776 | 0 | 0.0013 | A | 0.0013 |  |
| chr11 | rs34348607 | 74999410 | C | T | 1 | 1 | 1 | 776 | 0 | 0.0013 | T | 0.0013 |  |
| chr11 | rs34386985 | 75062736 | C | A | 1 | 1 | 1 | 776 | 0 | 0.0013 | A | 0.0013 |  |
| chr11 | rs34439199 | 74982987 | C | T | 1 | 1 | 1 | 776 | 0 | 0.0013 | T | 0.0013 |  |
| chr11 | rs34459470 | 74991840 | C | T | 1 | 1 | 1 | 776 | 0 | 0.0013 | T | 0.0013 |  |
| chr11 | rs34462044 | 74978111 | C | T | 1 | 1 | 1 | 776 | 0 | 0.0013 | T | 0.0013 |  |
| chr11 | rs34522100 | 74978031 | T | C | 1 | 1 | 1 | 776 | 0 | 0.0013 | C | 0.0013 |  |
| chr11 | rs34749024 | 75061150 | G | T | 1 | 1 | 1 | 776 | 0 | 0.0013 | T | 0.0013 |  |
| chr11 | rs34891647 | 74997388 | C | A | 1 | 1 | 1 | 776 | 0 | 0.0013 | A | 0.0013 |  |
| chr11 | rs34916046 | 75000537 | C | A | 1 | 1 | 1 | 776 | 0 | 0.0013 | A | 0.0013 |  |
| chr11 | rs34931451 | 74986487 | T | C | 1 | 1 | 1 | 776 | 0 | 0.0013 | C | 0.0013 |  |
| chr11 | rs34972932 | 75001116 | C | G | 1 | 1 | 1 | 776 | 0 | 0.0013 | G | 0.0013 |  |
| chr11 | rs34983463 | 74997929 | T | C | 1 | 1 | 1 | 776 | 0 | 0.0013 | C | 0.0013 |  |
| chr11 | rs35011100 | 74978868 | G | C | 1 | 1 | 1 | 776 | 0 | 0.0013 | C | 0.0013 |  |
| chr11 | rs35013364 | 74994133 | C | T | 1 | 1 | 1 | 776 | 0 | 0.0013 | T | 0.0013 |  |
| chr11 | rs35089648 | 74978205 | G | A | 1 | 1 | 1 | 776 | 0 | 0.0013 | A | 0.0013 |  |
| chr11 | rs35316816 | 74975614 | A | G | 1 | 1 | 1 | 776 | 0 | 0.0013 | G | 0.0013 |  |
| chr11 | rs35336923 | 74994695 | G | A | 1 | 1 | 1 | 776 | 0 | 0.0013 | A | 0.0013 |  |
| chr11 | rs35404251 | 75061071 | G | C | 1 | 1 | 1 | 776 | 0 | 0.0013 | C | 0.0013 |  |
| chr11 | rs35753294 | 74996201 | A | C | 1 | 1 | 1 | 776 | 0 | 0.0013 | C | 0.0013 |  |
| chr11 | rs35991329 | 74980677 | T | C | 1 | 1 | 1 | 776 | 0 | 0.0013 | C | 0.0013 |  |
| chr11 | rs368594040 | 75057244 | A | G | 1 | 1 | 1 | 776 | 0 | 0.0013 | G | 0.0013 |  |
| chr11 | rs369252668 | 75059926 | G | A | 1 | 1 | 1 | 776 | 0 | 0.0013 | A | 0.0013 |  |
| chr11 | rs369532753 | 74988021 | G | C | 1 | 1 | 1 | 776 | 0 | 0.0013 | C | 0.0013 |  |
| chr11 | rs371522697 | 74977902 | G | T | 1 | 1 | 1 | 776 | 0 | 0.0013 | T | 0.0013 |  |
| chr11 | rs371911812 | 74978744 | G | A | 1 | 1 | 1 | 776 | 0 | 0.0013 | A | 0.0013 |  |
| chr11 | rs372282476 | 75025033 | C | T | 1 | 1 | 1 | 776 | 0 | 0.0013 | T | 0.0013 |  |
| chr11 | rs372692055 | 75030595 | C | T | 1 | 1 | 1 | 776 | 0 | 0.0013 | T | 0.0013 |  |
| chr11 | rs373356730 | 75013303 | C | T | 1 | 1 | 1 | 776 | 0 | 0.0013 | T | 0.0013 |  |
| chr11 | rs374327011 | 75001118 | C | T | 1 | 1 | 1 | 776 | 0 | 0.0013 | T | 0.0013 |  |
| chr11 | rs375681001 | 74971503 | G | A | 1 | 1 | 1 | 776 | 0 | 0.0013 | A | 0.0013 |  |
| chr11 | rs375692225 | 75015778 | C | T | 1 | 1 | 1 | 776 | 0 | 0.0013 | T | 0.0013 |  |
| chr11 | rs376754609 | 75025378 | C | T | 1 | 1 | 1 | 776 | 0 | 0.0013 | T | 0.0013 |  |
| chr11 | rs376855923 | 74975171 | G | A | 1 | 1 | 1 | 776 | 0 | 0.0013 | A | 0.0013 |  |
| chr11 | rs377757326 | 75032969 | T | G | 1 | 1 | 1 | 776 | 0 | 0.0013 | G | 0.0013 |  |
| chr11 | rs3832741 | 74972353 | CAAG | C | 1 | 1 | 1 | 776 | 0 | 0.0013 | C | 0.0013 |  |
| chr11 | rs58894572 | 75039297 | C | T | 1 | 1 | 1 | 776 | 0 | 0.0013 | T | 0.0013 |  |
| chr11 | rs60244105 | 75042872 | T | A | 1 | 1 | 1 | 776 | 0 | 0.0013 | A | 0.0013 |  |
| chr11 | rs73490761 | 75003757 | C | G | 1 | 1 | 1 | 776 | 0 | 0.0013 | G | 0.0013 |  |
| chr11 | rs73490789 | 75029338 | A | G | 1 | 1 | 1 | 776 | 0 | 0.0013 | G | 0.0013 |  |
| chr11 | rs73492811 | 75037676 | C | T | 1 | 1 | 1 | 776 | 0 | 0.0013 | T | 0.0013 |  |
| chr11 | rs73492820 | 75046457 | C | T | 1 | 1 | 1 | 776 | 0 | 0.0013 | T | 0.0013 |  |
| chr11 | rs74387284 | 75004128 | G | A | 1 | 1 | 1 | 776 | 0 | 0.0013 | A | 0.0013 |  |
| chr11 | rs75001185 | 75054677 | C | A | 1 | 1 | 1 | 776 | 0 | 0.0013 | A | 0.0013 |  |
| chr11 | rs75674931 | 74971820 | C | A | 1 | 1 | 1 | 776 | 0 | 0.0013 | A | 0.0013 |  |
| chr11 | rs76718999 | 74992557 | T | C | 1 | 1 | 1 | 776 | 0 | 0.0013 | C | 0.0013 |  |
| chr11 | rs76819496 | 75046016 | C | T | 1 | 1 | 1 | 776 | 0 | 0.0013 | T | 0.0013 |  |
| chr11 | rs77878434 | 74989803 | C | T | 1 | 1 | 1 | 776 | 0 | 0.0013 | T | 0.0013 |  |
| chr11 | rs79413526 | 74977354 | C | T | 1 | 1 | 1 | 776 | 0 | 0.0013 | T | 0.0013 |  |
| chr11 | rs80222059 | 75031624 | A | T | 1 | 1 | 1 | 776 | 0 | 0.0013 | T | 0.0013 |  |
| chr11 | NA | 74992506 | G | A | 1 | 1 | 1 | 776 | 0 | 0.0013 | A | 0.0013 |  |
| chr11 | NA | 74983553 | T | C | 1 | 1 | 1 | 776 | 0 | 0.0013 | C | 0.0013 |  |
| chr11 | NA | 74984314 | C | T | 1 | 1 | 1 | 776 | 0 | 0.0013 | T | 0.0013 |  |
| chr11 | NA | 74985038 | G | A | 1 | 1 | 1 | 776 | 0 | 0.0013 | A | 0.0013 |  |
| chr11 | NA | 74985162 | G | A | 1 | 1 | 1 | 776 | 0 | 0.0013 | A | 0.0013 |  |
| chr11 | NA | 74985414 | G | A | 1 | 1 | 1 | 776 | 0 | 0.0013 | A | 0.0013 |  |
| chr11 | NA | 74988019 | G | GTGTCC | 1 | 1 | 1 | 776 | 0 | 0.0013 | GTGTCC | 0.0013 |  |
| chr11 | NA | 74988297 | G | T | 1 | 1 | 1 | 776 | 0 | 0.0013 | T | 0.0013 |  |
| chr11 | NA | 74988573 | C | T | 1 | 1 | 1 | 776 | 0 | 0.0013 | T | 0.0013 |  |
| chr11 | NA | 74989245 | G | A | 1 | 1 | 1 | 776 | 0 | 0.0013 | A | 0.0013 |  |
| chr11 | NA | 74989572 | G | T | 1 | 1 | 1 | 776 | 0 | 0.0013 | T | 0.0013 |  |
| chr11 | NA | 74989575 | G | A | 1 | 1 | 1 | 776 | 0 | 0.0013 | A | 0.0013 |  |
| chr11 | NA | 74989850 | C | T | 1 | 1 | 1 | 776 | 0 | 0.0013 | T | 0.0013 |  |
| chr11 | NA | 74991067 | A | T | 1 | 1 | 1 | 776 | 0 | 0.0013 | T | 0.0013 |  |
| chr11 | NA | 74991269 | A | G | 1 | 1 | 1 | 776 | 0 | 0.0013 | G | 0.0013 |  |
| chr11 | NA | 75024494 | G | A | 1 | 1 | 1 | 776 | 0 | 0.0013 | A | 0.0013 |  |
| chr11 | NA | 74992507 | C | T | 1 | 1 | 1 | 776 | 0 | 0.0013 | T | 0.0013 |  |
| chr11 | NA | 74993363 | C | T | 1 | 1 | 1 | 776 | 0 | 0.0013 | T | 0.0013 |  |
| chr11 | NA | 74993648 | C | T | 1 | 1 | 1 | 776 | 0 | 0.0013 | T | 0.0013 |  |
| chr11 | NA | 74994544 | G | A | 1 | 1 | 1 | 776 | 0 | 0.0013 | A | 0.0013 |  |
| chr11 | NA | 74994978 | C | T | 1 | 1 | 1 | 776 | 0 | 0.0013 | T | 0.0013 |  |
| chr11 | NA | 74995602 | C | T | 1 | 1 | 1 | 776 | 0 | 0.0013 | T | 0.0013 |  |
| chr11 | NA | 74995805 | G | A | 1 | 1 | 1 | 776 | 0 | 0.0013 | A | 0.0013 |  |
| chr11 | NA | 74996203 | T | C | 1 | 1 | 1 | 776 | 0 | 0.0013 | C | 0.0013 |  |
| chr11 | NA | 74997130 | G | A | 1 | 1 | 1 | 776 | 0 | 0.0013 | A | 0.0013 |  |
| chr11 | NA | 74971510 | A | G | 1 | 1 | 1 | 776 | 0 | 0.0013 | G | 0.0013 |  |
| chr11 | NA | 74972588 | G | A | 1 | 1 | 1 | 776 | 0 | 0.0013 | A | 0.0013 |  |
| chr11 | NA | 74972667 | G | A | 1 | 1 | 1 | 776 | 0 | 0.0013 | A | 0.0013 |  |
| chr11 | NA | 74973070 | G | A | 1 | 1 | 1 | 776 | 0 | 0.0013 | A | 0.0013 |  |
| chr11 | NA | 74973286 | A | G | 1 | 1 | 1 | 776 | 0 | 0.0013 | G | 0.0013 |  |
| chr11 | NA | 74974926 | A | G | 1 | 1 | 1 | 776 | 0 | 0.0013 | G | 0.0013 |  |
| chr11 | NA | 74975245 | G | A | 1 | 1 | 1 | 776 | 0 | 0.0013 | A | 0.0013 |  |
| chr11 | NA | 74976081 | G | A | 1 | 1 | 1 | 776 | 0 | 0.0013 | A | 0.0013 |  |
| chr11 | NA | 74977422 | G | A | 1 | 1 | 1 | 776 | 0 | 0.0013 | A | 0.0013 |  |
| chr11 | NA | 74977857 | A | C | 1 | 1 | 1 | 776 | 0 | 0.0013 | C | 0.0013 |  |
| chr11 | NA | 74978247 | G | C | 1 | 1 | 1 | 776 | 0 | 0.0013 | C | 0.0013 |  |
| chr11 | NA | 74979071 | A | G | 1 | 1 | 1 | 776 | 0 | 0.0013 | G | 0.0013 |  |
| chr11 | NA | 74979614 | G | A | 1 | 1 | 1 | 776 | 0 | 0.0013 | A | 0.0013 |  |
| chr11 | NA | 74980131 | C | T | 1 | 1 | 1 | 776 | 0 | 0.0013 | T | 0.0013 |  |
| chr11 | NA | 74980410 | G | A | 1 | 1 | 1 | 776 | 0 | 0.0013 | A | 0.0013 |  |
| chr11 | NA | 74980432 | C | T | 1 | 1 | 1 | 776 | 0 | 0.0013 | T | 0.0013 |  |
| chr11 | NA | 74980433 | G | A | 1 | 1 | 1 | 776 | 0 | 0.0013 | A | 0.0013 |  |
| chr11 | NA | 74980599 | G | A | 1 | 1 | 1 | 776 | 0 | 0.0013 | A | 0.0013 |  |
| chr11 | NA | 74980610 | G | A | 1 | 1 | 1 | 776 | 0 | 0.0013 | A | 0.0013 |  |
| chr11 | NA | 74980670 | G | A | 1 | 1 | 1 | 776 | 0 | 0.0013 | A | 0.0013 |  |
| chr11 | NA | 74981059 | A | C | 1 | 1 | 1 | 776 | 0 | 0.0013 | C | 0.0013 |  |
| chr11 | NA | 74981851 | T | TA | 1 | 1 | 1 | 776 | 0 | 0.0013 | TA | 0.0013 |  |
| chr11 | NA | 74982290 | A | G | 1 | 1 | 1 | 776 | 0 | 0.0013 | G | 0.0013 |  |
| chr11 | NA | 74982324 | G | C | 1 | 1 | 1 | 776 | 0 | 0.0013 | C | 0.0013 |  |
| chr11 | NA | 74982369 | C | T | 1 | 1 | 1 | 776 | 0 | 0.0013 | T | 0.0013 |  |
| chr11 | NA | 74983069 | C | T | 1 | 1 | 1 | 776 | 0 | 0.0013 | T | 0.0013 |  |
| chr11 | NA | 75016232 | C | T | 1 | 1 | 1 | 776 | 0 | 0.0013 | T | 0.0013 |  |
| chr11 | NA | 75017688 | G | A | 1 | 1 | 1 | 776 | 0 | 0.0013 | A | 0.0013 |  |
| chr11 | NA | 75017809 | G | A | 1 | 1 | 1 | 776 | 0 | 0.0013 | A | 0.0013 |  |
| chr11 | NA | 75018235 | C | T | 1 | 1 | 1 | 776 | 0 | 0.0013 | T | 0.0013 |  |
| chr11 | NA | 75018369 | G | A | 1 | 1 | 1 | 776 | 0 | 0.0013 | A | 0.0013 |  |
| chr11 | NA | 75019341 | A | G | 1 | 1 | 1 | 776 | 0 | 0.0013 | G | 0.0013 |  |
| chr11 | NA | 74986551 | G | T | 1 | 1 | 1 | 776 | 0 | 0.0013 | T | 0.0013 |  |
| chr11 | NA | 74987916 | T | A | 1 | 1 | 1 | 776 | 0 | 0.0013 | A | 0.0013 |  |
| chr11 | NA | 75020360 | C | T | 1 | 1 | 1 | 776 | 0 | 0.0013 | T | 0.0013 |  |
| chr11 | NA | 75021515 | G | A | 1 | 1 | 1 | 776 | 0 | 0.0013 | A | 0.0013 |  |
| chr11 | NA | 75022997 | A | G | 1 | 1 | 1 | 776 | 0 | 0.0013 | G | 0.0013 |  |
| chr11 | NA | 74990143 | A | G | 1 | 1 | 1 | 776 | 0 | 0.0013 | G | 0.0013 |  |
| chr11 | NA | 75023498 | G | A | 1 | 1 | 1 | 776 | 0 | 0.0013 | A | 0.0013 |  |
| chr11 | NA | 75023803 | C | G | 1 | 1 | 1 | 776 | 0 | 0.0013 | G | 0.0013 |  |
| chr11 | NA | 74991953 | G | A | 1 | 1 | 1 | 776 | 0 | 0.0013 | A | 0.0013 |  |
| chr11 | NA | 75024248 | T | A | 1 | 1 | 1 | 776 | 0 | 0.0013 | A | 0.0013 |  |
| chr11 | NA | 75048670 | C | CA | 1 | 1 | 1 | 776 | 0 | 0.0013 | CA | 0.0013 |  |
| chr11 | NA | 75024565 | G | A | 1 | 1 | 1 | 776 | 0 | 0.0013 | A | 0.0013 |  |
| chr11 | NA | 75024924 | A | G | 1 | 1 | 1 | 776 | 0 | 0.0013 | G | 0.0013 |  |
| chr11 | NA | 75025107 | C | A | 1 | 1 | 1 | 776 | 0 | 0.0013 | A | 0.0013 |  |
| chr11 | NA | 75025340 | A | G | 1 | 1 | 1 | 776 | 0 | 0.0013 | G | 0.0013 |  |
| chr11 | NA | 75025427 | C | T | 1 | 1 | 1 | 776 | 0 | 0.0013 | T | 0.0013 |  |
| chr11 | NA | 75025451 | A | G | 1 | 1 | 1 | 776 | 0 | 0.0013 | G | 0.0013 |  |
| chr11 | NA | 74996243 | G | A | 1 | 1 | 1 | 776 | 0 | 0.0013 | A | 0.0013 |  |
| chr11 | NA | 74996773 | G | A | 1 | 1 | 1 | 776 | 0 | 0.0013 | A | 0.0013 |  |
| chr11 | NA | 75028348 | C | T | 1 | 1 | 1 | 776 | 0 | 0.0013 | T | 0.0013 |  |
| chr11 | NA | 75028363 | C | A | 1 | 1 | 1 | 776 | 0 | 0.0013 | A | 0.0013 |  |
| chr11 | NA | 74998278 | C | G | 1 | 1 | 1 | 776 | 0 | 0.0013 | G | 0.0013 |  |
| chr11 | NA | 74998579 | A | G | 1 | 1 | 1 | 776 | 0 | 0.0013 | G | 0.0013 |  |
| chr11 | NA | 74998950 | G | A | 1 | 1 | 1 | 776 | 0 | 0.0013 | A | 0.0013 |  |
| chr11 | NA | 74999221 | A | T | 1 | 1 | 1 | 776 | 0 | 0.0013 | T | 0.0013 |  |
| chr11 | NA | 74999318 | C | G | 1 | 1 | 1 | 776 | 0 | 0.0013 | G | 0.0013 |  |
| chr11 | NA | 74999663 | T | TC | 1 | 1 | 1 | 776 | 0 | 0.0013 | TC | 0.0013 |  |
| chr11 | NA | 75000020 | G | T | 1 | 1 | 1 | 776 | 0 | 0.0013 | T | 0.0013 |  |
| chr11 | NA | 75000053 | C | T | 1 | 1 | 1 | 776 | 0 | 0.0013 | T | 0.0013 |  |
| chr11 | NA | 75000237 | T | C | 1 | 1 | 1 | 776 | 0 | 0.0013 | C | 0.0013 |  |
| chr11 | NA | 75000408 | C | A | 1 | 1 | 1 | 776 | 0 | 0.0013 | A | 0.0013 |  |
| chr11 | NA | 75000553 | G | A | 1 | 1 | 1 | 776 | 0 | 0.0013 | A | 0.0013 |  |
| chr11 | NA | 75000879 | T | TCAACTGACAGCTC | 1 | 1 | 1 | 776 | 0 | 0.0013 | TCAACTGACAGCTC | 0.0013 |  |
| chr11 | NA | 75000994 | G | T | 1 | 1 | 1 | 776 | 0 | 0.0013 | T | 0.0013 |  |
| chr11 | NA | 75001133 | G | T | 1 | 1 | 1 | 776 | 0 | 0.0013 | T | 0.0013 |  |
| chr11 | NA | 75001170 | G | C | 1 | 1 | 1 | 776 | 0 | 0.0013 | C | 0.0013 |  |
| chr11 | NA | 75001994 | T | G | 1 | 1 | 1 | 776 | 0 | 0.0013 | G | 0.0013 |  |
| chr11 | NA | 75003042 | G | A | 1 | 1 | 1 | 776 | 0 | 0.0013 | A | 0.0013 |  |
| chr11 | NA | 75003123 | A | G | 1 | 1 | 1 | 776 | 0 | 0.0013 | G | 0.0013 |  |
| chr11 | NA | 75003537 | C | T | 1 | 1 | 1 | 776 | 0 | 0.0013 | T | 0.0013 |  |
| chr11 | NA | 74976150 | C | T | 1 | 1 | 1 | 776 | 0 | 0.0013 | T | 0.0013 |  |
| chr11 | NA | 75004790 | AG | A | 1 | 1 | 1 | 776 | 0 | 0.0013 | A | 0.0013 |  |
| chr11 | NA | 75004906 | C | T | 1 | 1 | 1 | 776 | 0 | 0.0013 | T | 0.0013 |  |
| chr11 | NA | 75004930 | G | A | 1 | 1 | 1 | 776 | 0 | 0.0013 | A | 0.0013 |  |
| chr11 | NA | 75005282 | T | C | 1 | 1 | 1 | 776 | 0 | 0.0013 | C | 0.0013 |  |
| chr11 | NA | 75005651 | A | AAATT | 1 | 1 | 1 | 776 | 0 | 0.0013 | AAATT | 0.0013 |  |
| chr11 | NA | 75006610 | C | A | 1 | 1 | 1 | 776 | 0 | 0.0013 | A | 0.0013 |  |
| chr11 | NA | 75006730 | A | G | 1 | 1 | 1 | 776 | 0 | 0.0013 | G | 0.0013 |  |
| chr11 | NA | 75007229 | C | CAAAAAA | 1 | 1 | 1 | 776 | 0 | 0.0013 | CAAAAAA | 0.0013 |  |
| chr11 | NA | 75007253 | AGT | A | 1 | 1 | 1 | 776 | 0 | 0.0013 | A | 0.0013 |  |
| chr11 | NA | 75008189 | C | T | 1 | 1 | 1 | 776 | 0 | 0.0013 | T | 0.0013 |  |
| chr11 | NA | 75008568 | C | CA | 1 | 1 | 1 | 776 | 0 | 0.0013 | CA | 0.0013 |  |
| chr11 | NA | 74979226 | A | G | 1 | 1 | 1 | 776 | 0 | 0.0013 | G | 0.0013 |  |
| chr11 | NA | 74979578 | A | G | 1 | 1 | 1 | 776 | 0 | 0.0013 | G | 0.0013 |  |
| chr11 | NA | 75010848 | G | A | 1 | 1 | 1 | 776 | 0 | 0.0013 | A | 0.0013 |  |
| chr11 | NA | 74979938 | C | T | 1 | 1 | 1 | 776 | 0 | 0.0013 | T | 0.0013 |  |
| chr11 | NA | 75011562 | G | A | 1 | 1 | 1 | 776 | 0 | 0.0013 | A | 0.0013 |  |
| chr11 | NA | 75012548 | G | A | 1 | 1 | 1 | 776 | 0 | 0.0013 | A | 0.0013 |  |
| chr11 | NA | 75012643 | TGGGTA | T | 1 | 1 | 1 | 776 | 0 | 0.0013 | T | 0.0013 |  |
| chr11 | NA | 75012714 | CAG | C | 1 | 1 | 1 | 776 | 0 | 0.0013 | C | 0.0013 |  |
| chr11 | NA | 75013632 | C | A | 1 | 1 | 1 | 776 | 0 | 0.0013 | A | 0.0013 |  |
| chr11 | NA | 75014311 | G | T | 1 | 1 | 1 | 776 | 0 | 0.0013 | T | 0.0013 |  |
| chr11 | NA | 75014447 | C | A | 1 | 1 | 1 | 776 | 0 | 0.0013 | A | 0.0013 |  |
| chr11 | NA | 75014662 | C | T | 1 | 1 | 1 | 776 | 0 | 0.0013 | T | 0.0013 |  |
| chr11 | NA | 75015247 | G | T | 1 | 1 | 1 | 776 | 0 | 0.0013 | T | 0.0013 |  |
| chr11 | NA | 75015380 | T | G | 1 | 1 | 1 | 776 | 0 | 0.0013 | G | 0.0013 |  |
| chr11 | NA | 75015824 | C | G | 1 | 1 | 1 | 776 | 0 | 0.0013 | G | 0.0013 |  |
| chr11 | NA | 75015917 | G | A | 1 | 1 | 1 | 776 | 0 | 0.0013 | A | 0.0013 |  |
| chr11 | NA | 75041967 | A | G | 1 | 1 | 1 | 776 | 0 | 0.0013 | G | 0.0013 |  |
| chr11 | NA | 75042859 | G | A | 1 | 1 | 1 | 776 | 0 | 0.0013 | A | 0.0013 |  |
| chr11 | NA | 75042904 | A | G | 1 | 1 | 1 | 776 | 0 | 0.0013 | G | 0.0013 |  |
| chr11 | NA | 75019411 | C | T | 1 | 1 | 1 | 776 | 0 | 0.0013 | T | 0.0013 |  |
| chr11 | NA | 75021006 | C | G | 1 | 1 | 1 | 776 | 0 | 0.0013 | G | 0.0013 |  |
| chr11 | NA | 75046001 | A | T | 1 | 1 | 1 | 776 | 0 | 0.0013 | T | 0.0013 |  |
| chr11 | NA | 75046916 | G | A | 1 | 1 | 1 | 776 | 0 | 0.0013 | A | 0.0013 |  |
| chr11 | NA | 75022530 | T | C | 1 | 1 | 1 | 776 | 0 | 0.0013 | C | 0.0013 |  |
| chr11 | NA | 75047180 | C | T | 1 | 1 | 1 | 776 | 0 | 0.0013 | T | 0.0013 |  |
| chr11 | NA | 75023049 | G | A | 1 | 1 | 1 | 776 | 0 | 0.0013 | A | 0.0013 |  |
| chr11 | NA | 75047807 | T | C | 1 | 1 | 1 | 776 | 0 | 0.0013 | C | 0.0013 |  |
| chr11 | NA | 75048078 | C | T | 1 | 1 | 1 | 776 | 0 | 0.0013 | T | 0.0013 |  |
| chr11 | NA | 75024095 | G | A | 1 | 1 | 1 | 776 | 0 | 0.0013 | A | 0.0013 |  |
| chr11 | NA | 75048621 | A | G | 1 | 1 | 1 | 776 | 0 | 0.0013 | G | 0.0013 |  |
| chr11 | NA | 75049157 | G | T | 1 | 1 | 1 | 776 | 0 | 0.0013 | T | 0.0013 |  |
| chr11 | NA | 75049235 | T | C | 1 | 1 | 1 | 776 | 0 | 0.0013 | C | 0.0013 |  |
| chr11 | NA | 75049510 | G | A | 1 | 1 | 1 | 776 | 0 | 0.0013 | A | 0.0013 |  |
| chr11 | NA | 75025148 | C | T | 1 | 1 | 1 | 776 | 0 | 0.0013 | T | 0.0013 |  |
| chr11 | NA | 75049877 | C | T | 1 | 1 | 1 | 776 | 0 | 0.0013 | T | 0.0013 |  |
| chr11 | NA | 75050557 | G | T | 1 | 1 | 1 | 776 | 0 | 0.0013 | T | 0.0013 |  |
| chr11 | NA | 75050697 | G | T | 1 | 1 | 1 | 776 | 0 | 0.0013 | T | 0.0013 |  |
| chr11 | NA | 75025447 | G | A | 1 | 1 | 1 | 776 | 0 | 0.0013 | A | 0.0013 |  |
| chr11 | NA | 75050952 | G | A | 1 | 1 | 1 | 776 | 0 | 0.0013 | A | 0.0013 |  |
| chr11 | NA | 75051288 | C | G | 1 | 1 | 1 | 776 | 0 | 0.0013 | G | 0.0013 |  |
| chr11 | NA | 75051611 | GTCTT | G | 1 | 1 | 1 | 776 | 0 | 0.0013 | G | 0.0013 |  |
| chr11 | NA | 75027368 | CAA | C | 1 | 1 | 1 | 776 | 0 | 0.0013 | C | 0.0013 |  |
| chr11 | NA | 75052026 | G | A | 1 | 1 | 1 | 776 | 0 | 0.0013 | A | 0.0013 |  |
| chr11 | NA | 75052185 | TTTG | T | 1 | 1 | 1 | 776 | 0 | 0.0013 | T | 0.0013 |  |
| chr11 | NA | 75052237 | A | G | 1 | 1 | 1 | 776 | 0 | 0.0013 | G | 0.0013 |  |
| chr11 | NA | 75052472 | A | G | 1 | 1 | 1 | 776 | 0 | 0.0013 | G | 0.0013 |  |
| chr11 | NA | 75028514 | G | C | 1 | 1 | 1 | 776 | 0 | 0.0013 | C | 0.0013 |  |
| chr11 | NA | 75028553 | G | A | 1 | 1 | 1 | 776 | 0 | 0.0013 | A | 0.0013 |  |
| chr11 | NA | 75029730 | T | C | 1 | 1 | 1 | 776 | 0 | 0.0013 | C | 0.0013 |  |
| chr11 | NA | 75029963 | G | A | 1 | 1 | 1 | 776 | 0 | 0.0013 | A | 0.0013 |  |
| chr11 | NA | 75030014 | C | T | 1 | 1 | 1 | 776 | 0 | 0.0013 | T | 0.0013 |  |
| chr11 | NA | 75030338 | G | A | 1 | 1 | 1 | 776 | 0 | 0.0013 | A | 0.0013 |  |
| chr11 | NA | 75030922 | C | G | 1 | 1 | 1 | 776 | 0 | 0.0013 | G | 0.0013 |  |
| chr11 | NA | 75030940 | G | A | 1 | 1 | 1 | 776 | 0 | 0.0013 | A | 0.0013 |  |
| chr11 | NA | 75031030 | T | G | 1 | 1 | 1 | 776 | 0 | 0.0013 | G | 0.0013 |  |
| chr11 | NA | 75031054 | C | T | 1 | 1 | 1 | 776 | 0 | 0.0013 | T | 0.0013 |  |
| chr11 | NA | 75031197 | A | G | 1 | 1 | 1 | 776 | 0 | 0.0013 | G | 0.0013 |  |
| chr11 | NA | 75031482 | C | T | 1 | 1 | 1 | 776 | 0 | 0.0013 | T | 0.0013 |  |
| chr11 | NA | 75039857 | A | G | 1 | 1 | 1 | 776 | 0 | 0.0013 | G | 0.0013 |  |
| chr11 | NA | 75032081 | C | CT | 1 | 1 | 1 | 776 | 0 | 0.0013 | CT | 0.0013 |  |
| chr11 | NA | 75032312 | C | A | 1 | 1 | 1 | 776 | 0 | 0.0013 | A | 0.0013 |  |
| chr11 | NA | 75032425 | G | C | 1 | 1 | 1 | 776 | 0 | 0.0013 | C | 0.0013 |  |
| chr11 | NA | 75033558 | AAAC | A | 1 | 1 | 1 | 776 | 0 | 0.0013 | A | 0.0013 |  |
| chr11 | NA | 75033857 | A | G | 1 | 1 | 1 | 776 | 0 | 0.0013 | G | 0.0013 |  |
| chr11 | NA | 75033929 | C | G | 1 | 1 | 1 | 776 | 0 | 0.0013 | G | 0.0013 |  |
| chr11 | NA | 75034063 | A | T | 1 | 1 | 1 | 776 | 0 | 0.0013 | T | 0.0013 |  |
| chr11 | NA | 75035263 | T | G | 1 | 1 | 1 | 776 | 0 | 0.0013 | G | 0.0013 |  |
| chr11 | NA | 75035362 | C | T | 1 | 1 | 1 | 776 | 0 | 0.0013 | T | 0.0013 |  |
| chr11 | NA | 75035549 | G | A | 1 | 1 | 1 | 776 | 0 | 0.0013 | A | 0.0013 |  |
| chr11 | NA | 75035597 | A | T | 1 | 1 | 1 | 776 | 0 | 0.0013 | T | 0.0013 |  |
| chr11 | NA | 75035651 | G | A | 1 | 1 | 1 | 776 | 0 | 0.0013 | A | 0.0013 |  |
| chr11 | NA | 75035741 | A | G | 1 | 1 | 1 | 776 | 0 | 0.0013 | G | 0.0013 |  |
| chr11 | NA | 75035934 | C | A | 1 | 1 | 1 | 776 | 0 | 0.0013 | A | 0.0013 |  |
| chr11 | NA | 75035935 | C | G | 1 | 1 | 1 | 776 | 0 | 0.0013 | G | 0.0013 |  |
| chr11 | NA | 75007229 | C | CAAAAA | 1 | 1 | 1 | 776 | 0 | 0.0013 | CAAAAA | 0.0013 |  |
| chr11 | NA | 75036104 | C | CGTA | 1 | 1 | 1 | 776 | 0 | 0.0013 | CGTA | 0.0013 |  |
| chr11 | NA | 75036929 | C | T | 1 | 1 | 1 | 776 | 0 | 0.0013 | T | 0.0013 |  |
| chr11 | NA | 75008956 | A | G | 1 | 1 | 1 | 776 | 0 | 0.0013 | G | 0.0013 |  |
| chr11 | NA | 75009325 | T | C | 1 | 1 | 1 | 776 | 0 | 0.0013 | C | 0.0013 |  |
| chr11 | NA | 75011285 | A | C | 1 | 1 | 1 | 776 | 0 | 0.0013 | C | 0.0013 |  |
| chr11 | NA | 75011332 | TGTAA | T | 1 | 1 | 1 | 776 | 0 | 0.0013 | T | 0.0013 |  |
| chr11 | NA | 75039493 | T | C | 1 | 1 | 1 | 776 | 0 | 0.0013 | C | 0.0013 |  |
| chr11 | NA | 75055415 | G | T | 1 | 1 | 1 | 776 | 0 | 0.0013 | T | 0.0013 |  |
| chr11 | NA | 75040019 | AAG | A | 1 | 1 | 1 | 776 | 0 | 0.0013 | A | 0.0013 |  |
| chr11 | NA | 75040020 | A | G | 1 | 1 | 1 | 776 | 0 | 0.0013 | G | 0.0013 |  |
| chr11 | NA | 75040116 | C | T | 1 | 1 | 1 | 776 | 0 | 0.0013 | T | 0.0013 |  |
| chr11 | NA | 75040121 | C | T | 1 | 1 | 1 | 776 | 0 | 0.0013 | T | 0.0013 |  |
| chr11 | NA | 75040629 | G | A | 1 | 1 | 1 | 776 | 0 | 0.0013 | A | 0.0013 |  |
| chr11 | NA | 75040630 | C | T | 1 | 1 | 1 | 776 | 0 | 0.0013 | T | 0.0013 |  |
| chr11 | NA | 75040703 | A | G | 1 | 1 | 1 | 776 | 0 | 0.0013 | G | 0.0013 |  |
| chr11 | NA | 75041134 | G | A | 1 | 1 | 1 | 776 | 0 | 0.0013 | A | 0.0013 |  |
| chr11 | NA | 75041600 | C | T | 1 | 1 | 1 | 776 | 0 | 0.0013 | T | 0.0013 |  |
| chr11 | NA | 75034771 | G | A | 1 | 1 | 1 | 776 | 0 | 0.0013 | A | 0.0013 |  |
| chr11 | NA | 75051352 | T | C | 1 | 1 | 1 | 776 | 0 | 0.0013 | C | 0.0013 |  |
| chr11 | NA | 75052025 | C | T | 1 | 1 | 1 | 776 | 0 | 0.0013 | T | 0.0013 |  |
| chr11 | NA | 75059924 | G | A | 1 | 1 | 1 | 776 | 0 | 0.0013 | A | 0.0013 |  |
| chr11 | NA | 75044000 | T | C | 1 | 1 | 1 | 776 | 0 | 0.0013 | C | 0.0013 |  |
| chr11 | NA | 75019876 | G | A | 1 | 1 | 1 | 776 | 0 | 0.0013 | A | 0.0013 |  |
| chr11 | NA | 75044952 | C | T | 1 | 1 | 1 | 776 | 0 | 0.0013 | T | 0.0013 |  |
| chr11 | NA | 75053454 | C | T | 1 | 1 | 1 | 776 | 0 | 0.0013 | T | 0.0013 |  |
| chr11 | NA | 75045528 | C | G | 1 | 1 | 1 | 776 | 0 | 0.0013 | G | 0.0013 |  |
| chr11 | NA | 75029403 | A | G | 1 | 1 | 1 | 776 | 0 | 0.0013 | G | 0.0013 |  |
| chr11 | NA | 75054118 | C | T | 1 | 1 | 1 | 776 | 0 | 0.0013 | T | 0.0013 |  |
| chr11 | NA | 75021445 | A | G | 1 | 1 | 1 | 776 | 0 | 0.0013 | G | 0.0013 |  |
| chr11 | NA | 75054258 | T | TGA | 1 | 1 | 1 | 776 | 0 | 0.0013 | TGA | 0.0013 |  |
| chr11 | NA | 75054439 | CAAA | C | 1 | 1 | 1 | 776 | 0 | 0.0013 | C | 0.0013 |  |
| chr11 | NA | 75054640 | G | A | 1 | 1 | 1 | 776 | 0 | 0.0013 | A | 0.0013 |  |
| chr11 | NA | 75047410 | T | A | 1 | 1 | 1 | 776 | 0 | 0.0013 | A | 0.0013 |  |
| chr11 | NA | 75054763 | T | C | 1 | 1 | 1 | 776 | 0 | 0.0013 | C | 0.0013 |  |
| chr11 | NA | 75048530 | T | G | 1 | 1 | 1 | 776 | 0 | 0.0013 | G | 0.0013 |  |
| chr11 | NA | 75058534 | T | C | 1 | 1 | 1 | 776 | 0 | 0.0013 | C | 0.0013 |  |
| chr11 | NA | 75055770 | C | T | 1 | 1 | 1 | 776 | 0 | 0.0013 | T | 0.0013 |  |
| chr11 | NA | 75055811 | C | T | 1 | 1 | 1 | 776 | 0 | 0.0013 | T | 0.0013 |  |
| chr11 | NA | 75049658 | A | T | 1 | 1 | 1 | 776 | 0 | 0.0013 | T | 0.0013 |  |
| chr11 | NA | 75050395 | C | T | 1 | 1 | 1 | 776 | 0 | 0.0013 | T | 0.0013 |  |
| chr11 | NA | 75057505 | C | T | 1 | 1 | 1 | 776 | 0 | 0.0013 | T | 0.0013 |  |
| chr11 | NA | 75058184 | G | A | 1 | 1 | 1 | 776 | 0 | 0.0013 | A | 0.0013 |  |
| chr11 | NA | 75060423 | A | G | 1 | 1 | 1 | 776 | 0 | 0.0013 | G | 0.0013 |  |
| chr11 | NA | 75060772 | C | T | 1 | 1 | 1 | 776 | 0 | 0.0013 | T | 0.0013 |  |
| chr11 | NA | 75035084 | G | GCA | 1 | 1 | 1 | 776 | 0 | 0.0013 | GCA | 0.0013 |  |
| chr11 | NA | 75059345 | T | A | 1 | 1 | 1 | 776 | 0 | 0.0013 | A | 0.0013 |  |
| chr11 | NA | 75059543 | G | A | 1 | 1 | 1 | 776 | 0 | 0.0013 | A | 0.0013 |  |
| chr11 | NA | 75054381 | G | A | 1 | 1 | 1 | 776 | 0 | 0.0013 | A | 0.0013 |  |
| chr11 | NA | 75061284 | C | T | 1 | 1 | 1 | 776 | 0 | 0.0013 | T | 0.0013 |  |
| chr11 | NA | 75059962 | C | T | 1 | 1 | 1 | 776 | 0 | 0.0013 | T | 0.0013 |  |
| chr11 | NA | 75060052 | G | A | 1 | 1 | 1 | 776 | 0 | 0.0013 | A | 0.0013 |  |
| chr11 | NA | 75052774 | C | T | 1 | 1 | 1 | 776 | 0 | 0.0013 | T | 0.0013 |  |
| chr11 | NA | 75060248 | G | A | 1 | 1 | 1 | 776 | 0 | 0.0013 | A | 0.0013 |  |
| chr11 | NA | 75055380 | T | C | 1 | 1 | 1 | 776 | 0 | 0.0013 | C | 0.0013 |  |
| chr11 | NA | 75060355 | TG | T | 1 | 1 | 1 | 776 | 0 | 0.0013 | T | 0.0013 |  |
| chr11 | NA | 75054139 | C | T | 1 | 1 | 1 | 776 | 0 | 0.0013 | T | 0.0013 |  |
| chr11 | NA | 75037796 | G | A | 1 | 1 | 1 | 776 | 0 | 0.0013 | A | 0.0013 |  |
| chr11 | NA | 75061571 | C | A | 1 | 1 | 1 | 776 | 0 | 0.0013 | A | 0.0013 |  |
| chr11 | NA | 75054653 | T | A | 1 | 1 | 1 | 776 | 0 | 0.0013 | A | 0.0013 |  |
| chr11 | NA | 75062460 | C | A | 1 | 1 | 1 | 776 | 0 | 0.0013 | A | 0.0013 |  |
| chr11 | NA | 75036036 | C | G | 1 | 1 | 1 | 776 | 0 | 0.0013 | G | 0.0013 |  |
| chr11 | NA | 75058597 | C | T | 1 | 1 | 1 | 776 | 0 | 0.0013 | T | 0.0013 |  |
| chr11 | NA | 75056342 | C | T | 1 | 1 | 1 | 776 | 0 | 0.0013 | T | 0.0013 |  |
| chr11 | NA | 75037749 | G | GC | 1 | 1 | 1 | 776 | 0 | 0.0013 | GC | 0.0013 |  |
| chr11 | NA | 75037346 | G | T | 1 | 1 | 1 | 776 | 0 | 0.0013 | T | 0.0013 |  |
| chr11 | NA | 75044651 | C | T | 1 | 1 | 1 | 776 | 0 | 0.0013 | T | 0.0013 |  |
| chr11 | NA | 75061123 | A | C | 1 | 1 | 1 | 776 | 0 | 0.0013 | C | 0.0013 |  |

**Supplementary Table 2: Sociodemographic characteristics according to genotype**

Sociodemographic characteristics of METADAP patients are shown according to genotype for each of the 9 prioritized SNPs (i.e., MAF≥5% and RegulomeDB category rank of 1 or 2). Kruskal-Wallis tests were used to compare age, tobacco consumption, onset age of MDE, and baseline HDRS scores (presented as mean±standard deviation). Fisher Exact tests were used to compare sex, socio-education status, ethnicity, smoking status at baseline, MDE recurrence, prescribed AD drug, and dropout rates across study time (presented as the number of patients and percentage). *: *P*<0.05; **: *P*<0.01; ***: *P*<0.001. **AD**: antidepressant drug; **ECT**: electroconvulsive therapy; **HDRS**: 17-item Hamilton Depression Rating Scale; **m**: mean; **M1**: after 1 month of treatment; **M3**: after 3 months of treatment; **M6**: after 6 months of treatment; **MDE**: major depressive episode; **n**: number of patients; ***P***: *P*-value; **SNRI**: serotonin norepinephrine reuptake inhibitor; **sd**: standard deviation; **SSRI**: selective serotonin reuptake inhibitor; **TCA**: tricyclic antidepressant

|  |  | | | Total | | | rs2279130 | | | | | | | | | | | | rs501372 | | | | | | | | | | | | rs877711 | | | | | | | | |
| --- | --- | --- | --- | --- | --- | --- | --- | --- | --- | --- | --- | --- | --- | --- | --- | --- | --- | --- | --- | --- | --- | --- | --- | --- | --- | --- | --- | --- | --- | --- | --- | --- | --- | --- | --- | --- | --- | --- | --- |
|  |  | | |  | | | CC | | | CT | | | TT | | | *P* | | | CC | | | CA | | | AA | | | *P* | | | GG | | | GA | | AA | | *P* | |
|  |  | | | n=388 | | | n=334 | | | n=50 | | | n=3 | | |  | | | n=134 | | | n=194 | | | n=54 | | |  | | | n=303 | | | n=75 | | n=8 | |  | |
| Age (m±sd) |  | | | 45.4±13.3 | | | 45±13.1 | | | 47.3±14 | | | 57.7±18.3 | | | 0.14 | | | 44.5±12.9 | | | 45.7±13.3 | | | 47.3±14.8 | | | 0.37 | | | 45.7±13.3 | | | 45.5±13.2 | | 38.9±16.2 | | 0.51 | |
| Female (n(%)) |  | | | 263(68) | | | 224(67) | | | 36(72) | | | 2(67) | | | 0.79 | | | 87(65) | | | 127(65) | | | 45(83) | | | **0.027*** | | | 199(66) | | | 55(73) | | 7(88) | | 0.25 | |
| Education (n(%)) | *Primary* | | | 36(9) | | | 30(9) | | | 5(10) | | | 0(0) | | | 0.16 | | | 13(10) | | | 16(8) | | | 7(13) | | | 0.40 | | | 30(10) | | | 6(8) | | 0(0) | | 0.86 | |
|  | *High school* | | | 168(43) | | | 144(43) | | | 21(42) | | | 3(100) | | |  | | | 51(38) | | | 88(45) | | | 27(50) | | |  | | | 128(42) | | | 35(47) | | 5(62) | |  | |
|  | *University* | | | 183(47) | | | 160(48) | | | 23(46) | | | 0(0) | | |  | | | 70(52) | | | 89(46) | | | 20(37) | | |  | | | 144(48) | | | 34(45) | | 3(38) | |  | |
| Ethnicity (n(%)) | *Caucasian* | | | 353(91) | | | 305(91) | | | 44(88) | | | 3(100) | | | 0.53 | | | 126(94) | | | 177(91) | | | 45(83) | | | **0.0023*** | | | 276(91) | | | 67(89) | | 8(100) | | 0.84 | |
|  | *African* | | | 24(6) | | | 19(6) | | | 5(10) | | | 0(0) | | |  | | | 7(5) | | | 7(4) | | | 9(17) | | |  | | | 18(6) | | | 6(8) | | 0(0) | |  | |
|  | *Mixed* | | | 10(3) | | | 9(3) | | | 1(2) | | | 0(0) | | |  | | | 1(1) | | | 9(5) | | | 0(0) | | |  | | | 9(3) | | | 1(1) | | 0(0) | |  | |
| Current smoker (n(%)) |  | | | 149(38) | | | 129(39) | | | 19(38) | | | 1(33) | | | 1.00 | | | 51(38) | | | 72(37) | | | 23(43) | | | 0.76 | | | 112(37) | | | 31(41) | | 5(62) | | 0.27 | |
| Pack years (m±sd) |  | | | 15.2(14.9) | | | 14.8±14.1 | | | 18.2±19.6 | | | 2±NA | | | 0.37 | | | 13.9±15.8 | | | 15.6±14.8 | | | 15.6±13.4 | | | 0.52 | | | 15.8±15.5 | | | 12.5±10.1 | | 18.2±21.7 | | 0.69 | |
| Recurrent MDE (n(%)) |  | | | 284(73) | | | 242(72) | | | 39(78) | | | 2(67) | | | 0.58 | | | 91(68) | | | 150(77) | | | 40(74) | | | 0.16 | | | 225(74) | | | 53(71) | | 5(62) | | 0.55 | |
| Onset age MDE (m±sd)) |  | | | 35.3±14.5 | | | 35±14.3 | | | 37.3±15.6 | | | 28.7±18.9 | | | 0.50 | | | 34.3±14 | | | 35.6±14.7 | | | 36.4±15.2 | | | 0.54 | | | 35.2±14.7 | | | 35.8±13.6 | | 32.9±15.9 | | 0.79 | |
| Baseline HDRS (m±sd)) |  | | | 24.8±4.9 | | | 24.8±4.9 | | | 24.5±4.7 | | | 21.3±0.6 | | | 0.41 | | | 24.8±4.8 | | | 24.9±5 | | | 24.2±4.8 | | | 0.70 | | | 25.2±4.9 | | | 23.4±4.8 | | 22.5±4.7 | | **0.0049*** | |
| Prescribed AD (n(%)) | *SSRI* | | | 157(40) | | | 140(42) | | | 16(32) | | | 1(33) | | | **0.0081*** | | | 57(43) | | | 76(39) | | | 24(44) | | | 0.38 | | | 121(40) | | | 29(39) | | 6(75) | | 0.23 | |
|  | *SNRI* | | | 158(41) | | | 140(42) | | | 16(32) | | | 1(33) | | |  | | | 53(40) | | | 85(44) | | | 16(30) | | |  | | | 127(42) | | | 30(40) | | 0(0) | |  | |
|  | *TCA* | | | 24(6) | | | 21(6) | | | 3(6) | | | 0(0) | | |  | | | 6(4) | | | 14(7) | | | 3(6) | | |  | | | 18(6) | | | 6(8) | | 0(0) | |  | |
|  | *Other* | | | 34(9) | | | 24(7) | | | 9(18) | | | 1(33) | | |  | | | 11(8) | | | 14(7) | | | 8(15) | | |  | | | 25(8) | | | 7(9) | | 2(25) | |  | |
|  | *ECT* | | | 15(4) | | | 9(3) | | | 6(12) | | | 0(0) | | |  | | | 7(5) | | | 5(3) | | | 3(6) | | |  | | | 12(4) | | | 3(4) | | 0(0) | |  | |
| Dropout (n(%)) | *M1* | | | 21(5) | | | 18(5) | | | 3(6) | | | 0(0) | | | 0.78 | | | 8(6) | | | 9(5) | | | 4(7) | | | 0.61 | | | 14(5) | | | 7(9) | | 0(0) | | 0.24 | |
|  | *M3* | | | 121(31) | | | 102(31) | | | 17(34) | | | 2(67) | | | 0.32 | | | 39(29) | | | 59(30) | | | 22(41) | | | 0.27 | | | 92(30) | | | 23(31) | | 5(62) | | 0.18 | |
|  | *M6* | | | 179(46) | | | 151(45) | | | 25(50) | | | 2(67) | | | 0.62 | | | 55(41) | | | 97(50) | | | 26(48) | | | 0.27 | | | 134(44) | | | 39(52) | | 5(62) | | 0.32 | |
|  | |  | | | rs553664 | | | | | | | | | | | | | rs536852 | | | | | | | | | | | | rs1676887 | | | | | | | | | |
|  | |  | | | GG | | | GA | | | AA | | | | *P* | | | GG | | | GA | | | AA | | | *P* | | | GG | | | GA | | | | AA | | *P* |
|  | |  | | | n=115 | | | n=181 | | | n=89 | | | |  | | | n=106 | | | n=190 | | | n=88 | | |  | | | n=310 | | | n=68 | | | | n=6 | |  |
| Age (m±sd) | |  | | | 44.4±13.5 | | | 46.7±13.2 | | | 44.3±13.4 | | | | 0.23 | | | 45.3±13.5 | | | 46.9±13.2 | | | 42.6±13.1 | | | **0.041*** | | | 45.3±13.2 | | | 46.6±13.5 | | | | 37.7±16.1 | | 0.37 |
| Female (n(%)) | |  | | | 70(61) | | | 126(70) | | | 64(72) | | | | 0.18 | | | 77(73) | | | 130(68) | | | 52(59) | | | 0.13 | | | 205(66) | | | 49(72) | | | | 5(83) | | 0.52 |
| Education (n(%)) | | *Primary* | | | 10(9) | | | 13(7) | | | 13(15) | | | | 0.21 | | | 14(13) | | | 15(8) | | | 7(8) | | | 0.34 | | | 29(9) | | | 7(10) | | | | 0(0) | | 0.99 |
|  | | *High school* | | | 44(38) | | | 83(46) | | | 39(44) | | | |  | | | 51(48) | | | 78(41) | | | 37(42) | | |  | | | 134(43) | | | 31(46) | | | | 3(50) | |  |
|  | | *University* | | | 60(52) | | | 85(47) | | | 37(42) | | | |  | | | 41(39) | | | 96(51) | | | 44(50) | | |  | | | 146(47) | | | 30(44) | | | | 3(50) | |  |
| Ethnicity (n(%)) | | *Caucasian* | | | 111(97) | | | 166(92) | | | 73(82) | | | | **0.0001*** | | | 92(87) | | | 173(91) | | | 85(97) | | | **0.014*** | | | 280(90) | | | 63(93) | | | | 6(100) | | 0.96 |
|  | | *African* | | | 0(0) | | | 12(7) | | | 12(13) | | | |  | | | 11(10) | | | 12(6) | | | 0(0) | | |  | | | 20(6) | | | 4(6) | | | | 0(0) | |  |
|  | | *Mixed* | | | 4(3) | | | 2(1) | | | 4(4) | | | |  | | | 3(3) | | | 4(2) | | | 3(3) | | |  | | | 9(3) | | | 1(1) | | | | 0(0) | |  |
| Current smoker (n(%)) | |  | | | 55(48) | | | 57(31) | | | 35(39) | | | | **0.018*** | | | 40(38) | | | 64(34) | | | 44(50) | | | **0.035*** | | | 113(36) | | | 28(41) | | | | 5(83) | | 0.054 |
| Pack years (m±sd) | |  | | | 11.9±10.9 | | | 18.4±18.5 | | | 13.5±10.8 | | | | 0.08 | | | 16.2±15.7 | | | 15.9±16.1 | | | 12.7±11.4 | | | 0.48 | | | 15.3±15.5 | | | 15.6±13.1 | | | | 7.8±5.8 | | 0.40 |
| Recurrent MDE (n(%)) | |  | | | 84(73) | | | 131(72) | | | 67(75) | | | | 0.89 | | | 82(77) | | | 136(72) | | | 63(72) | | | 0.54 | | | 224(72) | | | 52(76) | | | | 5(83) | | 0.74 |
| Onset age MDE (m±sd)) | |  | | | 34.1±13.8 | | | 36.1±14.6 | | | 35.1±15.2 | | | | 0.52 | | | 35.1±14.8 | | | 36.7±14.7 | | | 32.3±13.3 | | | 0.06 | | | 35.3±14.7 | | | 35.7±13.5 | | | | 33.5±17.7 | | 0.87 |
| Baseline HDRS (m±sd)) | |  | | | 23.6±4.4 | | | 25.2±4.7 | | | 25.2±5.4 | | | | **0.025*** | | | 25.3±5.2 | | | 24.9±4.8 | | | 24±4.7 | | | 0.20 | | | 25.1±5 | | | 23.5±4.4 | | | | 23.7±5 | | 0.06 |
| Prescribed AD (n(%)) | | *SSRI* | | | 46(40) | | | 70(39) | | | 41(46) | | | | 0.93 | | | 46(43) | | | 72(38) | | | 39(44) | | | 0.66 | | | 124(40) | | | 27(40) | | | | 5(83) | | 0.81 |
|  | | *SNRI* | | | 48(42) | | | 77(43) | | | 30(34) | | | |  | | | 35(33) | | | 84(44) | | | 35(40) | | |  | | | 127(41) | | | 27(40) | | | | 1(17) | |  |
|  | | *TCA* | | | 6(5) | | | 11(6) | | | 7(8) | | | |  | | | 9(8) | | | 10(5) | | | 5(6) | | |  | | | 20(6) | | | 4(6) | | | | 0(0) | |  |
|  | | *Other* | | | 11(10) | | | 16(9) | | | 7(8) | | | |  | | | 10(9) | | | 18(9) | | | 6(7) | | |  | | | 28(9) | | | 6(9) | | | | 0(0) | |  |
|  | | *ECT* | | | 4(3) | | | 7(4) | | | 4(4) | | | |  | | | 6(6) | | | 6(3) | | | 3(3) | | |  | | | 11(4) | | | 4(6) | | | | 0(0) | |  |
| Dropout (n(%)) | | *M1* | | | 8(7) | | | 8(4) | | | 5(6) | | | | 0.62 | | | 6(6) | | | 9(5) | | | 6(7) | | | 0.73 | | | 14(5) | | | 6(9) | | | | 0(0) | | 0.44 |
|  | | *M3* | | | 29(25) | | | 61(34) | | | 30(34) | | | | 0.26 | | | 36(34) | | | 59(31) | | | 24(27) | | | 0.61 | | | 90(29) | | | 27(40) | | | | 2(33) | | 0.22 |
|  | | *M6* | | | 54(47) | | | 80(44) | | | 44(49) | | | | 0.71 | | | 50(47) | | | 85(45) | | | 42(48) | | | 0.87 | | | 137(44) | | | 39(57) | | | | 2(33) | | 0.11 |
|  | | |  | | | rs113636971 | | | | | | | | | | | rs504683 | | | | | | | | | | | | rs561923 | | | | | | | | | | |
|  | | |  | | | AA | | | AG | | | GG | | *P* | | | AA | | | AG | | | GG | | | *P* | | | CC | | | CA | | | AA | | | *P* | |
|  | | |  | | | n=340 | | | n=46 | | | n=2 | |  | | | n=195 | | | n=164 | | | n=27 | | |  | | | n=220 | | | n=142 | | | n=20 | | |  | |
| Age (m±sd) | | |  | | | 45.4±13.3 | | | 45.6±13.9 | | | 43±8.5 | | 0.94 | | | 44.4±13.4 | | | 46.3±13 | | | 47.4±14.3 | | | 0.30 | | | 44.1±13.6 | | | 47±12.5 | | | 50.1±15.1 | | | 0.052 | |
| Female (n(%)) | | |  | | | 233(69) | | | 29(63) | | | 1(50) | | 0.51 | | | 134(69) | | | 108(66) | | | 20(74) | | | 0.68 | | | 150(68) | | | 94(66) | | | 14(70) | | | 0.90 | |
| Education (n(%)) | | | *Primary* | | | 32(9) | | | 4(9) | | | 0(0) | | 0.83 | | | 17(9) | | | 16(10) | | | 2(7) | | | 0.95 | | | 20(9) | | | 15(11) | | | 1(5) | | | 0.94 | |
|  | | | *High school* | | | 147(43) | | | 21(46) | | | 0(0) | |  | | | 85(44) | | | 73(45) | | | 10(37) | | |  | | | 91(41) | | | 64(45) | | | 9(45) | | |  | |
|  | | | *University* | | | 160(47) | | | 21(46) | | | 2(100) | |  | | | 92(47) | | | 75(46) | | | 15(56) | | |  | | | 108(49) | | | 63(44) | | | 10(50) | | |  | |
| Ethnicity (n(%)) | | | *Caucasian* | | | 308(91) | | | 43(93) | | | 2(100) | | 0.92 | | | 167(86) | | | 157(96) | | | 27(100) | | | **0.0044*** | | | 192(87) | | | 136(96) | | | 20(100) | | | **0.032*** | |
|  | | | *African* | | | 22(6) | | | 2(4) | | | 0(0) | |  | | | 19(10) | | | 5(3) | | | 0(0) | | |  | | | 19(9) | | | 4(3) | | | 0(0) | | |  | |
|  | | | *Mixed* | | | 9(3) | | | 1(2) | | | 0(0) | |  | | | 9(5) | | | 1(1) | | | 0(0) | | |  | | | 9(4) | | | 1(1) | | | 0(0) | | |  | |
| Current smoker (n(%)) | | |  | | | 126(37) | | | 22(48) | | | 1(50) | | 0.31 | | | 82(42) | | | 59(36) | | | 8(30) | | | 0.32 | | | 90(41) | | | 50(35) | | | 6(30) | | | 0.42 | |
| Pack years (m±sd) | | |  | | | 15.3±15.4 | | | 14±11.3 | | | 20±NA | | 0.72 | | | 14.4±14.5 | | | 16.7±15.1 | | | 13±17 | | | 0.26 | | | 14.3±14.3 | | | 17.6±16.2 | | | 11.1±12.5 | | | 0.24 | |
| Recurrent MDE (n(%)) | | |  | | | 246(72) | | | 36(78) | | | 2(100) | | 0.67 | | | 138(71) | | | 122(74) | | | 23(85) | | | 0.28 | | | 159(72) | | | 105(74) | | | 16(80) | | | 0.80 | |
| Onset age MDE (m±sd)) | | |  | | | 35.7±14.4 | | | 32.5±14.7 | | | 29±4.2 | | 0.39 | | | 35±14 | | | 35.5±15.1 | | | 34.9±14.1 | | | 0.94 | | | 34.7±14 | | | 35.9±14.9 | | | 37±17.3 | | | 0.65 | |
| Baseline HDRS (m±sd)) | | |  | | | 24.8±4.9 | | | 24.6±4.9 | | | 21.5±0.7 | | 0.58 | | | 25.1±5.1 | | | 24.6±4.6 | | | 24.1±5.3 | | | 0.55 | | | 24.9±5.1 | | | 24.5±4.5 | | | 24.6±5.5 | | | 0.85 | |
| Prescribed AD (n(%)) | | | *SSRI* | | | 139(41) | | | 18(39) | | | 0(0) | | 0.08 | | | 85(44) | | | 64(39) | | | 8(30) | | | 0.41 | | | 90(41) | | | 58(41) | | | 7(35) | | | 0.84 | |
|  | | | *SNRI* | | | 139(41) | | | 19(41) | | | 0(0) | |  | | | 72(37) | | | 72(44) | | | 12(44) | | |  | | | 86(39) | | | 60(42) | | | 9(45) | | |  | |
|  | | | *TCA* | | | 19(6) | | | 5(11) | | | 0(0) | |  | | | 12(6) | | | 11(7) | | | 1(4) | | |  | | | 15(7) | | | 8(6) | | | 1(5) | | |  | |
|  | | | *Other* | | | 30(9) | | | 2(4) | | | 2(100) | |  | | | 20(10) | | | 11(7) | | | 3(11) | | |  | | | 22(10) | | | 10(7) | | | 1(5) | | |  | |
|  | | | *ECT* | | | 13(4) | | | 2(4) | | | 0(0) | |  | | | 6(3) | | | 6(4) | | | 3(11) | | |  | | | 7(3) | | | 6(4) | | | 2(10) | | |  | |
| Dropout (n(%)) | | | *M1* | | | 18(5) | | | 3(7) | | | 0(0) | | 0.76 | | | 9(5) | | | 10(6) | | | 2(7) | | | 0.68 | | | 9(4) | | | 10(7) | | | 2(10) | | | 0.21 | |
|  | | | *M3* | | | 110(32) | | | 10(22) | | | 1(50) | | 0.23 | | | 63(32) | | | 48(29) | | | 10(37) | | | 0.65 | | | 79(36) | | | 34(24) | | | 7(35) | | | **0.046*** | |
|  | | | *M6* | | | 158(46) | | | 20(43) | | | 1(50) | | 0.88 | | | 92(47) | | | 74(45) | | | 13(48) | | | 0.91 | | | 108(49) | | | 58(41) | | | 10(50) | | | 0.29 | |
